# Systemically Administered TLR7/8 Agonist and Antigen-Conjugated Nanogels Govern Immune Responses against Tumors

**DOI:** 10.1021/acsnano.1c10709

**Published:** 2022-02-01

**Authors:** Judith Stickdorn, Lara Stein, Danielle Arnold-Schild, Jennifer Hahlbrock, Carolina Medina-Montano, Joschka Bartneck, Tanja Ziß, Evelyn Montermann, Cinja Kappel, Dominika Hobernik, Maximilian Haist, Hajime Yurugi, Marco Raabe, Andreas Best, Krishnaraj Rajalingam, Markus P. Radsak, Sunil A. David, Kaloian Koynov, Matthias Bros, Stephan Grabbe, Hansjörg Schild, Lutz Nuhn

**Affiliations:** † Max Planck Institute for Polymer Research, Ackermannweg 10, 55128 Mainz, Germany; × Institute of Immunology, University Medical Center of Johannes Gutenberg-University Mainz, Langenbeckstraße 1, 55131 Mainz, Germany; § Department of Dermatology, University Medical Center of Johannes Gutenberg-University Mainz, Langenbeckstraße 1, 55131 Mainz, Germany; ∥ III^rd^ Department of Medicine - Hematology, Oncology, Pneumology, University Medical Center of the Johannes Gutenberg-University Mainz, Langenbeckstraße 1, 55131 Mainz, Germany; ⊥ Cell Biology Unit, University Medical Center of Johannes Gutenberg-University Mainz, Langenbeckstraße 1, 55131 Mainz, Germany; # ViroVax, LLC, 2029 Becker Drive Suite 100E, Lawrence 66047-1620, Kansas. United States

**Keywords:** nanogel, vaccine, immunotherapy, block copolymer, TLR agonist, polymer protein conjugate

## Abstract

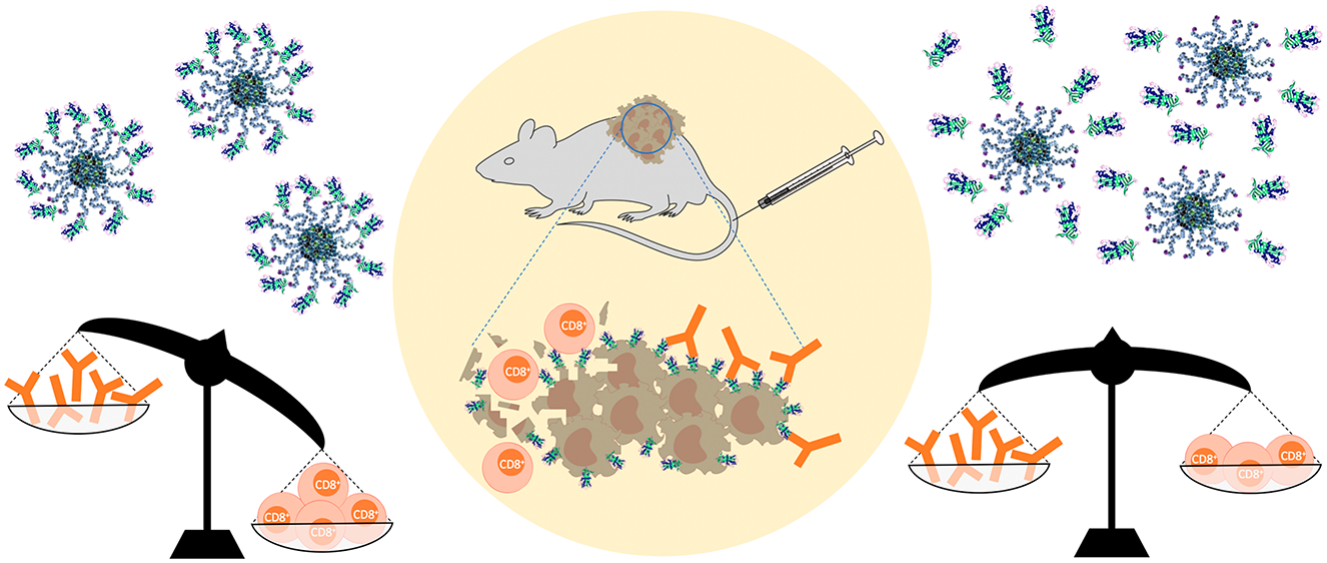

The generation of specific humoral and cellular immune responses plays a pivotal role in the development of effective vaccines against tumors. Especially the presence of antigen-specific, cytotoxic T cells influences the outcome of therapeutic cancer vaccinations. Different strategies, ranging from delivering antigen-encoding mRNAs to peptides or full antigens, are accessible but often suffer from insufficient immunogenicity and require immune-boosting adjuvants as well as carrier platforms to ensure stability and adequate retention. Here, we introduce a pH-responsive nanogel platform as a two-component antitumor vaccine that is safe for intravenous application and elicits robust immune responses *in vitro* and *in vivo*. The underlying chemical design allows for straightforward covalent attachment of a model antigen (ovalbumin) and an immune adjuvant (imidazoquinoline-type TLR7/8 agonist) onto the same nanocarrier system. In addition to eliciting antigen-specific T and B cell responses that outperform mixtures of individual components, our two-component nanovaccine leads in prophylactic and therapeutic studies to an antigen-specific growth reduction of different tumors expressing ovalbumin intracellularly or on their surface. Regarding the versatile opportunities for functionalization, our nanogels are promising for the development of highly customized and potent nanovaccines.

Endeavors in cancer immunotherapy seek to exploit the inherent capacity of immune cells to recognize and neutralize tumor cells. The individual background of each tumor as well as the diversity of each patients’ immune status necessitates the development of personalized antitumor vaccines.^1^ Research accomplishments of the last decades unveiled the role of immune evasion and checkpoint inhibition in tumorigenesis and drew attention to various immune cells as crucial targets in cancer immunotherapy.^2^ To elicit a robust and persistent immune response, vaccines need to trigger an interlude of innate and adaptive immune events both cellularly and humorally.^3^ While the positive contributions of humoral antitumor responses and the function of B cells are currently still discussed,^4^ the effective priming of cytotoxic CD8^+^ T cells against tumor-specific antigens through MHC class I-restricted presentation of antigenic peptides on dendritic cells (DCs), so-called cross-priming,^5^ is of particular importance since the existence of CD8^+^ T cells in the tumor microenvironment can be correlated to improved tumor outcome and patient survival.^6−9^ Recent attempts succeeded by applying antigen encoding mRNAs,^10−12^ however, depending on efficient translation and potent presentation through antigen-presenting cells (APCs).^13,14^ Alternatively, peptide- or protein-based anticancer vaccine strategies have been explored even longer.^15,16^ Unfortunately, they mostly suffer from insufficient immune activation with risk of tolerance^17,18^ and tumor relapse.^19^ Consequently, the development of improved carrier systems and the investigation of potent immune modulators such as Toll-like receptor (TLR) agonists as new vaccine adjuvants are addressed to boost vaccination outcome.^20,21^

Small-molecule imidazoquinoline-based TLR7/8 agonists are promising adjuvant candidates efficiently activating a broad spectrum of APCs, including different types of DCs.^22^ Moreover, they have been demonstrated to trigger high levels of cytokines like TNF-α, INF-γ, IL-6, IL-12, and type I interferon, resulting in Th1-mediated immune responses^23^ and cytotoxic T cells that act against intracellular pathogens or cancer cells.^24^ Nevertheless, clinical use of those small-molecule adjuvants is hampered based on their pharmacokinetic profile causing severe systemic inflammation.^25−27^ Covalent conjugation of TLR7/8 agonists to polymer scaffolds, however, has been shown to amend their safety profile and hinder fast clearance.^28^

The delivery of not only small-molecule adjuvants but also antigenic components including proteins or peptides provides several advantages when combined with polymer-based platforms.^29^ In addition to prolonged circulation and improved pharmacokinetics, nanoparticular systems benefit from accumulation in lymphatic organs and uptake in immune cells based on their morphological and compositional similarities to pathogens.^28,30,31^ If designed properly, they enable co-delivery of adjuvant and antigen to the same DC or other APCs and, thus, foster essential priming of CD8^+^ T cells.^32,33^ Carrier designs range from peptide- or lipid- to synthetic polymer-based systems including self-assembling micelles as well as core-cross-linked structures, which either physically encapsulate or covalently bind the desired cargo.^34−38^ Incorporation of pH-responsive units that trigger disintegration after endosomal uptake seem to enhance antigen presentation and improve priming of CD8^+^ T cells,^35^ in addition to prevent undesired long-term accumulation.^39^ Several studies emphasize that not only co-delivery of adjuvant and antigen to APCs plays a pivotal role in the effectiveness of a vaccine, but also the application of the nanocarrier itself already benefits its efficacy when compared to co-administration of the mixed components without a nanocarrier.^34,36,40^

One straightforward method to obtain amphiphilic, self-assembling polymers for nanocarrier syntheses is the reversible addition–fragmentation chain transfer (RAFT)-polymerization.^41^ RAFT procedures allow the adjustable fabrication of copolymers regarding posterior nanoparticle size and tolerate the introduction of a great variety of functionalities.^42^ While a tailored design of applied chain-transfer agents (CTAs) enables corona modifications, the introduction of reactive monomers allows core-functionalization in post-polymerization reactions.^43−47^ We have combined RAFT-derived heterotelechelic block copolymers with a post-polymerization modification approach^46^ to generate core-cross-linked nanogels^48^ and recently demonstrated that covalent nanogel conjugation with the TLR7/8 agonist 1-(4-(aminomethyl)benzyl)-2-butyl-1*H*-imidazo[4,5-*c*]quinolin-4-amine (IMDQ) leads to localized immune activation after subcutaneous (s.c.) injection with safe antiviral and antitumor immune responses.^30,45,49^

In this study, we report on the evolution of this pH-responsive nanogel platform toward a multi-component antitumor vaccine that is even safe for intravenous (i.v.) administration and facilitates co-delivery of TLR7/8 agonist IMDQ and ovalbumin (OVA) as model antigen for improved generation of CD8^+^ T cells *in vitro*. While the nanogel platform itself is immunologically silent, the nanoparticular co-delivery of IMDQ and OVA induces robust immune responses *in vitro* that outperform soluble mixtures of components. Prophylactic and therapeutic immunization against OVA-expressing tumors revealed the efficacy and specificity of this multi-component vaccine *in vivo*. Based on its chemical design, the herein described nanogel platform allows versatile adjustments regarding the introduced small molecules and attached antigens without morphological changes of the carrier and, therefore, might be an interesting candidate even for personalized anticancer immunotherapies.

## Results and Discussion

### Core and Surface Modification of pH-Degradable Nanogel Platform for Dual Delivery of TLR7/8 Agonist and OVA Antigen

**Figure 1:**
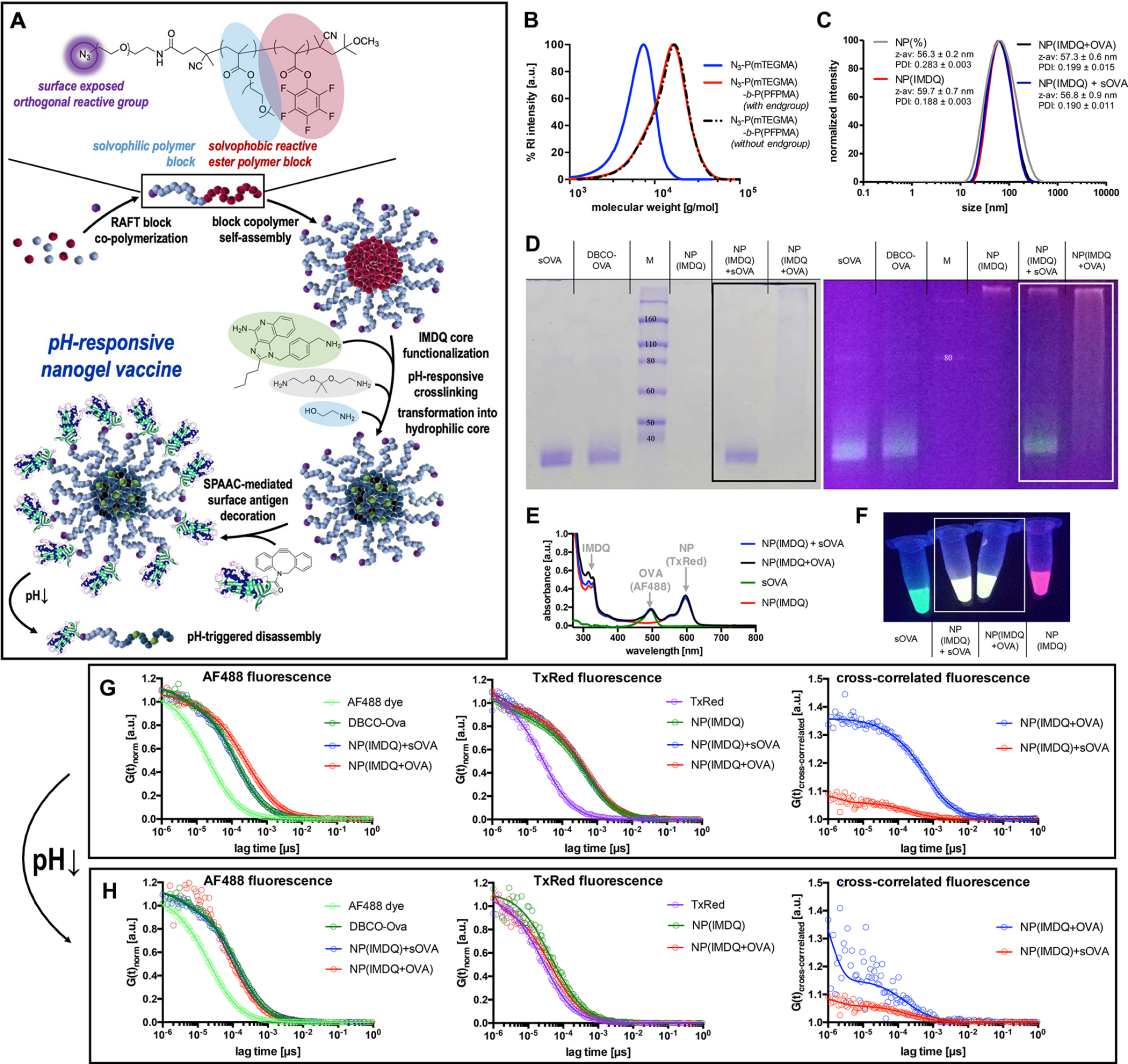
Characterization of TLR7/8-agonist- and protein conjugated nanogels for precise co-delivery of adjuvant and antigen during i.v. antitumor vaccination. (A) Synthetic design concept based on double reactive precursor block copolymers that self-assemble into block copolymer micelles with amine-reactive cores and a SPAAC-reactive corona. *Via* aminolysis of the pentafluorophenyl esters, the cores are covalently functionalized with the TLR 7/8 agonist IMDQ and Texas Red cadaverine and then sequentially cross-linked and transformed into pH-responsive nanogels. The corona is modified *via* click ligation of the surface-exposed azides to DBCO-modified (and Alexa Fluor 488-labeled) OVA as model antigen. (B) SEC chromatography of the RAFT-derived reactive homo and block copolymer (before and after removal of the dithiobenzoate end group). (C) DLS intensity size distribution plots of the resulting nanogels (with and without covalent IMDQ loading), mixed or covalently modified with OVA. (D) SDS-PAGE of modified OVA (labeled with Alexa Fluor 488) mixed or covalently conjugated to IMDQ-loaded nanogels (labeled with Texas Red) (left, Coomassie staining; right, UV excitation of the fluorescent dyes (red, Texas Red-labeled nanogel; green, Alex Fluor 488-labeled OVA). (E) UV–vis spectrum of the fluorescently labeled samples and (F) corresponding image of the samples upon excitation by a UV lamp. (G) FCS correlograms derived from Alexa Fluor 488 and Texas Red fluorescence, as well as their cross-correlated correlogram indicating successful OVA conjugation to the nanogel. (H) FCS correlograms and corresponding cross-correlated correlogram upon exposure to endosomal acidic pH conditions indicating successful particle degradation.

To realize quantifiable co-delivery of antigen and immune stimulant *via* a precise nanoscale carrier, we applied a tunable nanogel platform based on amphiphilic reactive precursor block copolymers that enable both core and corona functionalization *via* post-polymerization modifications. Through covalent conjugations, these nanogels ensure co-delivery of the attached TLR7/8 agonist and OVA as model protein antigen to immune cells both *in vitro* and *in vivo* (Figure 1A).

The underlying block copolymer is accessible through RAFT block copolymerization of methoxy tri(ethylene glycol) methacrylate (mTEGMA) and pentafluorophenyl methacrylate (PFPMA).^47,50^ While the hydrophilic P(mTEGMA) block provides micellar stability and shielding properties, the solvophobic P(PFPMA)-block drives self-assembly to micelles in polar-aprotic solvents like DMSO and facilitates posterior core functionalization of the nanosized precursors by aminolysis of the reactive esters.^51^ When applying the azide-functionalized chain-transfer agent 1-azido-16-cyano-13-oxo-3,6,9-trioxa-12-azaheptadecan-16-yl benzodithioate (azide-CTA) during the RAFT polymerization process,^50^ click-reactive functionalities are installed on the surface of the resulting nanogels allowing for orthogonal surface-modifications through strain-promoted azide–alkyne cycloaddition (SPAAC)^52^ with dibenzyl cyclooctyne (DBCO)-modified counterparts (Figure 1A).

RAFT block copolymerization of mTEGMA and PFPMA using the low-temperature initiator 2,2′-azobis(4-methoxy-2,4-dimethylvaleronitrile (AMDVN) afforded monodisperse block copolymers with a moderate PDI of 1.3 and a number-average molecular weight of around 12 kDa after removal of the dithiobenzoate end groups (Figure 1B; for detailed characterization of P(mTEGMA)_25_ and block copolymer P(mTEGMA)_25_-*b*-P(PFPMA)_34_ compare Figures S1–S10). Formation of reactive precursor micelles after block copolymer self-assembly in DMSO was assessed by dynamic light scattering (DLS) (Figure S12). Subsequent covalent attachment of IMDQ as well as fluorescent labeling with Texas Red dye was achieved through aminolysis of the reactive esters inside the core (Figure 1A). Integrity of the P(mTEGMA) segment during aminolysis of PFP esters was confirmed by ^1^H NMR measurements (Figure S10) in addition to a detailed characterization of the sequential aminolysis of PFP esters by ^19^F NMR monitoring (Figure S9). Further core-cross-linking with the acid-sensitive cross-linker 2,2-bis(aminoethoxy)propane and conversion of the remaining PFP esters into fully hydrophilic moieties by 2-aminoethanol yielded the hydrophilic, pH-labile IMDQ-loaded nanogels that could be purified by dialysis and stored as dry powder after lyophilization. The introduction of the ketal-based cross-linker locks in the nanogels’ morphology and size but at the same time assures disassembly of nanogels into single polymer chains after endosomal uptake.^53^ Particle formation of IMDQ-loaded nanogels as well as their degradation behavior upon an acidic stimulus and long-term stability under physiological conditions were analyzed by DLS and FCS (Figures S13–S15). IMDQ-loading of redispersed nanogels was quantified *via* UV–vis spectroscopy as 9.5 wt% (Figure S11).

In a subsequent experiment, we verified the accessibility of the RAFT CTA-derived, surface-exposed azide groups for SPAAC conjugation. For this purpose, IMDQ-loaded nanogels were incubated with cyclooctyne-modified Oregon Green dye. UV–vis measurements after removal of unbound dye confirmed covalent attachment of the Oregon Green dye and, hence, preserved accessibility of azide-groups for covalent conjugation (Figure S16; a negative control sample of unmodified Oregon Green showed almost complete dye removal). Based on these results, we proceeded with the generation of the envisaged two-component antitumor vaccine composed of IMDQ-nanogels and surface attached model antigen.

Especially for i.v. administration, adjuvants and antigens should be combined into the same carrier system to guarantee co-delivery to the same immune cell subpopulations.^54^ Prior to conjugation of the model antigen OVA to the nanogels, the protein’s lysine-derived amino side chains were exploited through NHS ester chemistry for modification with a SPAAC-reactive DBCO-PEG_4_ linker and an Alexa Fluor 488 label for monitoring (note that OVA is equipped with 20 potential lysine modification sites, and on average one Alexa Fluor 488 and five DBCO-PEG_4_ linkers were attached according to UV–vis spectroscopy; Figure S17). DBCO modification did not alter the integrity or stability of the protein (Figure S18), and it was also well accessible for azide-terminated polymers for conjugation reactions (Figure S19). Interestingly, although one of the modification sites is localized in the CD8^+^ T cell epitope region (compare Supporting Information), DBCO modification did not alter the protein’s antigenicity (Figure S20).

Consequently, covalent conjugation of OVA to the azide-surface exposed nanogels could be performed by simply mixing both components dissolved in PBS, resulting in a protein to azide molar ratio of 1:20. Note that mixing the individually labeled compounds (Texas Red-labeled nanogel and the Alexa Fluor 488-labeled proteins) provided a yellow fluorescent sample (Figure 1E,F and Figure S21) from which successful conjugation could be validated macroscopically by sodium dodecyl sulfate–polyacrylamide gel electrophoresis (SDS-PAGE) (Figure 1D). Soluble ovalbumin (sOVA) as well as DBCO-modified ovalbumin (DBCO-OVA) showed distinct bands after UV excitation as well as after Coomassie staining. The disappearance of unbound DBCO-OVA after SPAAC reaction (NP(IMDQ+OVA)) indicated complete conjugation of DBCO-OVA to azide nanogels NP(IMDQ), while the mixture of sOVA and NP(IMDQ) did not lead to any ligation of sOVA (Figure 1D). In-depth characterization of various protein to azide molar ratios revealed no cross-linking between particles and confirmed the necessity of the excess azide (Figure S24). Further conjugate characterization by DLS revealed monodisperse nanogel formation with sizes around 58 nm that were independent of IMDQ and OVA loading (Figure 1C). Zeta potential measurements revealed a slight decrease in surface charge after conjugation of ovalbumin from −4 to −9.5 mV (Figure S23). Moreover, OVA-bearing particles still showed an acidic degradation profile upon exposure to endosomal pH (Figure S22).

In agreement with the gel electrophoresis experiment, subsequent fluorescence correlation spectroscopy (FCS) measurements^55^ were performed to affirm covalent conjugation of IMDQ-loaded nanogels and DBCO-OVA on a molecular level (Figure 1G). The selection of fluorescent dyes allowed to monitor independently from each other the autocorrelation of the Texas Red-labeled NP(IMDQ) as well as the Alexa Fluor 488-labeled OVA (Figure 1E and Figure S18). Based on this setup, we found for the Alexa Fluor 488-derived OVA autocorrelation a quantitative shift to higher lag times which corresponds to larger species after SPAAC reaction, demonstrating covalent association of OVA to the nanogels (Figure 1G). This shift was independent from IMDQ-loading (compare to FCS measurement of nanogels without IMDQ, Figure S25). *Via* Texas Red-derived nanogel autocorrelation, also a slight increase in size was found (Figure 1G and Figure S26), indicating successful OVA conjugation. In addition, simultaneous analysis of both channels *via* fluorescent cross-correlation spectroscopy (FCCS) revealed an exclusive cross-correlation for NP(IMDQ+OVA) in comparison to the mixture of NP(IMDQ) and sOVA, thus manifesting dual-loading of nanogels also on a molecular level (Figure 1G and Figure S27). Upon acidification, the previously observed cross-correlation dropped and almost disappeared (Figure 1H), reflecting the pH-responsiveness of the nanogels and their disintegration into many non-cross-correlating single polymer chains, as observed also by the Texas Red-derived autocorrelation of NP(IMDQ) before (Figure 1H). Similar results were found again for empty nanogels without IMDQ NP(−) and are summarized in the Supporting Information (Figures S25–S27). Moreover, detailed calculation of the hydrodynamic radii derived from the resulting autocorrelation functions confirmed both conjugation of DBCO-modified OVA to the nanogel as well as degradation of IMDQ-loaded nanogels upon acidification (compare Tables S1 and S2 in the Supporting Information). Summarizing, the underlying chemical design allows for a well-defined and also reproducible fabrication process (compare Figure S28) toward precise co-delivery of immune stimulant and immunogenic part within one nanogel carrier system that is pre-defined in morphology and guarantees control over the co-delivery of each vaccine component.

### IMDQ-Loaded, OVA-Decorated Nanogels Enable Co-delivery and Lead to Activation and Maturation of Immune Cells through TLR7/8-Dependent Signaling

**Figure 2:**
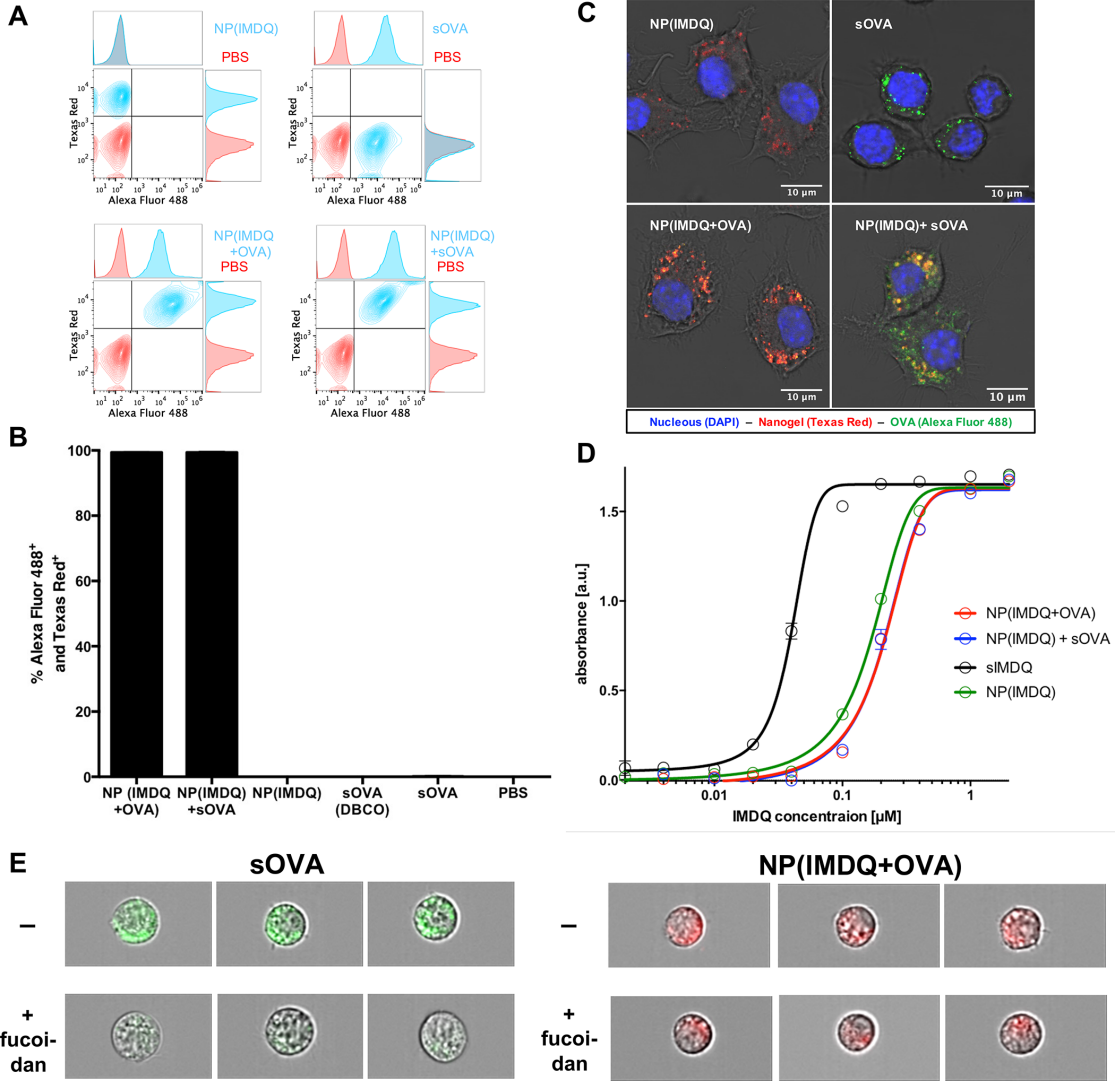
Nanogel-mediated co-delivery and immune cell stimulation by TLR7/8-agonist IMDQ and model protein antigen OVA in an immune reporter cell line. (A) Flow cytometric plots, (B) corresponding percentage of particle and antigen positive RAW-Blue macrophages, and (C) fluorescent confocal microscopy images after incubation with the respective nanogel conjugates and single compounds. (D) TLR agonistic activity measured by NF-kB activation *via* the RAW-Blue reporter assay. (E) Fluorescence imaging of DCs that have internalized soluble OVA (green) (left) or NP(IMDQ+OVA) nanogels. In the presence of the scavenger receptor inhibitor fucoidan (300 μg/mL) the uptake of sOVA is inhibited, but the nanogel sample is still internalized (for quantification, compare Figures S45 and S46).

After successful conjugation of IMDQ and OVA to the pH-responsive nanogels, we wanted to evaluate the system’s co-delivering capacity and initiation of immune responses by its interaction with immune cells. While it has already been demonstrated that IMDQ-loaded nanogels outperform IMDQ-loaded polymer chains regarding TLR activation *in vivo*,^45^ we first sought to investigate the influence of OVA co-administration, either covalently attached to the nanogel surface or solely admixed, on a TLR reporter cell line (Figure 2). Engineered RAW-Blue macrophages allow for a broad screening of TLR activity and were, therefore, selected. *Via* MTT assay, no influence on the cells’ viability was found in the relevant concentration range (Figure S29). By flow cytometry and fluorescent confocal microscopy experiments, RAW macrophages could be characterized to concomitantly internalize nanogels and co-delivered OVA. For that purpose, cells were incubated for 16 h with nanogel and OVA samples. We found that both nanogels and OVA were taken up independently from each other (Figure 2A and Figure S31). Interestingly, both co-delivery of covalently attached or co-administered soluble sOVA provided almost 100% Alexa Fluor 488 and Texas Red positive cells (Figure 2B). Additionally, by confocal microscopy both nanogel and OVA-derived fluorescence could be found internalized by cells (Figure 2C and Figure S32). While for the mixture of NP(IMDQ) and sOVA separate and co-localized fluorescent signals could be found for each species inside the cells, only the NP(IMDQ+OVA) sample with covalently attached OVA provided co-localization of both components inside same compartments. This could also be further confirmed on other antigen-presenting cell lines, *e.g.*, on DC2.4 (compare Figure S33). By flow cytometric analysis applying the ImageStream technology, overlays of OVA-derived fluorescence with bright field imaging confirmed again an intracellular localization of the antigen both in its soluble or particle-bound version (Figure S44). We additionally compared the uptake of sOVA (Alexa Fluor 488-labeled) with the NP(IMDQ+OVA) nanogel conjugate (Cy5-labeled) in the presence of fucoidan, a potent scavenger receptor inhibitor.^56^ In Figure 2E (as well as in Figure S45) scavenger receptor inhibition caused a reduction of internalization for the sOVA, while for the nanogel sample NP(IMDQ+OVA) particles could still get internalized independent from the scavenger receptor (Figure S46). Note that scavenger receptors are usually associated with phagocytosis of bacterial pathogens and other extracellular antigens.^57^ They further afford an intracellular processing leading to more MHC-II antigen presentation than MHC-I presentation. This would favor CD4^+^ T cell responses at the expense of cytotoxic CD8^+^ T cell immune responses.^58^ Circumventing this intracellular processing by using the nanogel-bound version seems to be more attractive in order to enhance cross-presentation and generate a higher amount of antigen-specific CD8^+^ T cells (see Figure 4). These observations underline again that the dual functionalized carriers guarantee successful co-delivery of both antigen and adjuvant into the same immune cell simultaneously.

Moreover, the co-delivered IMDQ is able to stimulate TLR receptor for an effective maturation of the APCs that are required for the induction of cellular and humoral immune responses. This could be monitored by RAW-Blue macrophages *via* secretion of the embryonic alkaline phosphatase (SEAP) in the cell culture supernatant caused by the NF-kB signaling. Compared to non-conjugated IMDQ, nanoparticular conjugated agonist led to a slightly reduced but still highly potent TLR activation in the sub-micromolar regime (Figure 2D). Similarly, OVA-decorated IMDQ nanogels also induced significant TLR activation comparable to IMDQ-loaded nanogels without OVA loading. Hence, OVA loading did not interfere with the TLR agonist–receptor interaction.

**Figure 3:**
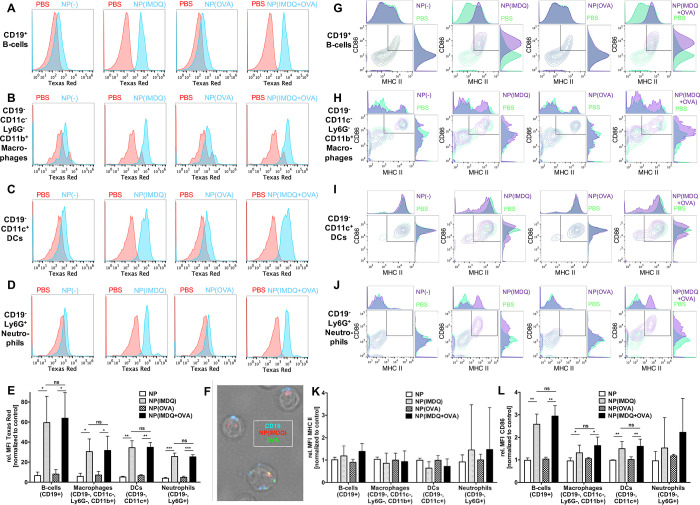
Nanogel-mediated co-delivery and immune cell stimulation by TLR7/8-agonist IMDQ and model protein antigen OVA in primary heterogeneous immune cells. Flow cytometric histograms of particle uptake in B cells (A), macrophages (B), DCs (C), and neutrophils (D) obtained from incubating spleen cells *ex vivo* with the respective nanogel conjugates. (E) Summary of particle-derived relative mean fluorescence intensity (MFI, normalized to PBS as control) of respective immune cells. (F) Fluorescent microscopy image of spleen cells identified as B cells (by CD19 expression, blue) which have internalized both NP(IMDQ) (red) and OVA (green) after incubation of total spleen cells *ex vivo* with NP(IMDQ+OVA). Flow cytometric plots of maturation status by CD86 and MHC-II expression of B cells (G), macrophages (H), DCs (I), and neutrophils (J) after incubating spleen cells with the respective nanogel conjugates. Summary of (K) MHC-II- and (L) CD86-derived relative mean fluorescence intensity (MFI, normalized to PBS as control). Due to immune cell preparation, CD86 serves as appropriate maturation marker for the respective immune cells after incubation with respective nanogel conjugates.

To assess the interaction of the nanogels with immune cells that are more relevant for successful *in vivo* immunization, we next performed similar studies using primary APCs *in vitro*. For that purpose, we selected murine splenocytes as heterogeneous primary immune cell population and incubated them with our nanogel samples (Figure 3), followed by flow cytometric analysis to identify each immune cell population (Figure S34). As visualized by the histogram plots in Figure 3A–D, a boosted nanogel uptake could generally be found when they were loaded with IMDQ in comparison to “empty” nanogels, independently from their additional OVA payload. A more in-depth evaluation of the involved immune cell subsets revealed that this is especially the case for B cells (CD19^+^), but also macrophages (CD19^–^, CD11c^–^, CD11b^+^, Ly6G^–^), DCs (CD11c^+^), and neutrophils (Ly6G^+^) seem to follow this trend (Figure 3E). Interestingly, a pre-incubation of the nanogel samples with mouse serum prior to addition to spleen cells did not affect this uptake behavior (Figure S35). Besides, also by fluorescent microscopy a preferential interaction with B cells could be observed, as both Texas Red-derived nanogel fluorescence and Alexa Fluor 488-derived OVA fluorescence were generally associated with CD19^+^ B cells (Figure 3F and Figure S38). In fact, similar preferential association to B cells has also been found for IMDQ-loaded nanogels *in vivo* after s.c. injection and subsequent analysis of the draining lymph nodes before.^30^

We then also looked at maturation markers in the corresponding immune cell subpopulations by staining them for the expression of the co-stimulatory molecules CD86 and MHC-II, typical maturation markers for successful antigen presentation (Figure 3G–J). Particle-delivered IMDQ triggered the expression levels especially of CD86 in almost all analyzed APCs. MHC-II expression alone was already at relatively high basal levels in the negative control samples due to the applied splenic isolation conditions (Figure S37) and, thus, showed only a minor trend toward IMDQ-mediated maturation (Figure 3K). However, CD86 expression is much more sensitive and was exclusively stimulated by the nanogel-bound IMDQ in almost all immune cell populations (Figure 3L and Figure S36). Interestingly, B cells increased again most strikingly their maturation status. Taken into account their vigorous uptake of IMDQ- and OVA-loaded nanogels, these observations make them promising candidates for successful antigen presenting after i.v. applications, as nanogel pre-incubation with serum did also not alter this behavior either (Figures S36 and S37).

### Two-Component Nanogel Platform Favors CD8^+^ over CD4^+^ T Cell Proliferation *In Vitro*

**Figure 4:**
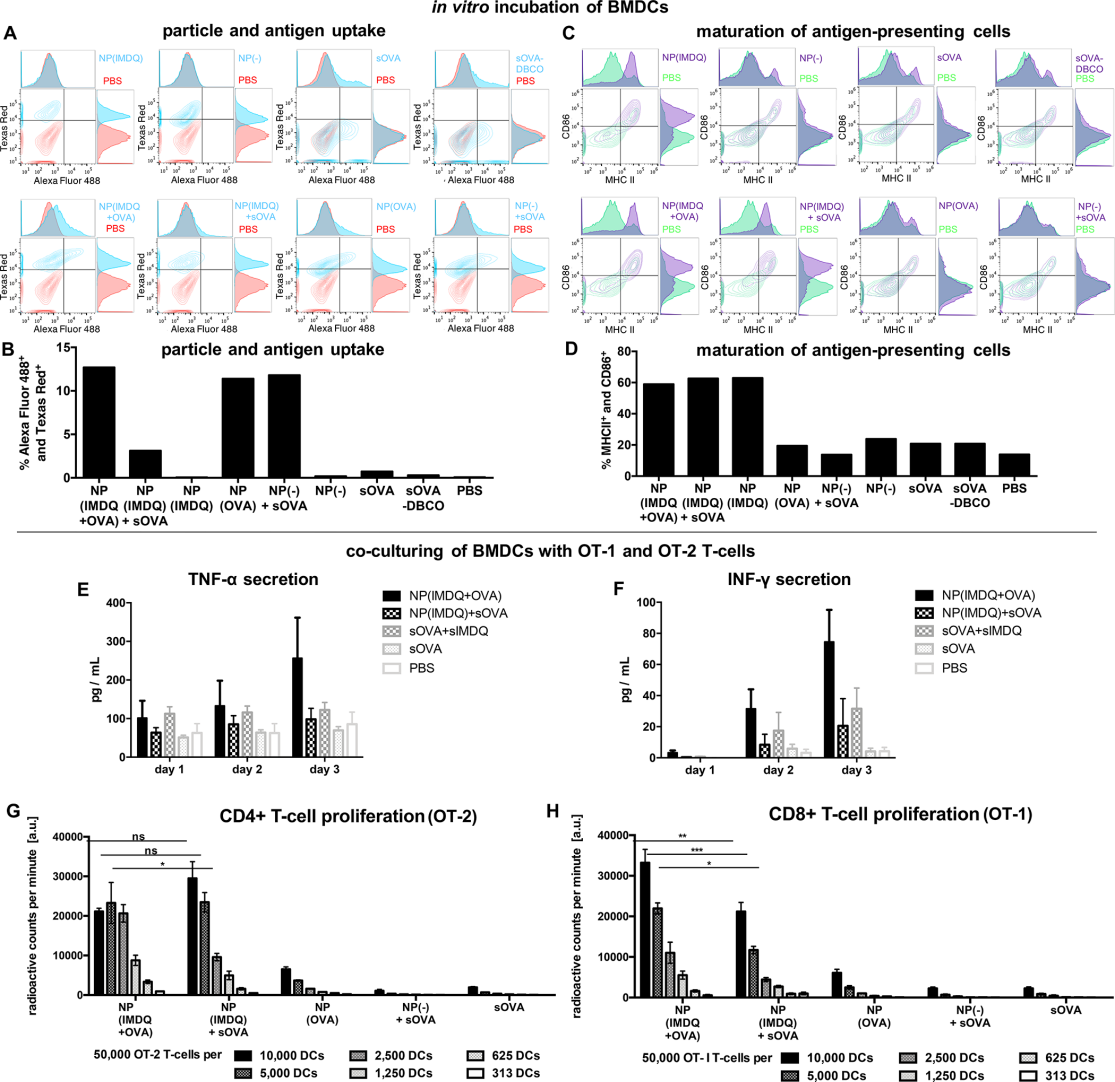
Nanogel-mediated co-delivery of antigen and adjuvant triggers maturation of primary dendritic cells and T cell proliferation *in vitro*. (A) Flow cytometric histogram and (B) percentage of both particle and antigen positive BMDCs after incubation with the respective nanogel conjugates and single compounds. (C) Flow cytometric histogram and (D) percentage of CD86 and MHC-II positive BMDCs after incubation with the respective nanogel conjugates. After co-culturing of nanogel-pulsed BMDCs with OVA-specific T cells secreted cytokines tumor necrosis factor TNF-α (E) and interferon INF-γ (F) were determined from the cell culture supernatant. OVA-specific T cell proliferation was determined at different ratio BMDC to T cell ratios with (G) OT-2 T cells for CD4^+^ T cell proliferation and (H) OT-1 T cells for CD8^+^ T cell proliferation. For the latter, covalently attached OVA seems to favor the secretion of pro-inflammatory cytokines and the induction of a CD8^+^ T cell immune response pathway.

To further elucidate the downstream immune responses triggered by co-delivering antigen and adjuvant through our pH-degradable nanogel platform *in vitro*, we selected bone marrow-derived primary dendritic cells (BMDCs) and incubated them again for 16 h with the nanogel system (Figure 4). The previously observed simultaneous uptake of IMDQ-nanogels and OVA could again be confirmed. Interestingly, flow cytometry analysis revealed that for nanoparticle conjugated IMDQ the percentage of double positive cells (both particle/Texas Red and antigen/Alexa Fluor 488) was increased when OVA was covalently attached to the nanogels’ surface. The amount of internalized sOVA is reduced when cells were already stimulated by NP(IMDQ) (Figure 4A,B and Figure S40). This is in agreement with other studies where maturation of APCs has been shown to reduce additional particle uptake capacities^59^ and, thus, underlines the necessity of antigen and adjuvant co-delivery.

The cells’ maturation profile could again be identified during flow cytometry analysis (Figure S41; for BMDCs, MHC-II expression is now also more reliable). Only for BMDCs pulsed with IMDQ-containing samples, both MHC-II and CD86 maturation markers were upregulated concurrently, while nanogels without TLR7/8 agonist did not trigger BMDC maturation independent of co-administration of OVA, demonstrating again that the nanogel system itself is immunogenically silent (Figure 4C,D).

Moreover, the BMDC maturation approach allowed us to further characterize *in vitro* antigen-specific CD4^+^ and CD8^+^ T cell immune responses, an assumed pre-requisite for successful induction of tumor immunity. Therefore, after stimulation with our IMDQ- and OVA-loaded nanogels overnight, BMDCs were then co-cultivated with dilutions of OVA-specific T cell receptor transgenic OT-1 and OT-2 T cells, isolated from the spleen of OT-1 or OT-2 mice, corresponding to the induction of OVA-specific CD4^+^ (OT-2) and CD8^+^ (OT-1) T cells. On the following days, cell culture supernatants were quantified for pro-inflammatory cytokines that support Th1-biased immune responses, and interestingly the nanogel conjugate of NP(IMDQ+OVA) yielded highest secretion of tumor necrosis factor TNF-α and interferon INF-γ (Figure 4E,F). The T cell proliferation triggered by BMDC maturation and corresponding antigen presentation (either the CD4^+^ epitope for OT-2 or the CD8^+^ epitope for OT-1) could be measured radioactively *via* incorporation of ^3^H-thymidine (Figures S42 and S43). Interestingly, the enhanced co-delivery and stimulation of our two-component nanogel vaccine had again a significant impact on the co-applied T cells. Only the combination of OVA with IMDQ-containing samples led to an increased proliferation of OT-1 and OT-2 cells, emphasizing the importance of co-administering antigen and adjuvant (Figure 4G,H).

Furthermore, covalent attachment of OVA to IMDQ-nanogels led to a significantly increased proliferation of OVA-specific OT-1/CD8^+^ T cells compared to the mixture of sOVA and NP(IMDQ) (Figure 4H). For OT-2 cells (CD4^+^ T cells), however, similar elevated proliferation levels were found for the covalent two-component system as well as the non-covalent mixture (Figure 4G). Consequently, our covalent NP(IMDQ+OVA) construct seems to boost OT-1/CD8^+^ T cell proliferation significantly, probably by the increase in TNF-α and INF-γ secretion. This is in accordance with several previous reports demonstrating that a carrier-mediated co-delivery of OVA favors cross-presentation and the induction of CD8^+^-governed immune responses.^60−62^ Hence, under the applied *in vitro* settings our covalent nanogel platform which co-delivers antigen and adjuvant seems to amend the immune response in favor of CD8^+^ T cell generation.

### Two-Component Nanogel Platform Can Be Safely Applied Intravenously and Generates OVA-Specific Humoral and Cellular Immune Responses

Based on these promising *in vitro* results, we pursued *in vivo* applications for our nanovaccine. It is known that small-molecule TLR agonists are prone to cause systemic immune responses followed by systemic inflammation leading to severe side effects.^27^ Conjugation of TLR7/8 agonists to polymeric carriers, however, has been shown to confine the subsequent immune response to draining lymph nodes after s.c. injection.^28,30^ Interestingly, latest findings by Lynn and co-workers indicate that i.v. injection improves vaccine efficacy compared to s.c. immunization. Especially in case of a physical linkage between antigen and adjuvant affording nanosized particles, CD8^+^ T cell responses were significantly higher after i.v. administration.^34^ However, when applying carrier-bound TLR7/8 agonists into the bloodstream, their influence on hematologic toxicities should be monitored carefully in reflection to other reported systemic type I IFN responses.^63^

In this context, we injected our IMDQ-loaded nanogels i.v. into mice and first analyzed their biodistribution. For that purpose, we used Cy5-labeled nanogels and injected them with and without covalently attached ovalbumin (NP(IMDQ+OVA) and NP(IMDQ)+sOVA) into the tail vein of mice (for each sample an adjuvant dose corresponding to 10 μg of IMDQ was used according to our previous local immunization experiments^30,45^—additionally, an antigen dose corresponding to 30 μg of OVA was used, as at this ratio all antigen can quantitatively be conjugated to the nanogel, compare Figure S23). A mixture of soluble IMDQ and OVA (sIMDQ+sOVA) served as control. Cy5-labeling allowed us to monitor particle organ distribution after dissection using an IVIS imaging system (Figure S48). The majority of the particles were either found in the liver, a typical non-specific sink for systemically administered nanocarriers, and in the kidneys, probably due to the pH-induced particle degradation and unfolding into single polymer chains that can be cleared renally. Nonetheless, a significant amount could still be detected in the spleen as relevant lymphatic organ to induce antigen-specific immune responses.

We therefore conducted flow cytometry analyses of liver and spleen-derived single cell suspensions (Figures S54–S57) and observed an uptake of particles with and without OVA into various antigen-presenting immune cell subpopulations. Interestingly, co-delivery of IMDQ-functionalized particle (Cy5-labeled) with antigen (Alexa Fluor 488-labeled OVA) worked best for the covalent conjugate in most of these immune cells (Figures S55B and S57B). In analogy to the previous *ex vivo* incubation experiments on isolated spleen cells, we also analyzed maturation markers in the corresponding immune cell subpopulations by staining them for co-stimulatory factors CD86 and MHC-II. Interestingly, none of these markers could be stimulated inside the liver, in line with its immunosuppressive microenvironment (Figure S55C,D). However, in the spleen, all IMDQ-containing NP increased the expression levels of CD86 in B cells, DCs, neutrophils ,and macrophages (MHC-II expression alone was again already at relatively high basal levels in the PBS control due to the applied splenic isolation conditions, Figure S57C,D). However, CD86 expression seems again to be more sensitive, as it was also exclusively most stimulated by the nanogel-bound IMDQ in almost all of these immune cell populations in the spleen (Figure S57C).

To further prove that nanogel-bound IMDQ on OVA-loaded nanogels has the potential to induce promising immune responses, we also checked for the cytokine profile of those mice 24 h after i.v. injection and found highest levels of the pro-inflammatory cytokines TNF-α and INF-γ for the dual-loaded nanogel NP(IMDQ+OVA) (Figure S51). These results are in accordance with the cytokine data of the *in vitro*-stimulated BMDCs (compare Figure 4E,F) and confirm that the nanogel-mediated co-delivery of both antigen and immune stimulant provides a good basis for successful immunizations. For additional insights into the innate immune response, we analyzed a larger panel of TLR7/8-driven cytokines also 4 h after the nanogel injection. Again, the nanovaccine led to elevated levels of most analyzed cytokines 24 h after i.v. injection. However, administration of sIMDQ+sOVA resulted in a rapid production of cytokines early after the injection (4 h), illustrating an undesired cytokine storm caused by sIMDQ when applied without a carrier system (Figure S52).

We further aimed to evaluate this toxicity after i.v. injection. Prompted by the strong accumulation of the nanogels in the liver (compare Figures S48 and S55), we looked at the liver enzyme parameters in the blood but could not observe any differences between different samples both after 4 and 24 h (Figures S49 and S50). Moreover, histopathological analyses by hematoxylin–eosin staining of liver, spleen, kidney, heart, and lung tissue showed no histological anomalies after i.v. injection of the nanovaccine (Figure S53).

**Figure 5:**
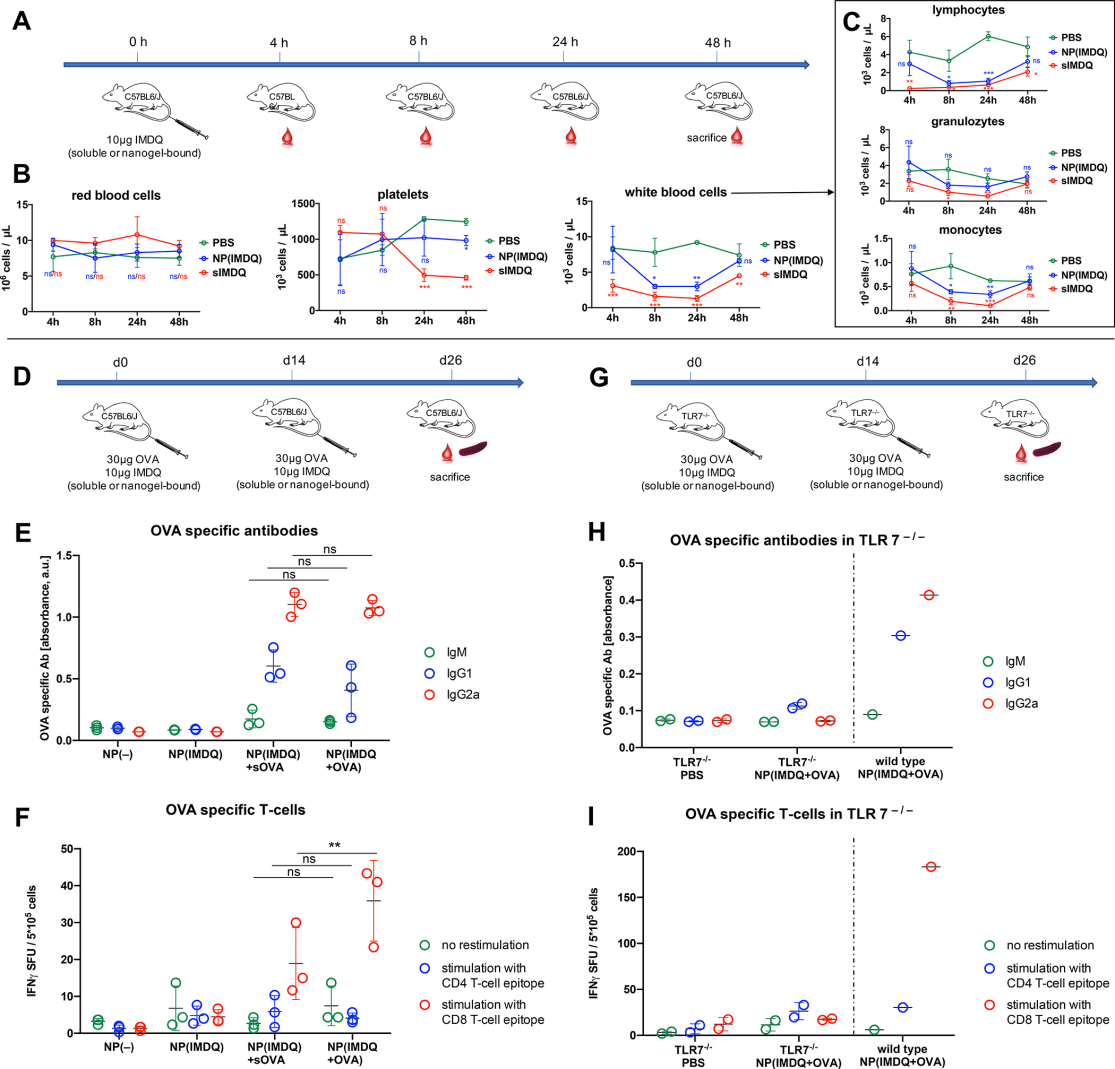
IMDQ- and OVA-loaded nanogel can be administered i.v. safely and generate OVA-specific humoral and superior cellular immune responses. (A) Schedule for analyzing hematologic toxicities of soluble or nanogel-bound TLR7/8 agonist IMDQ after single i.v. injection. (B) Corresponding blood cell results and (C) their differentiated white blood cells. (D) Immunization schedule for IMDQ- and OVA-loaded nanogel in wild-type mice and (E) the corresponding OVA specific antibodies determined by ELISA of the blood serum, as well as (F) the corresponding T cells determined by ELISpot of the isolated spleen cells. (G) Analogous immunization schedule for IMDQ- and OVA-loaded nanogel in TLR7^–/–^ mice. (H) No OVA-specific antibodies could be found by ELISA in the blood serum as well as (I) no corresponding T cells could be determined by ELISpot of the isolated spleen cells, in contrast to a wild-type mouse serving as control during these experiments.

However, a major impact especially for the i.v. administration of the soluble IMDQ could be detected on the composition of cells in the blood (Figure 5A–C). Earlier experiments revealed that, when bound to nanogels, the negative influence on the blood cell profile can be prevented and the immune response remains localized to draining lymph nodes after s.c. injection.^30,49^ To provide an overview of IMDQ’s impact after i.v. injection over time, mice were treated again with 100 μL of samples containing 10 μg equivalents of IMDQ, and blood samples were taken after 4, 8, 24, and 48 h for analysis of their red and white blood cell content as well as for the amount of platelets (Figure 5A). Systemic injection of TLR agonists resulting in uncontrolled release of type I interferons has been linked to strongly reduced numbers of platelets and white blood cells in the blood.^63^

While the number of red blood cells was not affected during all our studies (Figure 5B), the massive drop of platelets could be reconfirmed for the sIMDQ after 24 h, while the nanogel-bound TLR7/8 agonist had no effect, also after 48 h (Figure 5B). We relate this unfavorable site effect of the sIMDQ to the earlier detected massive cytokine expression when applied without a carrier system (Figure S52). Interestingly, such immunization-related severe thrombocytopenia is also associated as side effect of currently administered viral vector-based SARS-CoV-2 vaccines causing sinus vein thrombosis^64^ and should therefore be circumvented already at an early stage of vaccine development.

Compared to sIMDQ, mice treated with nanogel-bound IMDQ (NP(IMDQ)) displaced no drop in platelets and only a reduced drop in lymphocytes that recovered more after 48 h effectively (Figure 5B). Specifically, white blood cell analyses revealed that the levels of lymphocytes, granulocytes and monocytes were also less affected by the nanogel-bound IMDQ than by sIMDQ and recovered to values of PBS-treated mice (Figure 5C). Moreover, empty nanogels or IMDQ-loaded nanogels decorated with OVA antigen never affected all hematologic parameters as much as sIMDQ after i.v. administration (Figure S47). Altogether, these findings highlight the enhanced safety profile of nanogel-bound IMDQ even after injection into the bloodstream, avoiding adverse systemic immune responses but instead providing more access to circulating immune cells for improved vaccination performance.

Proven effective and safe for i.v. injection, we used our two-component nanogels for immunizing naïve wild-type mice. On day 0 they were immunized i.v. with different nanogel samples, namely empty nanogels NP(−), IMDQ-loaded nanogels NP(IMDQ), two-component nanogels NP(IMDQ+OVA), and IMDQ nanogels mixed with sOVA (NP(IMDQ)+sOVA), followed by a boost immunization on day 14 (Figure 5D). On day 26 mice were sacrificed to analyze the generation of OVA-specific antibodies in the blood serum by ELISA (Figure 5E) and OVA-specific T cells in the spleen by ELISpot analysis (Figure 5F). As expected, only mice immunized with samples containing OVA and IMDQ showed secretion of OVA-specific antibodies. Both formulations, two-component vaccine and soluble mixture, led to increased secretion of IgG-type antibodies, especially the IgG2a subtype, which indicates a Th1-biased immune response. Here, covalent attachment of OVA did not have any drastic influence on the humoral response (Figure 5E). On the cellular level, however, spleen analysis of mice immunized with NP(IMDQ+OVA) revealed a significantly increased number of OVA-specific CD8^+^ T cells compared to NP(IMDQ)+sOVA (Figure 5F). This observation is in accordance with our previous *in vitro* results (Figure 4G) indicating that OVA-bound IMDQ-nanogels mediate enhanced cross-presentation and favor a CD8^+^ T cell response also *in vivo*.

Additionally, immunization experiments were repeated in TLR7^–/–^ mice (Figure 5G) and revealed no priming of specific B cell (Figure 5H) and T cell (Figure 5I) immune responses. This clearly demonstrates that the nanogel-mediated Th1-biased humoral and cellular immune responses are mediated by TLR7 receptor stimulation triggered through nanogel-conjugated IMDQ.

Based on these results, we analyzed the effects of i.v. vaccination compared to s.c. vaccination on humoral and cellular responses, as similar systems have already been shown suitable for s.c. injection by us and others.^30,34^ Hence, mice were injected twice with our nanovaccine. Again blood serum and spleen cells were tested for OVA-specific antibodies and T cells. This time, MHC-tetramer staining revealed CD8^+^ T cells in blood after prime and boost immunization with our nanovaccine (Figure S58) but no significant differences between s.c. and i.v. administration. Similarly, recent data from Baharom *et al.* shows that i.v. vaccination does not trigger higher numbers of antigen-specific T cells compared to s.c. vaccination but creates subtypes of CD8^+^ T cells with superior antitumor capacity.^65^ In our study, i.v. administration of NP(IMDQ+OVA) significantly outperformed s.c. injection in the generation of OVA-specific IgG2a antibodies (Figure S59A). Furthermore, investigating the INF-γ secretion of OVA-specific T cells *via* ELISpot, we found an improved performance of the i.v. immunization for both CD4^+^ and CD8^+^ T cells (Figure S59B).

In addition, mice body weight was monitored over time during these immunization studies. Note that NP(IMDQ+OVA) conjugate was well tolerated for i.v. and s.c. injection routes. However, the administration of sIMDQ as adjuvant led to a rapid drop in body weight and confirmed the necessity of covalent IMDQ-attachment once more (Figure S60) and its impact on generation of OVA-specific T cells (Figure S61).

Summarizing, our IMDQ nanogels could be demonstrated as safe for i.v. immunization and elicit robust humoral and cellular immune responses. Furthermore, our results are indicating that covalent attachment of OVA benefits the formation of OVA-specific cytotoxic CD8^+^ T cells both *in vitro* and *in vivo*.

### Prophylactic Immunization with Two-Component Nanovaccine Reduces Tumor Growth and Leads to Enhanced Tumor Protection during Therapeutic Vaccination

**Figure 6:**
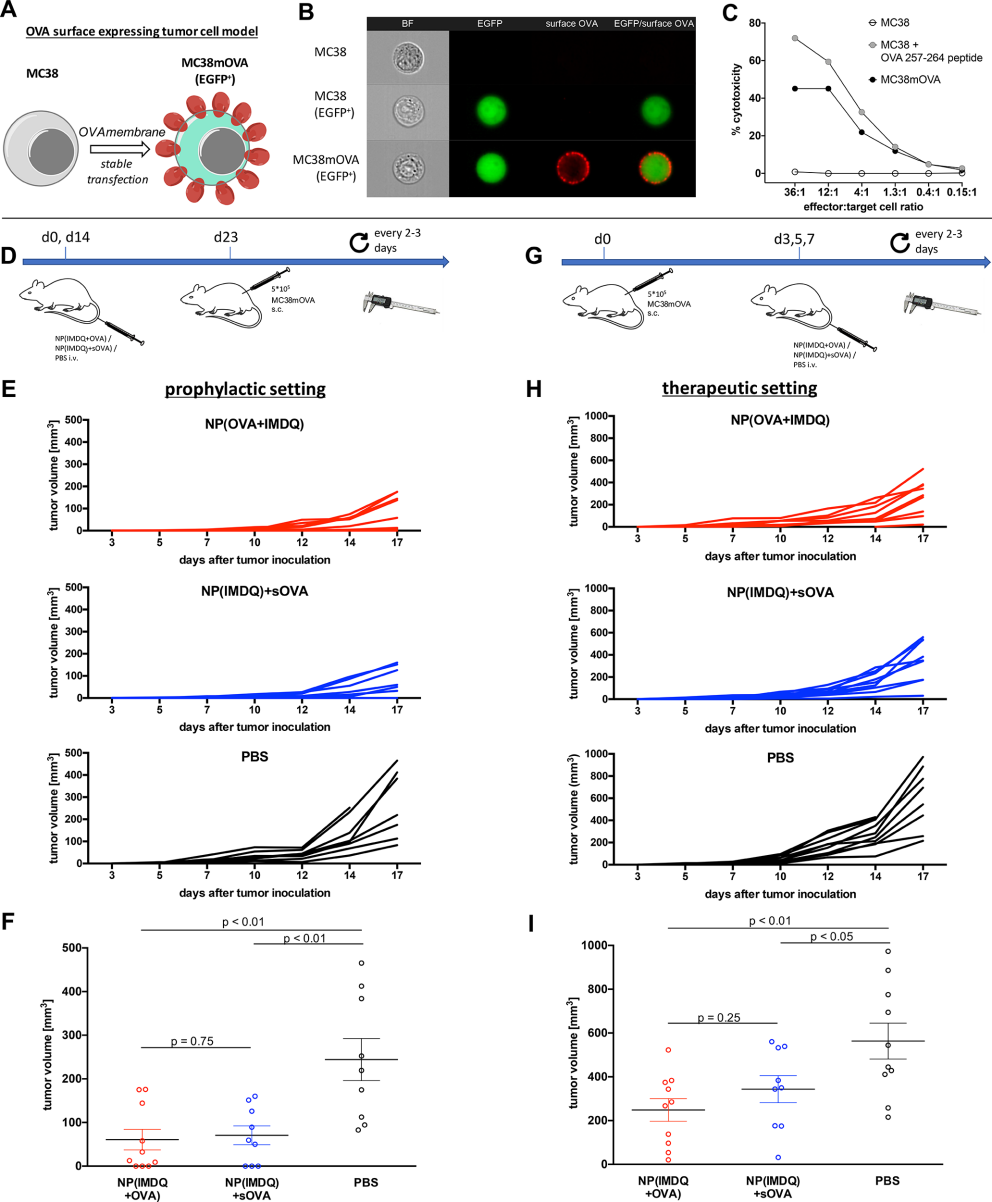
IMDQ- and OVA-loaded nanogels provide both prophylactic and therapeutic immunity toward a surface neoantigen-expressing tumor model. (A) MC38 cancer cells were genetically engineered to stably express OVA on their surface. (B) ImageStream analysis of wild-type MC38 cells (top), MC38 cells expressing only EGFP (middle) and MC38mOVA (EGFP^+^, bottom) stained for surface OVA by OVA-specific antibodies. Image panels (left to right) show brightfield (BF, magnification 40×), EGFP expression (green), surface OVA expression (Alexa Fluor 647, red), and overlay image (EGFP/surface OVA). (C) CD8^+^ T cell-mediated killing of MC38mOVA and control target cells (MC38 and peptide-pulsed MC38 (1 μM, 45 min at 37 °C)) after incubation with OT-I T cells at the indicated ratios (specific target lysis was calculated as described in the Supporting Information). (D) Prophylactic immunization schedule and challenge with MC38mOVA tumor cells. (E) Results of the individual tumors after prophylactic immunization with the corresponding nanogel samples or PBS (*n* = 10). (F) End point tumor volume showing reduced tumor growth for NP(IMDQ+OVA)- and NP(IMDQ)+sOVA-immunized mice compared to PBS group; however, no significant difference between NP(IMDQ+OVA) and NP(IMDQ)+sOVA could be found. (G) Therapeutic schedule for the treatment of mice challenged with MC38mOVA tumor cells (*n* = 10). (H) Results of the individual tumors after therapeutic treatment with the corresponding nanogel samples or PBS. (I) End point tumor volume showing reduced tumor growth for NP(IMDQ+OVA) and NP(IMDQ)+sOVA-treated mice compared to PBS group. A more significant difference between NP(IMDQ+OVA) and NP(IMDQ)+sOVA could be found compared to the prophylactic treatment.

The presence and the activation of antigen-specific cytotoxic T cells in the tumor microenvironment is often correlated with improved tumor regression and therapy output.^66^ Referring to superior generation of OVA-specific CD8^+^ T cells after immunization with our two-component nanogels, we next asked whether this observation might be displayed by enhanced tumor regression *in vivo*, too. For that purpose, we first aimed to implement an OVA-dependent tumor model system that is accessible to both humoral and cellular immune responses (the classical B16-OVA model, for instance, has some limitations: its cytosolic OVA expression does not provide access for antibody-mediated immune responses^67^ and its downregulated MHC-I expression allows only reduced CD8 epitope presentation^68^). We therefore first selected the MC38 colon cancer cell line and established an OVA antigen model for our studies (Figure 6).

Cancer immunotherapy seeks to enable immunization against tumor-specific antigens either as overexpressing antigens or as neoantigens. In contrast to patient-specific neoepitopes usually found intracellularly, we hypothesize that cancer-specific cell surface expressed neoantigens display better suited targets since they are accessible for both cellular as well as humoral immune responses. Hence, the MC38 tumor model was genetically engineered to stably express membrane-bound OVA and, thereby, allows the elimination by OVA-specific cellular as well as humoral immune responses (Figure 6A). OVA surface expression could be verified by ImageStream analysis, clearly showing OVA-dependent fluorescence at the cell surface after incubation with OVA-specific antibodies (non-transfected or EGFP transfected control cells could not be stained by OVA-specific antibodies on their surface) (Figure 6B). These data suggest that these tumor cells can be detected by humoral immune responses. Furthermore, OVA-expressing MC38 cells were also recognized and eliminated by OVA-specific CD8^+^ T cells obtained from OT-1 mice. When co-incubated for 4 h with OT-1 T cells, OVA-surface-expressing MC38 cells (MC38mOVA) were killed in a T cell dose-dependent way, in analogy to wild-type MC38 cells externally loaded with MHC-I binding CD8 T cell epitope OVA 257-264 peptide. Non-treated MC38 cells lacking OVA expression were not lysed at all (Figure 6C). Consequently, these experiments confirm that also cellular immune responses are capable of recognizing and responding to our tumor model in an antigen-specific fashion. Summarizing, our OVA-expressing MC38 tumor cell model was considered to be suitable for analyzing tumor-specific humoral and cellular immune responses triggered by our two-component nanovaccine.

Subsequently, the OVA membrane-expressing MC38 cell line was applied to wild-type mice. We first assessed prophylactic tumor protection *in vivo* by immunization two times (on day 0 and day 14) with either NP(IMDQ+OVA) conjugate or the mixture NP(IMDQ)+sOVA (Figure 6D). On day 23 mice were then subcutaneously inoculated with membranous-expressing OVA MC38 cells and tumor growth was analyzed each 2–3 days. While tumors rapidly grew in the non-immunized control group (PBS) from day 12 after inoculation, prophylactic immunization against OVA with both nanogel samples resulted in significantly reduced tumor growth (Figure 6E). However, no significant differences in tumor protection based on the nature of OVA delivery, covalently attached or administered, could be observed by comparing final tumor volumes (Figure 6F). In accordance with our previous *in vivo* findings that both NP(IMDQ+OVA) conjugate or the mixture NP(IMDQ)+sOVA were able to induce similar humoral immune responses (Figure 5E), we hypothesize that these mechanisms might primarily be responsible here to inhibit growth of the surface antigen-expressing tumor cells under prophylactic conditions.

Alternatively, we further assessed the influence of NP(IMDQ+OVA) on tumor regression under therapeutic conditions. For that purpose, mice were first inoculated s.c. with OVA MC38 cells on day 0, and on day 3 palpable tumors could be detected at a volume below 5 mm^3^. Next, i.v. immunization with NP(IMDQ+OVA) or NP(IMDQ)+sOVA was performed on days 3, 5, and 7 (Figure 6G). Again, analysis of tumor growth showed that both formulations could trigger significant tumor regression (Figure 6H). Interestingly, treatment with NP(IMDQ+OVA) seemed to have a slightly improved effect on controlling final tumor volumes than the mixture of NP(IMDQ)+sOVA (Figure 6I). This is in line with our previous finding that covalent attachment of OVA guarantees more efficient co-delivery of OVA and IMDQ followed by rapid immune cell stimulation, cross-presentation, and induction of OVA-dependent CD8^+^ cells (Figure 5F).

### OVA- and IMDQ-Loaded Two-Component Nanovaccine Governs Antigen-Specific Tumor Growth by Increasing the Number of Infiltrating Immune Cells into the Tumor Microenvironment

**Figure 7:**
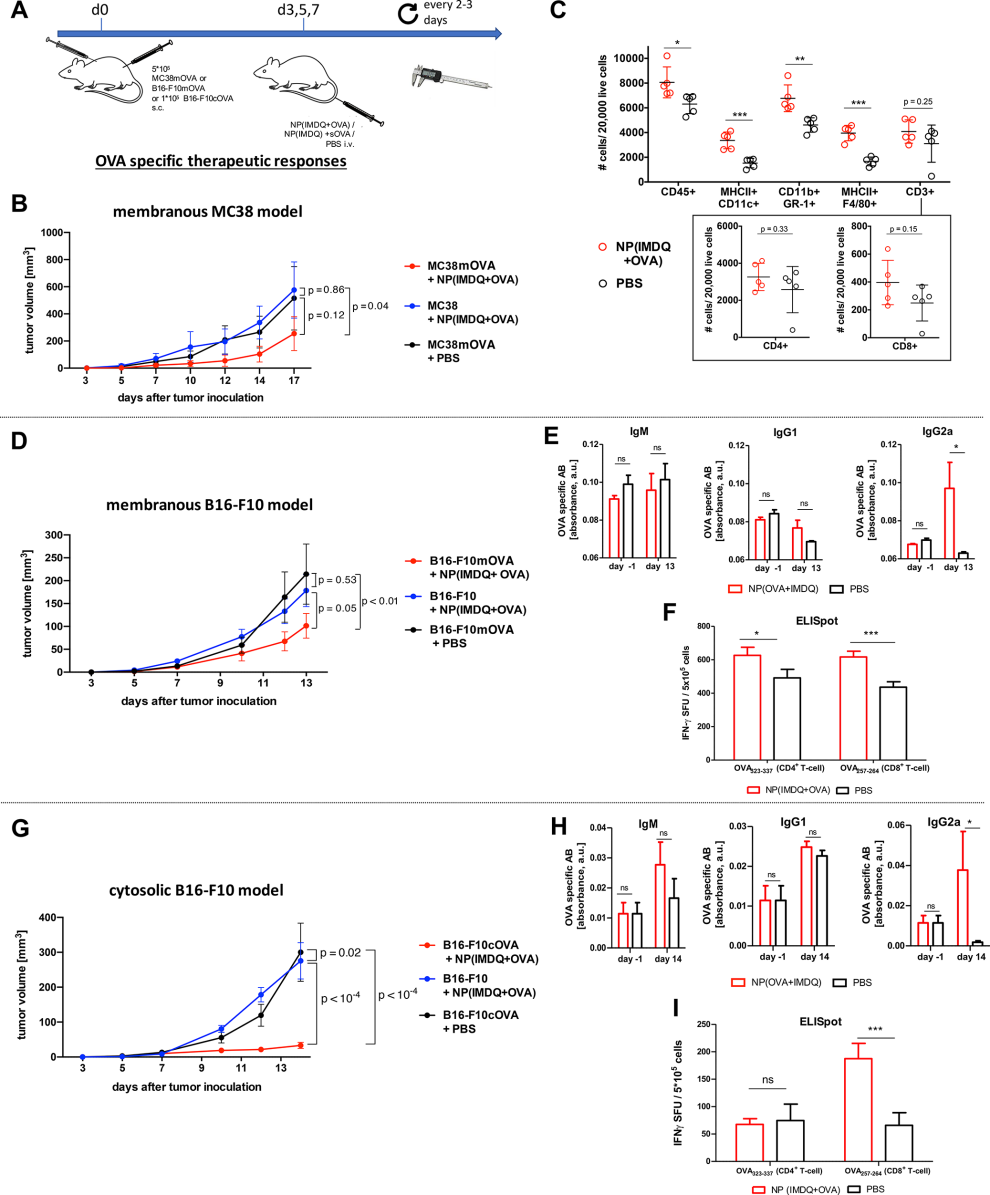
IMDQ- and OVA-loaded nanogels provide antigen-specific tumor immunity by induction of Th-1-biased immune responses with respect to increasing levels of antigen-specific IgG2a titers, increasing numbers of antigen-specific CD8^+^ and CD4^+^ T cells and increasing numbers of tumor-infiltrating immune cells. (A) Therapeutic schedule for the treatment of mice challenged with both MC38mOVA and wild-type MC38, B16-F10mOVA and wild-type B16-F10, or B16-F10cOVA and wild-type B16-F10 tumor cells. (B) Results of the MC38mOVA or MC38 tumor sizes after treatment either with NP(IMDQ+OVA) or PBS (*n* = 10). (C) Flow cytometric analysis of single-cell tumor suspensions derived from MC38mOVA tumors treated with NP(IMDQ+OVA) or PBS. (D) Results of B16-F10mOVA or B16-F10 tumor sizes after treatment with NP(IMDQ+OVA) or PBS (*n* = 9–11). (E) ELISA analysis of blood serum samples taken on day −1 (so before tumor inoculation and treatment with the nanogel) and day 13 after tumor inoculation from B16-F10mOVA-bearing mice treated with NP(IMDQ+OVA) or PBS. (F) ELISpot analysis of the isolated spleen cells taken on day 13 after tumor inoculation from B16-F10mOVA-bearing mice treated with NP(IMDQ+OVA) or PBS. (G) Results of B16-F10cOVA or B16-F10 tumor sizes after treatment with NP(IMDQ+OVA) or PBS (*n* = 10). (H) ELISA analysis of blood serum samples taken on day −1 and day 14 after tumor inoculation from B16-F10cOVA-bearing mice treated with NP(IMDQ+OVA) or PBS. (I) ELISpot analysis of the isolated spleen cells taken on day 14 after tumor inoculation from B16-F10cOVA-bearing mice treated with NP(IMDQ+OVA) or PBS.

In a subsequent experiment, we wanted to confirm these observations and investigated whether the observed antitumor effects fully rely on OVA-specific immune responses. For that purpose, mice were inoculated on one flank with MC38mOVA cells and on the other flank with wild-type MC38 cells not expressing OVA. On days 3, 5, and 7, mice were then treated with NP(IMDQ+OVA) or PBS as control (Figure 7A). Whereas nanovaccine treatment induced again an immune response that recognized the OVA-expressing MC38 tumors selectively and caused reduced tumor growth, the MC38 tumors did not respond to the immunization and grew comparable to the MC38mOVA tumors of PBS-treated mice (Figure 7B).

Moreover, we looked at the immune status by determining the number of infiltrating immune cells in the MC38mOVA tumor micromilieu after treatment with NP(IMDQ+OVA) (Figure 7C and Figures S63 and S64). We generally observed a significant increase in the number of immune cells (CD45^+^) compared to PBS-treated tumors, most notably in number of infiltrating myeloid cell populations, as reflected by an increase in numbers of MHC-II^+^ CD11c^+^ dendritic cells, CD11b^+^ Gr-1^+^ neutrophils, and MHC-II+F4/80^+^ macrophages, all considered as antitumoral. Beyond that, also a slight increase in CD3^+^ T cells was observed. When analyzing this population more carefully, we confirmed that the number of infiltrating CD8^+^ T cells, but not of CD4^+^ T cells, increased within the tumors after treatment with NP(IMDQ+OVA), reflecting the proposed enhanced cellular antitumoral immune response induced by NP(IMDQ+OVA).

Based on these results, we further applied the same experimental conditions using the commonly used B16-F10 tumor model but also engineered the cell line with a membrane expressing OVA in analogy to the MC38 model. Although the B16-F10 cell line is known to have downregulated MHC-I expression and, thus, only reduced CD8 epitope presentation,^68^ we observed again similar results for this B16-F10mOVA model as for the MC38mOVA model. Therapeutic treatment with our two-component vaccine led to an antigen-specific reduction of the transplanted B16-F10mOVA tumor, while the wild-type B16-F10 tumors were not affected in the same way as the untreated B16-F10mOVA tumors (Figure 7D). These results confirm that our IMDQ- and OVA-loaded nanogel fully guarantees selective antigen-specific antitumor responses in mice after i.v. administration, independent from the tumor source.

To shine further light on the immunologic mechanism behind these findings, we performed ELISA analysis of blood serum samples taken from of B16-F10mOVA-bearing mice before tumor inoculation (day −1) and after nanogel treatment (day 13 after tumor inoculation). Again, they revealed a significant increase especially of IgG2a antibodies after treatment with NP(IMDQ+OVA) (Figure 7E), indicating a Th1-biased immune response in analogy to the previous immunization experiments (Figure 5D). This was further proven by cytokine analysis of those blood samples revealing a significant induction of the pro-inflammatory cytokines TNF-α and INF-γ (Figure S62), as already demonstrated after nanogel treatment *in vitro* (Figure 4E,F) and *in vivo* (Figure S51). This finally resulted in an increase of the number of OVA-specific CD4^+^ and CD8^+^ T cells in the spleen determined by ELISpot analysis (Figure 7F), which are again in accordance with our immunization studies (Figure 5F).

Indicating that the antigen-specific generation of antibodies plays a crucial role in the rejection of tumors that present OVA on the cell surface, we decided to investigate the impact of humoral and cellular immune responses in a mice model lacking antibody production. Hence, antibody-deficient mice, referred to as IgMi mice,^69^ were challenged with MC38mOVA tumors but in contrast to wild-type mice no tumor reduction was observable. Further analysis of spleen cells revealed that IgMi mice, besides lacking antibodies, also show reduced generation of antigen-specific CD8^+^ T cells compared to wild-type mice (Figure S65). Hence, we propose that failed tumor rejection in IgMi mice cannot exclusively be attributed to antibody deficiency and might also depend on reduced generation of T cells. This makes it difficult to draw clear conclusions regarding the impact of antibodies and T cells on tumor control.

Therefore, we modified our tumor model by generating B16-F10 cells expressing OVA exclusively in the cytosol (B16-F10cOVA). Interestingly, we were also able to observe antigen-specific and robust reduction of tumors in this model, while control wild-type B16-F10 tumors were not affected (Figure 7G). Subsequent analysis of antibody secretion and OVA-specific T cell generation revealed again increased secretion of OVA-specific IgG2a antibodies and more importantly a rise in OVA-specific CD8^+^ T cells (Figure 7H,I), giving evidence that the induction of cellular immune responses is primarily essential for the antitumor effect induced by our nanovaccine. These properties might therefore also become relevant when addressing currently investigated clinical settings of cancer-specific neoepitopes.

Altogether these results confirm that the i.v.-administered, co-delivering two-component nanovaccine NP(IMDQ+OVA) induces robust antigen-specific humoral and cellular immune responses *in vitro* and *in vivo* that install enhanced antitumor efficacy, after both prophylactic and therapeutic immunization.

## Conclusion

Therapeutic vaccination against tumor-associated antigens is of great interest regarding the variety of tumor types and individual immune condition for each patient. Vaccines need to elicit antigen-specific stable humoral and cellular immune responses to ensure immunogenicity and avoid tolerance. Here, we reported on a RAFT-based nanogel system that chemically allows covalent attachment of antigens and core functionalization with small molecules in a straightforward way, resulting in a two-component nanovaccine that is safe for i.v. immunization and allows physical co-delivery of antigen and adjuvant. We showed that covalent attachment of OVA to our IMDQ-loaded nanogels elicits robust humoral and cellular immune responses and, in addition, benefits the generation of antigen-specific CD8^+^ T cells both *in vitro* and *in vivo*. These observations are in accordance with studies by other groups that emphasize the importance of incorporation of immune adjuvant and antigen into the same carrier system.^34−36^ The development of well-defined OVA-dependent MC38 and B16-F10 tumors facilitated tumor studies with membrane-bound OVA, mimicking the expression of tumor-associated neoantigens in clinical relevant tumors that are responsive toward both humoral and cellular immune responses as well as studying the exclusive impact of cellular immunity on cytosolically expressed OVA.

Our nanogels elicited OVA-dependent antitumor responses in both prophylactic and therapeutic approaches. The demand for personalized cancer vaccines promotes the need for customizable vaccine platforms. Regarding the chemical design of our nanogel platform, easy modifications toward co-delivery of other immune-interfering drugs and antigens or peptides are possible, since both components only need to exhibit either amino functionalities due to the reactive ester approach for core conjugation or DBCO modification for ligation to the particle surface. As well, multi-targeting by combination with immune checkpoint inhibitors or different antigens on one nanogel is conceivable. Based on the selected RAFT polymerization conditions and core-cross-linking of polymeric micelles, the size and morphology of our nanovaccines are pre-defined before functionalization; hence, the nanoparticulate formulation is fully independent of the immunologically relevant payload. Their pharmacokinetic profile will exclusively rely on the performance of the immunogenically silent carrier. In addition, the results of this study conclude that the pH-degradable nanogel system further facilitates a safe i.v. administration of highly immune stimulating imidazoquinoline-type TLR7/8 adjuvants in combination with co-delivered cancer-associated antigens and, thus, provides—in contrast to currently investigated antigen-free adjuvant treatments of tumors—opportunities to install cancer-specific immunity.

Overall, our nanogel approach can be considered as a highly versatile immunocarrier platform that is able to trigger antitumor capacities and might be interesting for the development of highly customized nanovaccines, not only for clinically more relevant tumors but also against other pandemic viral diseases.

## Materials and Methods

Detailed information on instrumentations, materials, cells and mice, as well as experimental procedures can be found in the Supporting Information.

## Supporting Information Available

Instrumentation, materials, cells and mice, experimental procedures, and data, including Figures S1–S65 (PDF)
