# Supplementary material for: Systemically Administered TLR7/8 Agonist and Antigen-Conjugated Nanogels Govern Immune Responses against Tumors
Source: ACS Nano. 2022 Feb 1;16(3):4426–43. doi: 10.1021/acsnano.1c10709 (PMC8945363; doi:10.1021/acsnano.1c10709)
Supplement: Supplementary file 1 — nn1c10709_si_001.pdf [file nn1c10709_si_001.pdf]

Systemically Administered TLR7/8-Agonist and Antigen Conjugated Nanogels Govern Immune Responses against Tumors

- Supporting Information -

Judith Stickdorn,[1,‡] Lara Stein,[2,‡] Danielle Arnold-Schild,[2] Jennifer Hahlbrock,[2] Carolina Medina-Montano,[3] Joschka Bartneck,[4] Tanja Ziß,[2] Evelyn Montermann,[3] Cinja Kappel,[3] Dominika Hobernik,[3] Maximilian Haist,[3] Hajime Yurugi,[5] Marco Raabe,[1] Andreas Best,[1] Krishnaraj Rajalingam,[5] Markus P. Radsak,[4] Sunil A. David,[6] Kaloian Koynov,[1] Matthias Bros,[3] Stephan Grabbe,[3,*] Hansjörg Schild,[2,*] Lutz Nuhn[1,*]

[‡]: equally contributed.
[1]: Max Planck Institute for Polymer Research, Ackermannweg 10, 55128 Mainz, Germany – email: lutz.nuhn@mpip-mainz.mpg.de.
[2]: Institute of Immunology, University Medical Center of Johannes Gutenberg-University Mainz, Langenbeckstraße 1, 55131 Mainz, Germany – email: schild@uni-mainz.de.
[3]: Department of Dermatology, University Medical Center of Johannes Gutenberg-University Mainz, Langenbeckstraße 1, 55131 Mainz, Germany – email: stephan.grabbe@unimedizin-mainz.de.
[4]: IIIrd Department of Medicine - Hematology, Oncology, Pneumology, University Medical Center of the Johannes Gutenberg-University Mainz, Langenbeckstraße 1, 55131 Mainz, Germany
[5]: Cell Biology Unit, University Medical Center of Johannes Gutenberg-University Mainz, Langenbeckstraße 1, 55131 Mainz, Germany
[6]: ViroVax, LLC, 2029 Becker Drive Suite 100E, Lawrence 66047-1620, Kansas, United States.
* corresponding authors (lutz.nuhn@mpip-mainz.mpg.de, schild@uni-mainz.de, stephan.grabbe@unimedizin-mainz.de).

Instrumentation

All ^{1}H- and ^{19}F-NMR spectra were recorded on a Bruker 400 MHz FT NMR spectrometer. Chemical shifts (δ) are provided in ppm relative to TMS. Samples were prepared in $CDCl_3$ and their signals referenced to residual nondeuterated signals of the solvent. UV-Vis spectra were recorded with a Jasco V-630 spectrophotometer with a JASCO ETC-717 peltier thermostatted cell holder. Spectra were recorded at 20 °C using a water thermostat (A. Knüss Optronic V50). For analytical size exclusion chromatography (SEC) with THF as eluent, columns packed with MZ-Gel SDplus 102 Å and MZ-Gel SDplus 106 Å obtained from MZ-Analysentechnik were used. Polymers were detected at a wavelength of 254 nm with a UV-detector (JASCO UV-1575) and a RI-detector (JASCO RI-1530). Molecular weights were determined with a polystyrene calibration obtained from PSS Polymer Standard Services GmbH and toluene as internal standard. Dynamic light scattering (DLS) measurements were carried out using a ZetaSizer Nano ZS instrument (Malvern Instruments Ltd., Worcestershire, UK) equipped with a He-Ne laser. Measurements were performed at a temperature of 25 °C and a detection angle of 173°. Micro BCA Protein Assay, RAW-Blue TLR reporter assay and MTT assay were performed with a Spark 20M Multimode Microplate Reader from Tecan. SDS-PAGE was performed with a Mini-PROTEAN Tetra Cell from Bio-Rad. Gel imaging was conducted with a smart phone camera.

Fluorescence cross-correlation spectroscopy (FCCS) experiments were performed on a commercial setup (LSM 880, Carl Zeiss, Jena, Germany).

Flow cytometric analyses were done with an Attune NxT flow cytometer (Thermo Fisher).

Materials, Cells and Mice

Unless otherwise stated, solvents and chemicals were obtained from Sigma Aldrich and Acros Organics. Dioxane and THF were dried over sodium, DCM over calcium hydride, and freshly distilled prior to use. Millipore water was prepared using a MILLI-Q® Reference A+ System. DBCO-PEG_4-NHS ester was purchased from Jena Bioscience, Alexa Fluor-TFP ester from Thermo Fisher and EndoFit™ Ovalbumin from InvivoGen. AMDVN (V-70) was obtained from Wako Chemical and the TLR 7/8 agonist 1-(4-(aminomethyl) benzyl)-2-butyl-1H-imidazo[4,5-c]quinolin-4- amine (IMDQ) could be provided following an earlier report.[1] Dialysis was performed using Spectra/Por 3 membranes obtained from Spectrum Labs with a molecular weight cutoff 1000 g mol−1.

Dulbecco's PBS, cell culture medium and supplements were purchased from Thermo Fisher Scientific. The RAW-Blue reporter cell line and QUANTI-Blue were obtained from InvivoGen.

RAW-Blue macrophages purchased from InvivoGen were cultured in DMEM-GlutaMAX™ medium supplemented with 10% fetal bovine serum (FBS), 1% penicillin/streptomycin, 0.01% zeocin and 0.02% normocin at 37°C with 5% CO_2 saturation.

DC2.4 cells, kindly provided by V. Mailänder (Department of Dermatology, University Medical Center, Johannes Gutenberg-University Mainz) were maintained in IMDM (Thermo Fisher Scientific) supplemented with 5 % FBS, 2 mM L-Glutamine, 200 mg/mL penicillin and 200 U/mL streptomycin.

MC38 colon adenocarcinoma cells were kindly provided by H.-C. Probst (Institute for Immunology, University Medical Center, Johannes Gutenberg-University Mainz) and B16-F10 melanoma cells were purchased from ATCC (USA). Stable cell lines expressing membrane-bound OVA (MC38mOVA, B16-F10mOVA) were produced by electroporation using expression plasmids (pIRES2-EGFP (Clontech) for MC38 and pcDNA3.1+P2A-eGFP (Genscript Biotech) for B16-F10) containing cDNA encoding full-length OVA protein linked to the transmembrane region of H2-D^b, which was generously provided by T. Tedder (Department of Immunology, Duke University School of Medicine, North Carolina). To obtain stable B16-F10 cells expressing cytosolic OVA (B16-F10cOVA), B16-F10 cells were electroporated using the pcDNA3.1+P2A-eGFP vector containing cDNA encoding full-length OVA. Cells expressing EGFP were selected by three rounds of screening using a FACS Aria (BD Biosciences) at the Core Facility of the Research Center for Immunotherapy (University Medical Center, Johannes Gutenberg-University Mainz. Cells were maintained in DMEM high glucose supplemented with 10 % FBS, 2 mM L-Glutamine, 200 mg/mL penicillin, 200 U/mL streptomycin and 1 mM sodium pyruvate. To maintain EGFP and OVA expressions, transfected cell cultures contained G418 (400 µg/mL for MC38mOVA, 1000 µg/mL for B16-F10mOVA and B16-F10cOVA).

BALB/c mice were obtained from the Translational Animal Research Center (TARC) of the Johannes Gutenberg-University Mainz). C57BL/6J mice were purchased from Envigo or bred inhouse. Mice carrying heterozygous *lox*P-flanked alleles for TLR7 were obtained from the European Mouse Mutant Archive (EMMA) at the Helmholtz Center Munich and were intercrossed with a CreDeleter mouse provided by Ari Waismain (Institute for Molecular Medicine, University Medical Center Mainz) to obtain mice deficient in TLR7 ($TLR7^{-/-}$). IgMi mice were also kindly provided by Ari Waisman. OT-I transgenic mice were provided by R. Holtappels-Geginat (Institute of Virology, University Medical Center, Johannes Gutenberg-University Mainz). For *in vivo* experiments, mice were 8-12 weeks old and experiments were performed in a blinded fashion. All mice were housed at the animal facility of the Johannes Gutenberg-University Mainz. Animal experiments were performed following institutional guidelines and with permission of the State Rhineland Palatinate.

Bone marrow cells were isolated from femurs and tibiae of C57BL/6 mice and resuspended in IMDM-based culture medium supplemented with 5% FBS, 2 mM L-glutamine, 100 IU/mL penicillin, 100 µg/mL streptomycin and 50 µM β-mercaptoethanol (Sigma-Aldrich, Deisenhofen, Germany) and with 10 ng/mL recombinant murine GM-CSF (R&D Systems, Wiesbaden, Germany).

OT-I splenocytes were cultured in RPMI 1640 (Thermo Fisher Scientific) supplemented with 10 % FCS, 2 mM L-Glutamine, 200 mg/mL penicillin, 200 U/mL streptomycin, 50 µM β-mercaptoethanol, in the presence of IL-2 (supernatant of XL-63 cells used at a dilution of 1:250, kindly provided by H.-C. Probst (Institute for Immunology, University Medical Center, Johannes Gutenberg-University Mainz)) and $OVA_{257-264}$ peptide (1 µg/mL) for 5 to 7 days.

Experimental

Synthesis of chain transfer agent (CTA)
pentafluorophenyl 4-cyano-4((phenylcarbonothioyl)thio)pentanoate

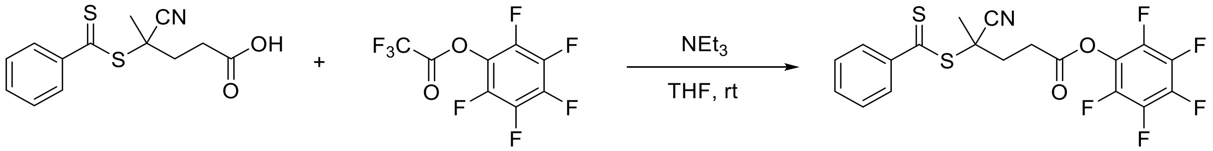

Corresponding to earlier studies[2], 4-cyano-4-((phenylcarbonothioyl)thio)pentanoic acid (2.75 g, 9.9 mmol) was dissolved under Ar-atmosphere in 90 mL anhydrous THF in a predried round bottom flask. Then, triethylamine (4.9 mL, 35.5 mmol) was added dropwise, followed by the dropwise addition of pentafluorophenyl 2,2,2-trifluoroacetate (4.4 mL, 25.6 mmol). After stirring at room temperature for 4 h the reaction mixture was supplemented with 100 mL DCM and washed two times with water. The organic layers were dried over Na_2SO_4 and concentrated in vacuum. The crude product was purified by silica column chromatography (DCM) and yielded pentafluorophenyl 4-cyano-4((phenylcarbonothioyl)thio)pentanoate as a pink solid after concentration under vacuum (3.18 g, 72%).

^{1}H-NMR ($CDCl_3$, 400 MHz): δ [ppm] = 7.93 (d, J = 8,0 Hz, 2H, ortho-Ar*H*), 7.59 (t, J = 8 Hz, 1H, p-Ar*H*), 7.42 (t, J = 8 Hz, 2H, m-Ar*H*), 3.14–3.00 (m, 2H, -COO-C*H₂*-, 2.81–2.73 (m, 1H, -COO-CH_2-CH_2-), 2.60–2.52 (m, 1H, -COO-CH_2-CH_2-), 1.99 (s, 3H, -CH_3).

^{19}F-NMR ($CDCl_3$, 282 MHz): δ [ppm] = -153.64 (d, 2F, o-Ar*F*), -158.38 (t, 1F, p-Ar*F*), -162.99 (t, 2F, m-Ar*F*).

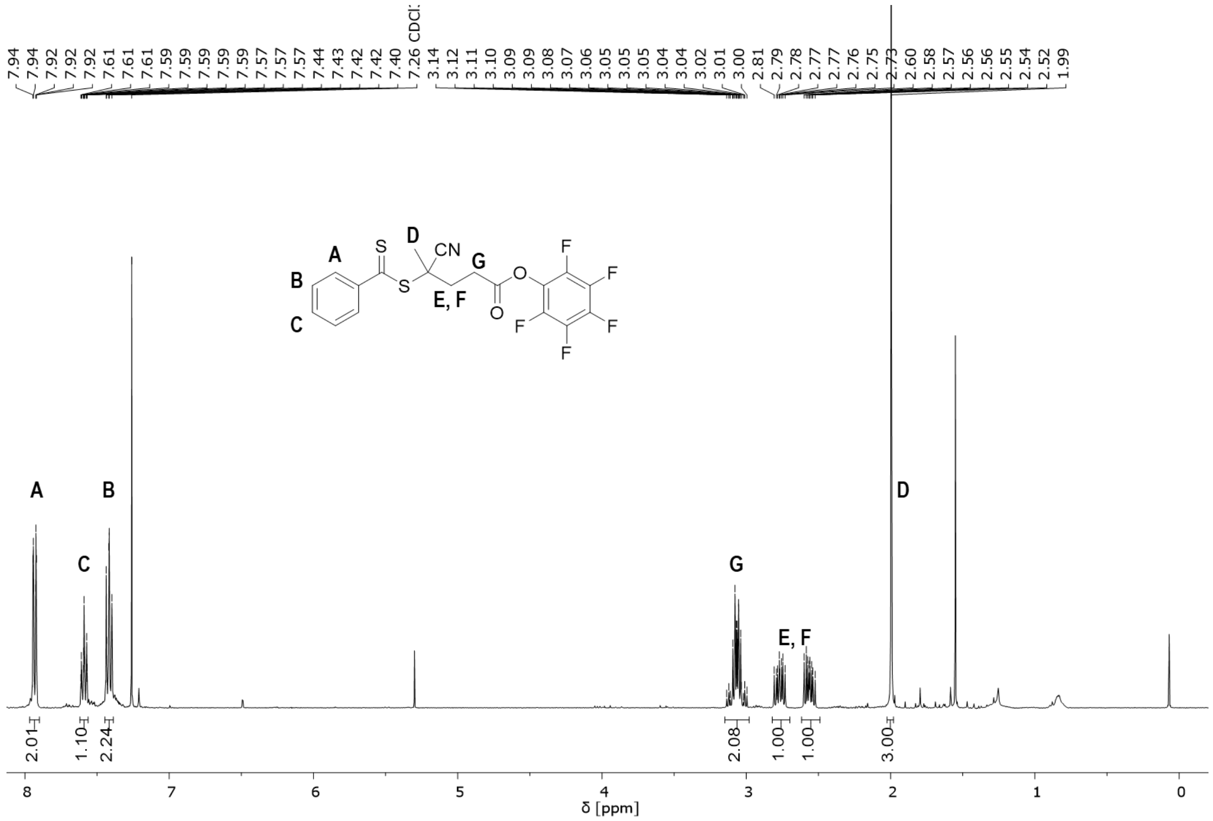

Figure S1: ^{1}H-NMR (400 MHz, $CDCl_3$) of pentafluorophenyl 4-cyano-4((phenylcarbonothioyl)thio)pentanoate.

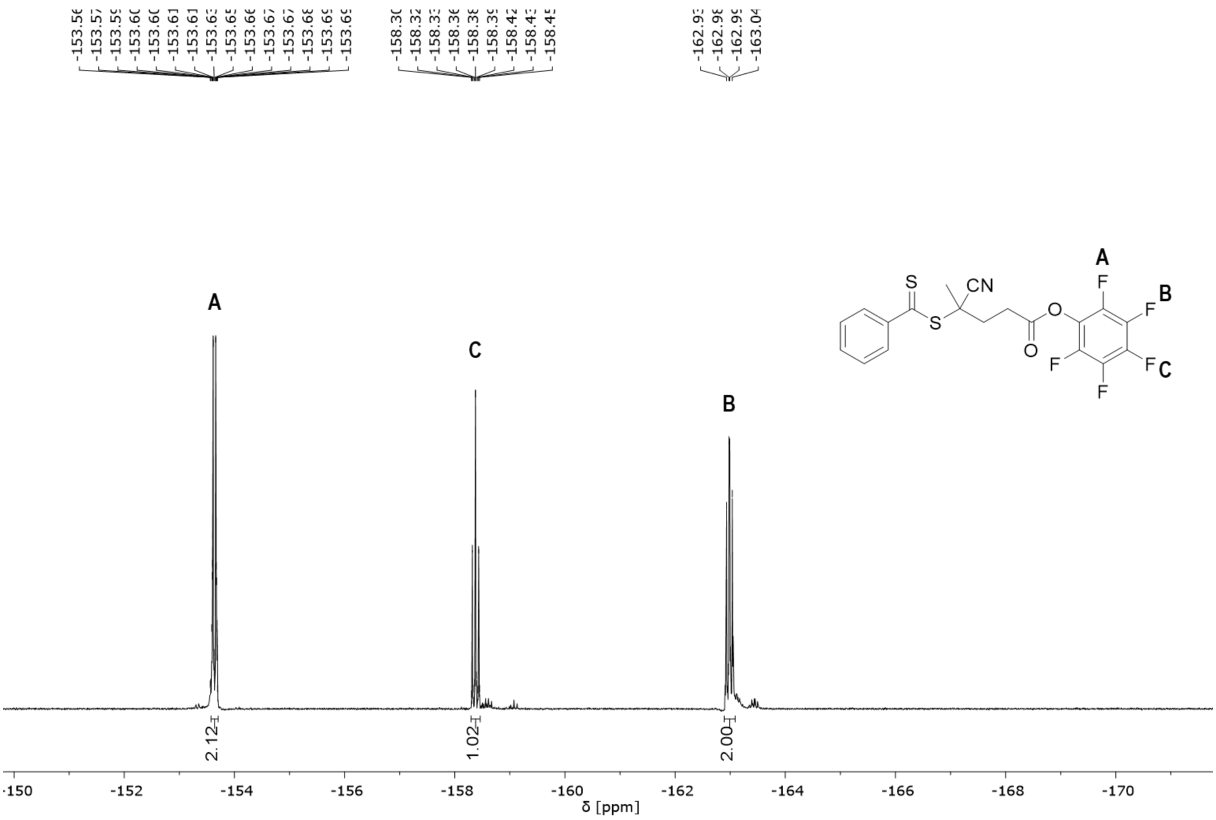

Figure S2: ^{19}F-NMR (282 MHz, $CDCl_3$) of pentafluorophenyl 4-cyano-4((phenylcarbonothioyl)thio)pentanoate.

Synthesis of azide-CTA 1-azido-16-cyano-13-oxo-3,6,9-trioxa-12-azaheptadecan-16-yl benzodithioate

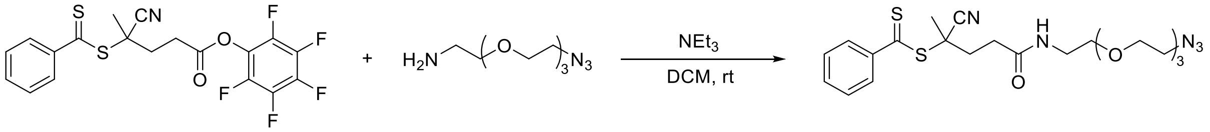

In analogy to earlier reports,[3] pentafluorophenyl 4-cyano-4((phenylcarbonothioyl)thio)pentanoate (1.2 g, 2.69 mmol) was dissolved in 15 mL anhydrous DCM under Ar-atmosphere in a predried flask. 11-Azido-3,6,9-trioxaundecan-1-amine (0.21 mL, 1.08 mmol) and triethylamine (0.45 mL, 3.23 mmol) were dissolved in 5 mL anhydrous DCM and added dropwise to the dissolved CTA. The reaction mixture was stirred in the dark for 17 h at room temperature. Reaction monitoring by TLC showed complete conversion of amine. The crude mixture was concentrated and purified by silica column chromatography (ethyl acetate : MeOH = 20:1) yielding 1-azido-16-cyano-13-oxo-3,6,9-trioxa-12-azaheptadecan-16-yl benzodithioate as a pink, viscous oil (quant.).

^{1}H-NMR ($CDCl_3$, 400 MHz): δ [ppm] = 7.90 (d, J = 8.0 Hz, 2H, o-Ar*H*), 7.56 (t, J = 8.0 Hz, 1H, p-Ar*H*), 7.39 (t, J = 8.0 Hz, 2H, m-Ar*H*), 6.32 (t, J = 4.0 Hz, 1H, -CON*H*), 3.68–3.66 (m, 8H, -O-CH_2-CH_2-O- CH_2-CH_2-O), 3.64–3.63 (m, 2H, -O-CH_2-CH_2-N_3), 3.57 (t, J = 4.0 Hz, 2H, -CONH-CH_2-CH_2-O-), 3.47 (q, J = 4.0 Hz, 2H, -CONH-CH_2-), 3.38 (t, J = 4.0 Hz, 2H, -CH_2-N_3), 2.67–2.60 (m, 1H, -CH*H*-CH_2-CONH-), 2.58–2.51 (m, 2H, -CH_2-CONH-), 2.45–2.38 (m, 1H, -C*H*H-CH_2-CONH-), 1.94 (s, 3H, -CH_3).

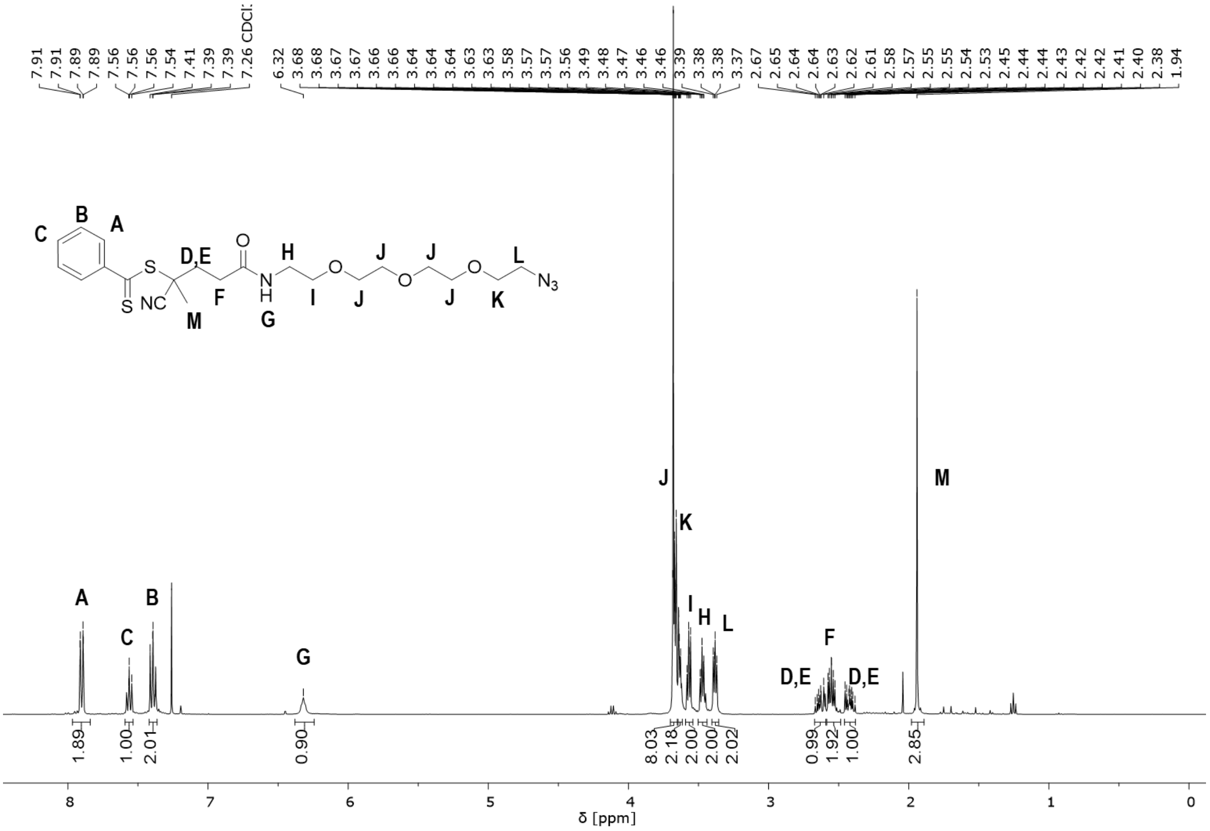

Figure S3: ^{1}H-NMR (400 MHz, $CDCl_3$) of azide-CTA 1-azido-16-cyano-13-oxo-3,6,9-trioxa-12-azaheptadecan-16-yl benzodithioate.

Synthesis of amphiphilic block copolymer P(mTEGMA)-b-P(PFPMA) by RAFT block copolymerization

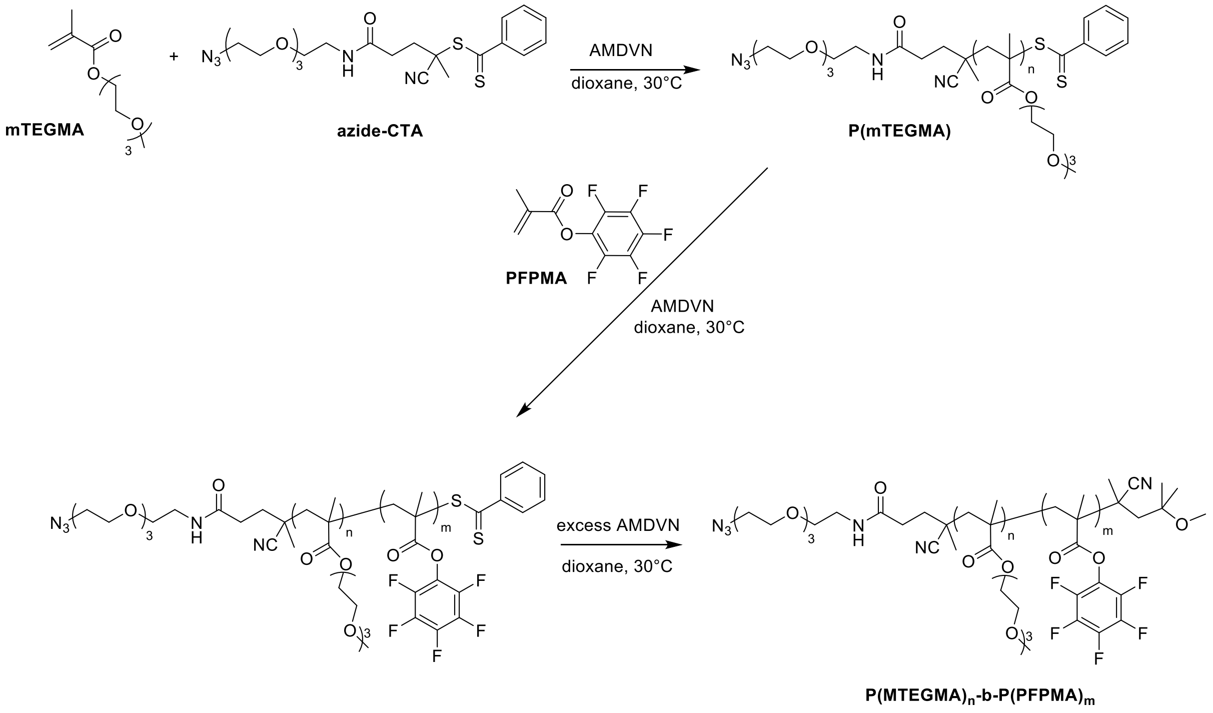

Synthesis of poly(tri(ethylene glycol)methyl ether methacrylate) P(mTEGMA)

P(mTEGMA) was synthesized via RAFT polymerization as reported previously.[4] 1-azido-16-cyano-13-oxo-3,6,9-trioxa-12-azaheptadecan-16-yl benzodithioate (azide-CTA) (92.5 mg, 0.192 mmol) and tri(ethyleneglycol)methyl ether methacrylate/mTEGMA (896 mg, 3.857 mmol) were loaded into a Schlenk tube and dissolved in 2 mL anhydrous 1,4-dioxane. Low temperature initiator 2,2′-azobis(4-methoxy-2,4-dimethylvaleronitrile) (AMDVN) (5.95 mg, 0.019 mmol) was added. After performing three freeze pump thaw cycles the reaction mixture was stirred at 30°C for 16 h. Analysis of a reaction aliquot by ^{1}H-NMR revealed a monomer conversion of 74%. The polymer was then isolated by precipitation in hexane followed by centrifugation. After re-dissolving the polymer in 2 mL dioxane the precipitation procedure was repeated two more times. Drying under vacuum overnight afforded P(mTEGMA) as a red oil (592 mg, 78%). End group analysis based on ^{1}H-NMR showed a chain length χ_n of 25 for P(mTEGMA).

^{1}H-NMR ($CDCl_3$, 400 MHz): δ [ppm] = 4.08 (br, 2H, -COO-CH_2), 3.64 (br, 8H, -COO-CH_2-CH_2-O-CH_2-CH_2-O-CH_2-), 3.55 (br, 2H, -CH_2-O-CH_3), 3.38 (br, 3H, -O-CH_3), 2.12–1.67 (br, 2H, -CH_2- polymer main chain), 1.38–0.64 (br, 3H, -CH_3 polymer main chain).

SEC (THF, PS-Std.): M_n = 5050 g/mol , M_w = 6160 g/mol , PDI = 1.22.

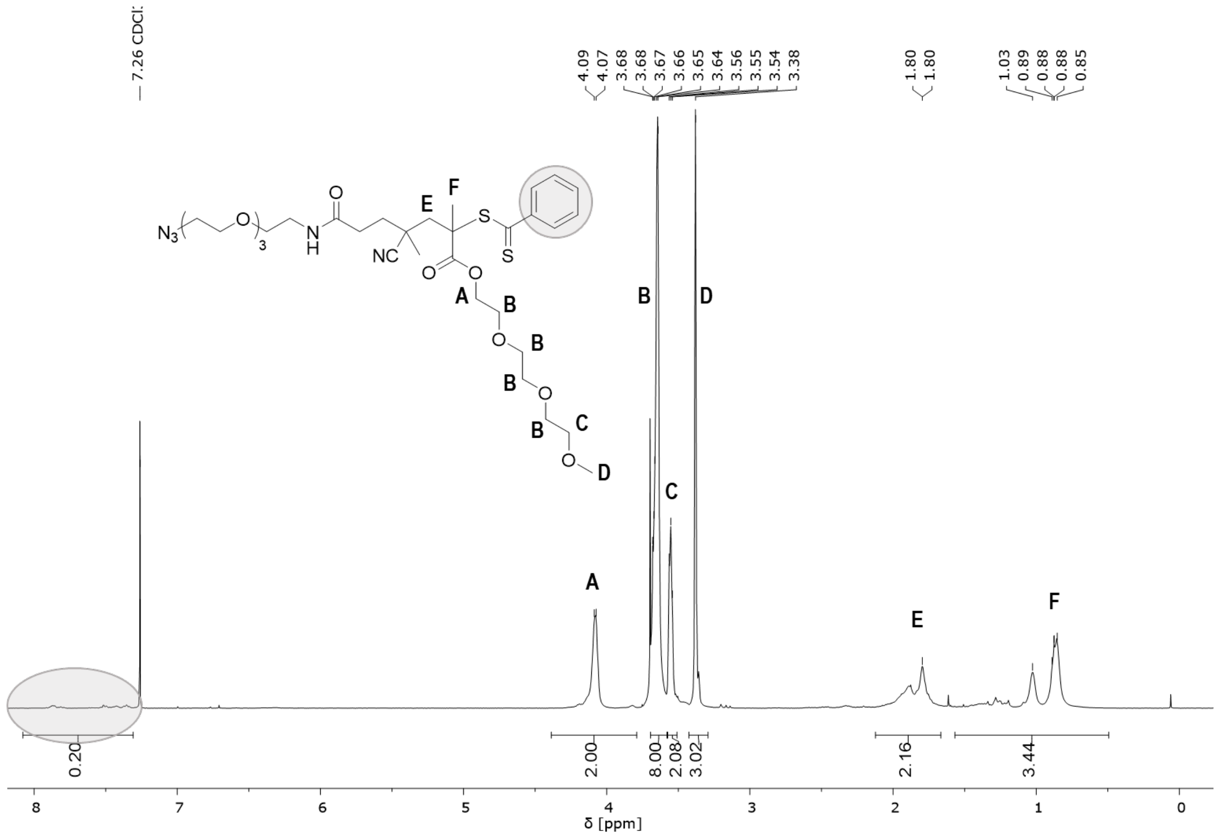

Figure S4: ^{1}H-NMR (400 MHz, $CDCl_3$) of P(mTEGMA).

Synthesis of poly(tri(ethylene glycol)methyl ether methacrylate)–block–poly(pentafluorophenyl methacrylate) P(mTEGMA)-b-P(PFPMA)

Corresponding to earlier reports[4], P(mTEGMA) as macro-CTA (578 mg, 0.113 mmol) and pentafluorophenyl methacrylate (PFPMA) (1280 mg, 5.1 mmol) were dissolved in 2 mL anhydrous dioxane in a Schlenk tube with a stirring bar. After addition of AMDVN (3.4 mg, 0.01 mmol) three freeze pump thaw cycles were performed. The degassed reaction mixture was then stirred for 40 h at 30°C. Analysis by ^{1}H-NMR and ^{19}F-NMR of a reaction aliquot in $CDCl_3$ revealed a PFPMA-conversion around 50%. The polymer was purified by repeated precipitation in hexane and centrifugation (3 times). Drying overnight under vacuum afforded P(mTEGMA)-*b*-P(PFPMA) as a red powder (1.3 g, quant.).

Removal of dithiobenzoate end groups of P(mTEGMA)-b-P(PFPMA)

Removal of dithiobenzoate end groups of previously synthesized P(mTEGMA)-*b*-(PFPMA) was achieved by treating the polymer with an excess of the initiator AMDVN. Therefore, P(mTEGMA)-*b*-P(PFPMA) (1.33 g, 87.9 µmol) was dissolved in 2 mL anhydrous dioxane in a Schlenk tube with a stir bar. After addition of AMDVN (813 mg, 264 µmol) the reaction mixture was stirred overnight under nitrogen atmosphere at 30°C. Successful removal of dithiobenzoate end groups was observed by a loss of red color of the reaction mixture and was confirmed by UV-Vis spectroscopy. The colorless polymer was purified by precipitation in hexane followed by centrifugation. The precipitation process was repeated two times after re-dissolving the polymer in 2 mL dioxane. Vacuum drying afforded P(mTEGMA)-*b*-P(PFPMA) as a colorless powder (946 mg). Copolymer composition was analyzed based on ^{1}H-NMR spectra of P(mTEGMA)-*b*-P(PFPMA) by normalization to the methoxy group of P(mTEGMA)

followed by a comparison of signals of polymer main chains of P(mTEGMA) and P(PFPMA) blocks. Referring to a block copolymer composition of P(mTEGMA) : P(PFPMA) = 1 : 1.36 a chain length χ_n of 34 was found for P(PFPMA).

^{1}H-NMR ($CDCl_3$, 400 MHz): δ [ppm] = 4.08 (br, 2H, -COO-CH_2), 3.65 (br, 8H, -COO-CH_2-CH_2-O-CH_2-CH_2-O-CH_2-), 3.56 (br, 2H, -CH_2-O-CH_3), 3.38 (br, 3H, -O-CH_3), 2.71–2.00 (br, 2H, -CH_2- PFPMA polymer main chain), 1.90–1.67 (br, 2H, -CH_2- mTEGMA polymer main chain), 1.64–1.23 (br, 3H, -CH_3 PFPMA polymer main chain), 1.16–0.62 (br, 3H, -CH_3 mTEGMA polymer main chain).

^{19}F-NMR ($CDCl_3$, 282 MHz): δ [ppm] = -150.90–153.36 (br, 2F, o-Ar*F*), -158.09 (br, 1F, p-Ar*F*), -163.27 (br, 2F, m-Ar*F*).

SEC (THF, PS-Std.): M_n = 12050 g/mol, M_w =15950 g/mol, PDI = 1.33.

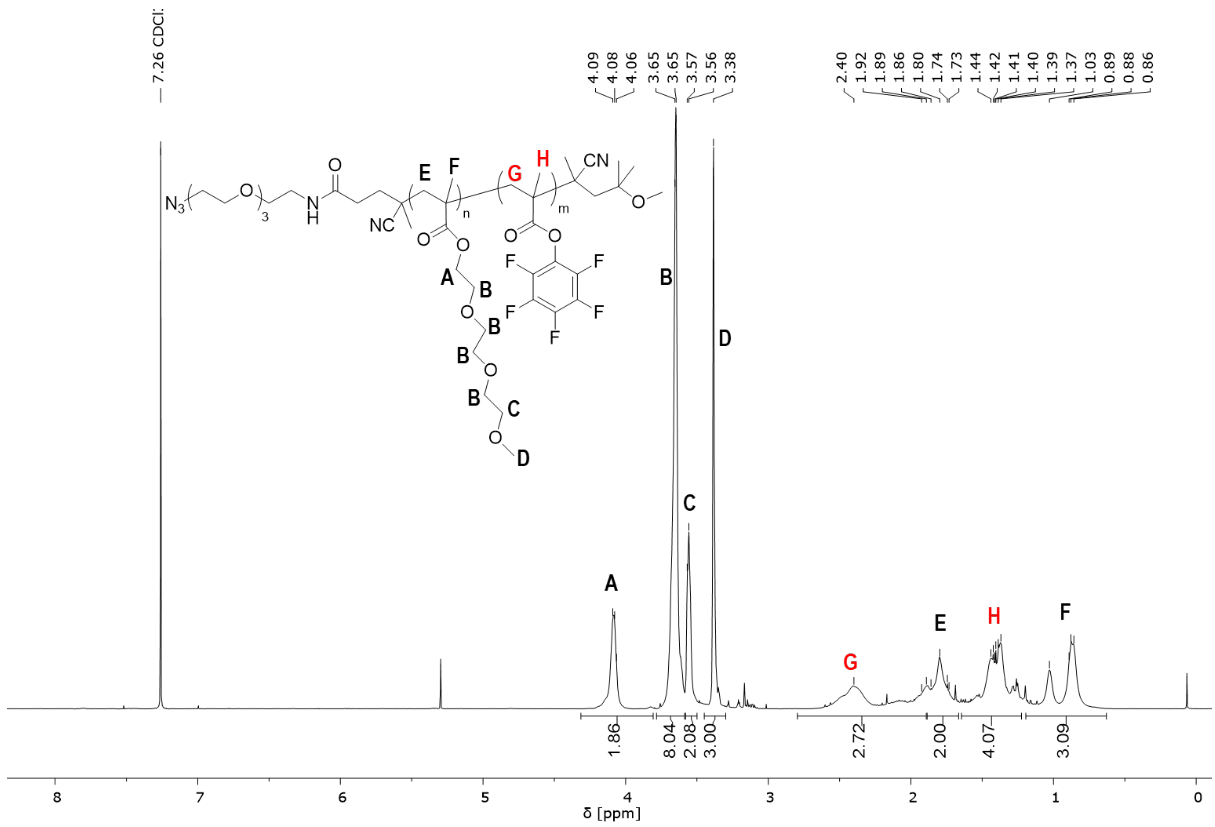

Figure S5: ^{1}H-NMR ($CDCl_3$, 400 MHz) of P(mTEGMA)-b-P(PFPMA) after RAFT end group removal.

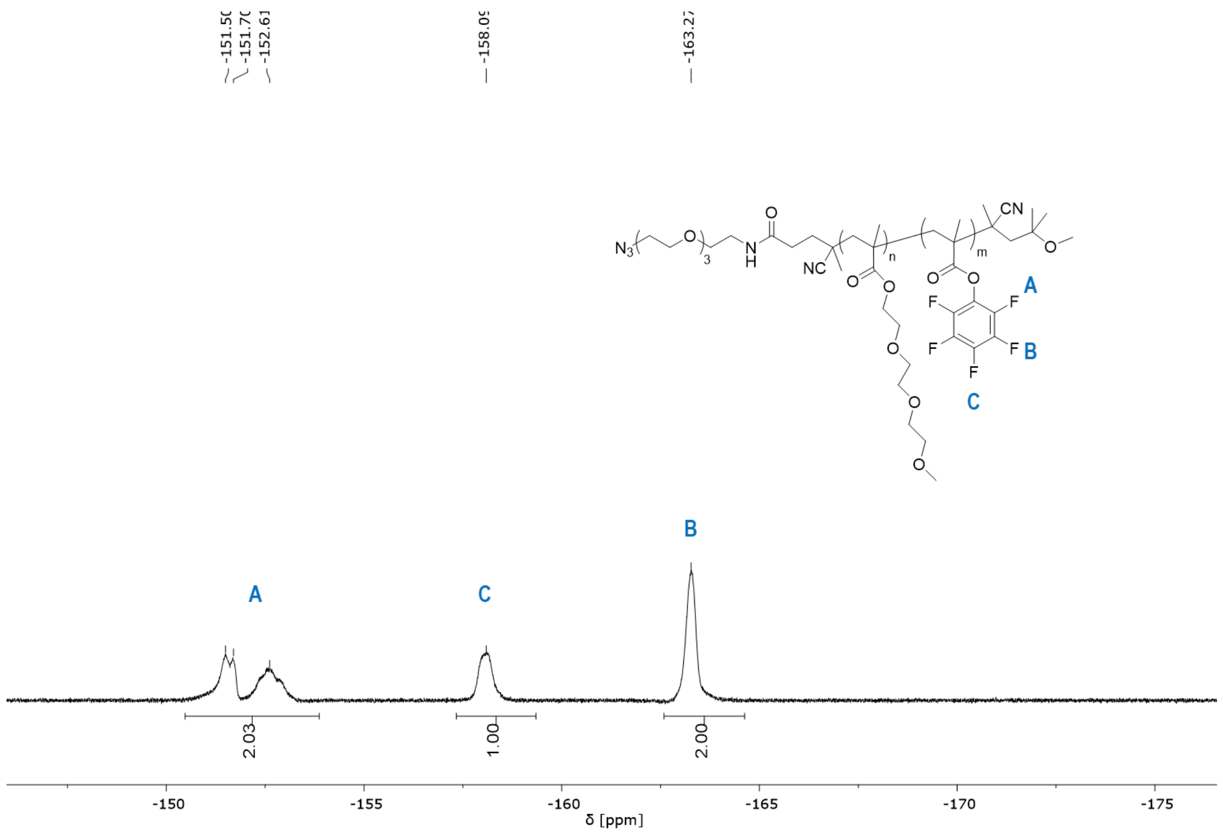

Figure S6: ^{19}F-NMR ($CDCl_3$, 282 MHz) of P(mTEGMA)-b-P(PFPMA) after RAFT end group removal.

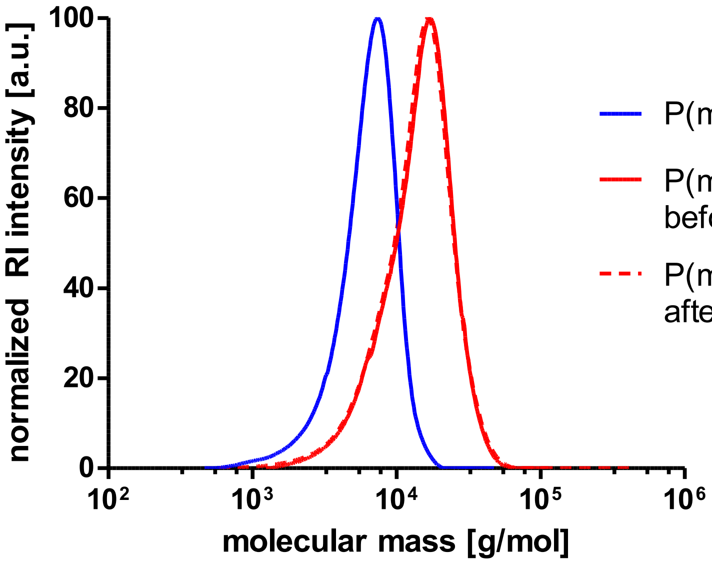

Figure S7: SEC trace (THF. PS-Std.) of P(mTEGMA) and P(mTEGMA)-b-P(PFPMA) before and after RAFT end group removal.

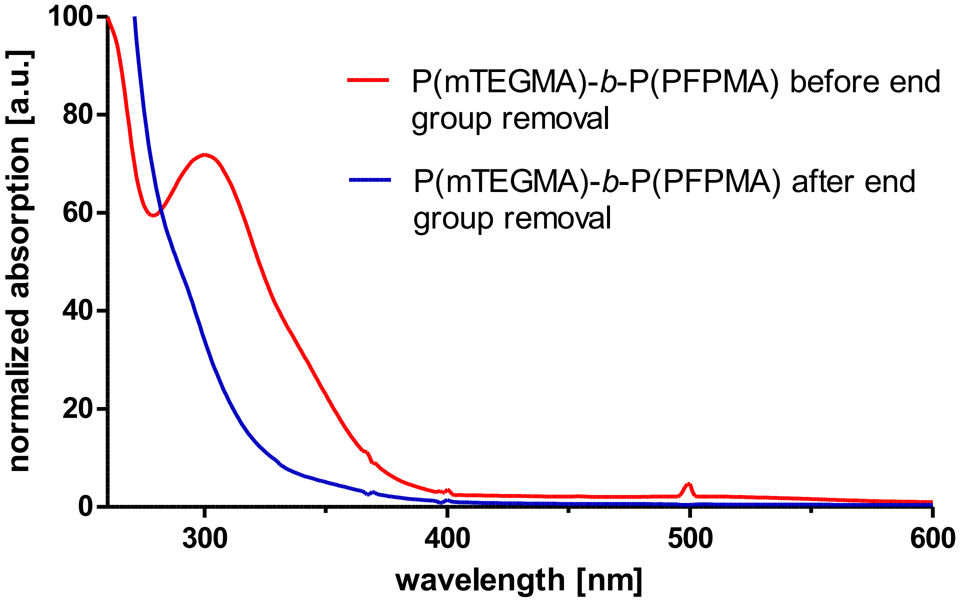

Figure S8: UV-Vis spectra of P(mTEGMA)-b-P(PFPMA) in dioxane before and after removal of RAFT end groups.

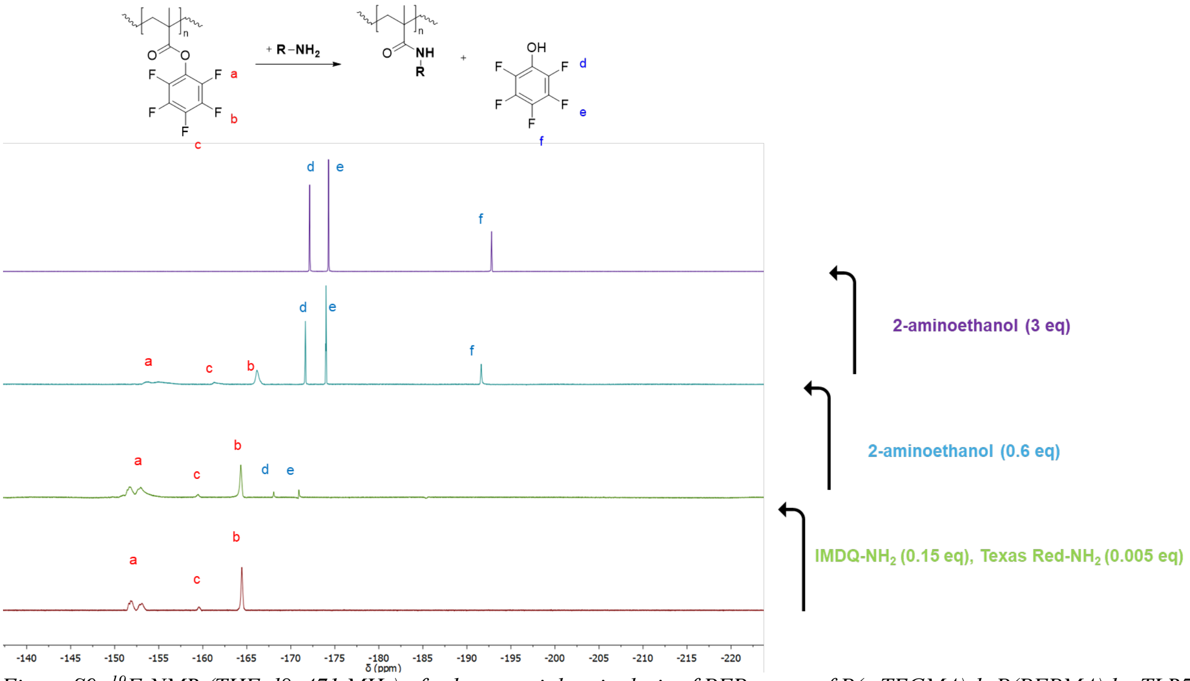

Figure S9: ^{19}F-NMR (THF-d8, 471 MHz) of subsequential aminolysis of PFP esters of P(mTEGMA)-b-P(PFPMA) by TLR7/8 agonist IMDQ and fluorescent dye followed by hydrophilization with 2-aminoethanol.

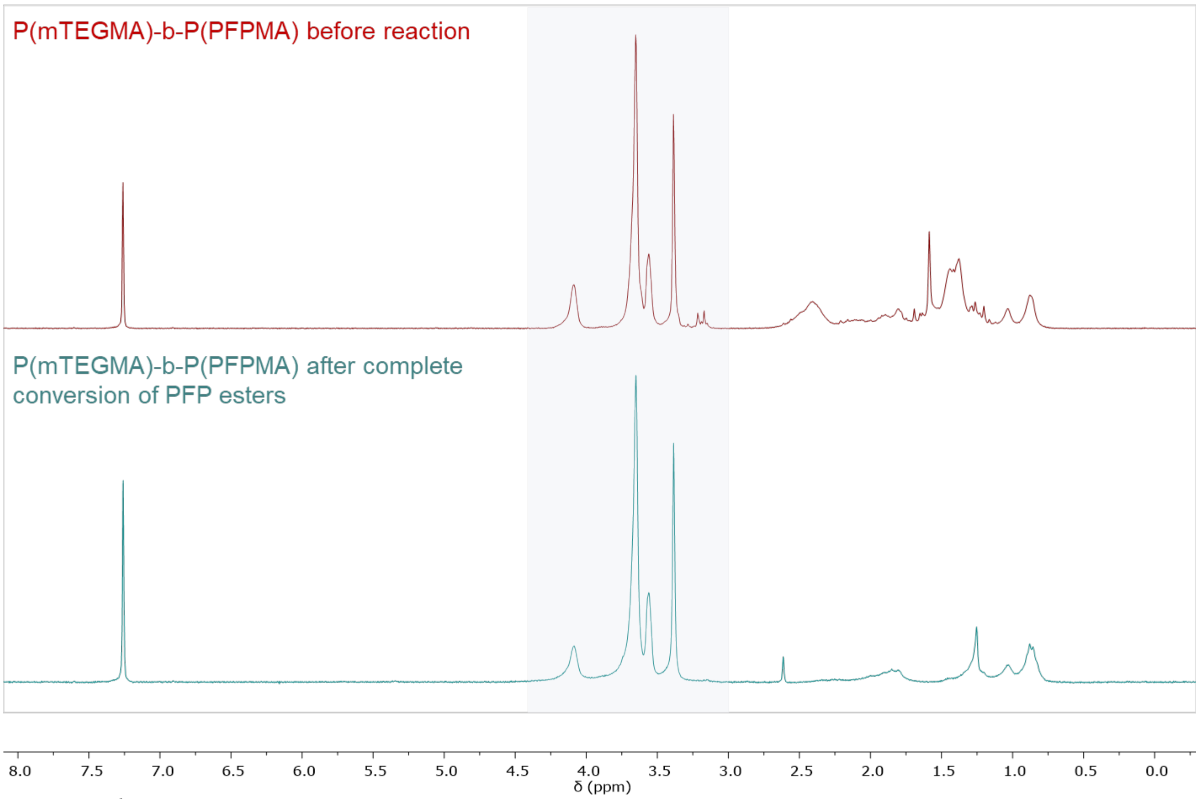

Figure S10: ^{1}H-NMR (CDCl3, 300 MHz) of P(mTEGMA)-b-P(PFPMA) before and after conversion of PFP esters with IMDQ and 2-ethanolamine. Ester bonds of the P(mTEGMA) segment show no reactivity towards added amines during aminolysis of PFP esters,

Nanogel synthesis

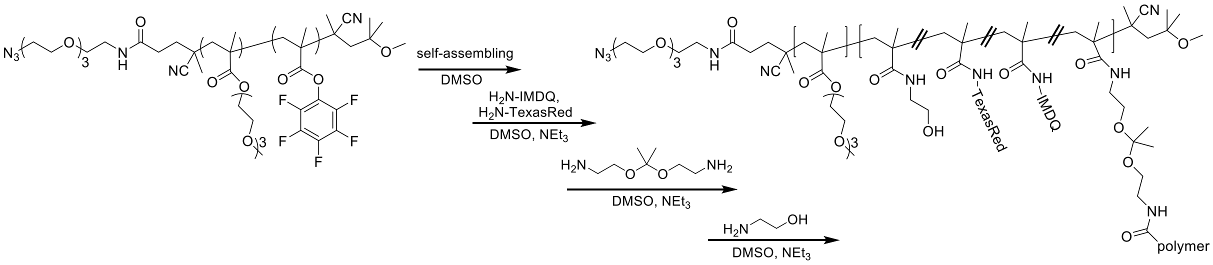

Two different types of acid-degradable, fluorescently labeled nanogels (IMDQ-loaded or without IMDQ/control) were prepared based on a modified procedure from earlier reports.[5] The reactive precursor polymer P(mTEGMA)-*b*-P(PFPMA) was dispersed at 10 mg/mL in anhydrous DMSO under nitrogen atmosphere followed by sonication for 1h. Formation of polymeric micelles was verified by DLS measurements and micellar dispersions were further used for the nanogel synthesis.

For each nanogel type a micellar dispersion of P(mTEGMA)-*b*-P(PFPMA) (10 mL, 230 µmol reactive ester) was transferred into a Schlenk tube equipped with a stir bar. For fluorescent labeling, Cy5 cadaverine or Texas Red cadaverine (2.5 mg/mL, 317 µL, 1.15 µmol) was added under nitrogen atmosphere. In case of covalent loading with the immune modulator IMDQ, IMDQ (10 mg/mL, 1490 µL, 34.5 µmol) was added. After the addition of triethylamine (48 µL, 345 µmol) the reaction mixture was stirred under nitrogen atmosphere at 40°C for 48 h. To achieve pH-degradable crosslinking, 2,2-bis(aminoethoxy)propane (10.1 µL, 68.9 µmol) and triethylamine (96 µL, 689 µmol) were added and the reaction mixture was stirred under nitrogen atmosphere at 50°C for 24 h. To obtain fully hydrophilic nanogels the reaction mixture was quenched with 2-aminoethanol (41.6 µL, 689 µmol) and triethylamine (96 µL, 689 µmol) and stirred for a half day at 50°C. Then, nanogel preparations were transferred into dialysis membranes (MWCO 3.5 kDa) and dialyzed against water supplemented with 0.1 % (v/v) ammonia for three days. Lyophilization afforded nanogels as voluminous solids (77 mg, 91%). Lyophilized nanogels were re-dispersed in water supplemented with 0.1% (v/v) ammonia or PBS at given concentrations followed by sonication for 1 h under ice cooling prior to application in described experiments. IMDQ-loading was determined based on a calibration curve and adjusted equations from literature and revealed a drug-loading of 9.4 wt%.[5]

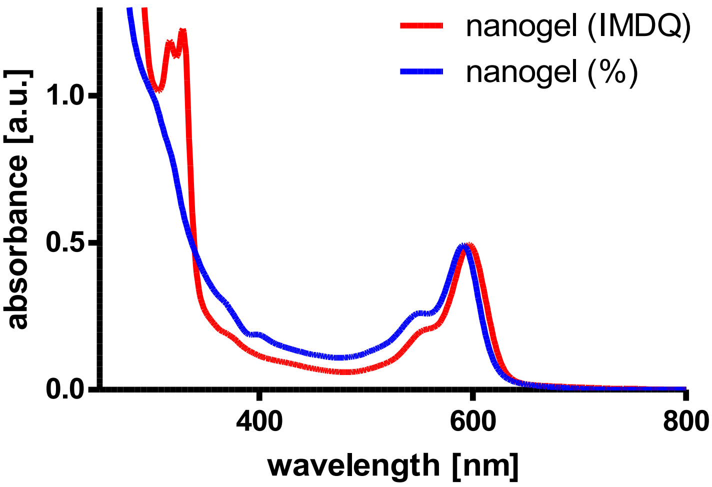

Figure S11: UV-Vis spectra of re-dispersed nanogel samples at 1 mg/mLfor determination of IMDQ-loading.

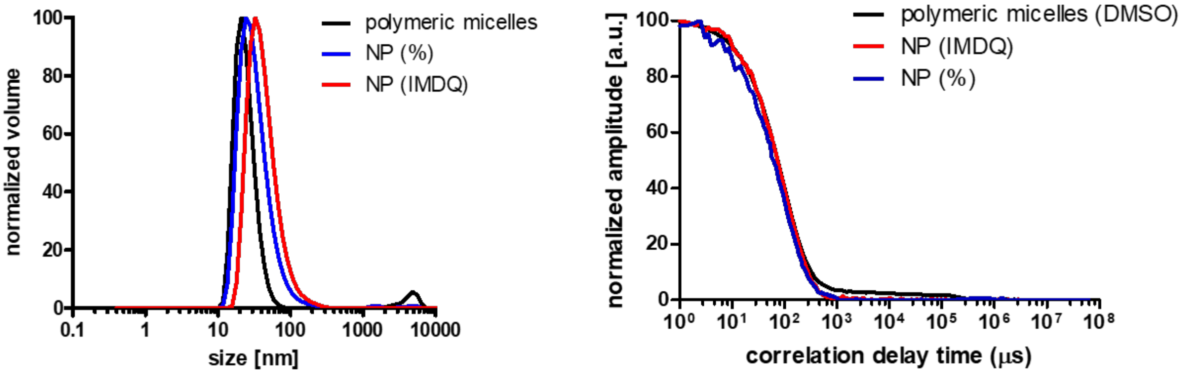

0.5 mg/mL	z-average [nm]	PDI
**polymeric micelle**	32.5 ± 0.2	0.25 ± 0.05
**nanogel (%)**	58.1 ± 1.9	0.38 ± 0.05
**nanogel (IMDQ)**	67.4 ± 2.0	0.23 ± 0.03

Figure S12: DLS results of polymeric micelles in DMSO (10 mg/mL) and resulting hydrophilic nanogels with or without (%) IMDQ at 0.5 mg/mL PBS.

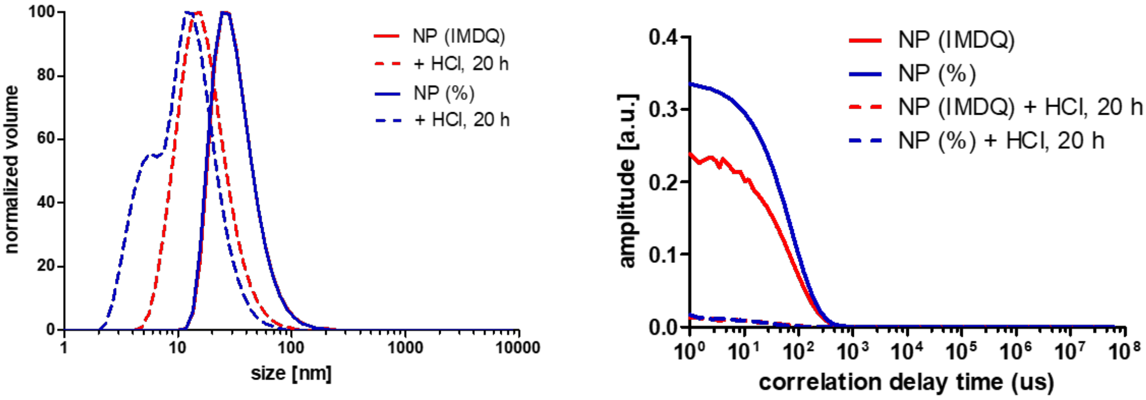

	z-average [nm]	PDI	volume mean [nm]
**NP (%)**	51.7 ± 1.3	0.209 ± 0.003	34.1 ± 1.0
+ HCl, 20 h	24.0 ± 1.8	0.312 ± 0.110	10.1 ± 7.1
**NP (IMDQ)**	53.8 ± 0.7	0.216 ± 0.008	34.6 ± 1.4
+ HCl, 20 h	33.0 ± 1.5	0.218 ± 0.056	18.5 ± 3.7

Figure S13: DLS study of nanogel degradation under acidic conditions at 1 mg/mL. 10 v% 1M HCl was added to each nanogel solution.

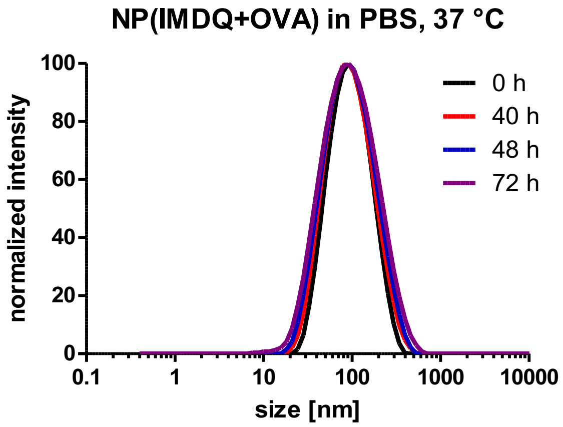

Figure S14: DLS study of nanogel stability under physiological conditions at 37°C. Nanogels are stable for at least 72 h.

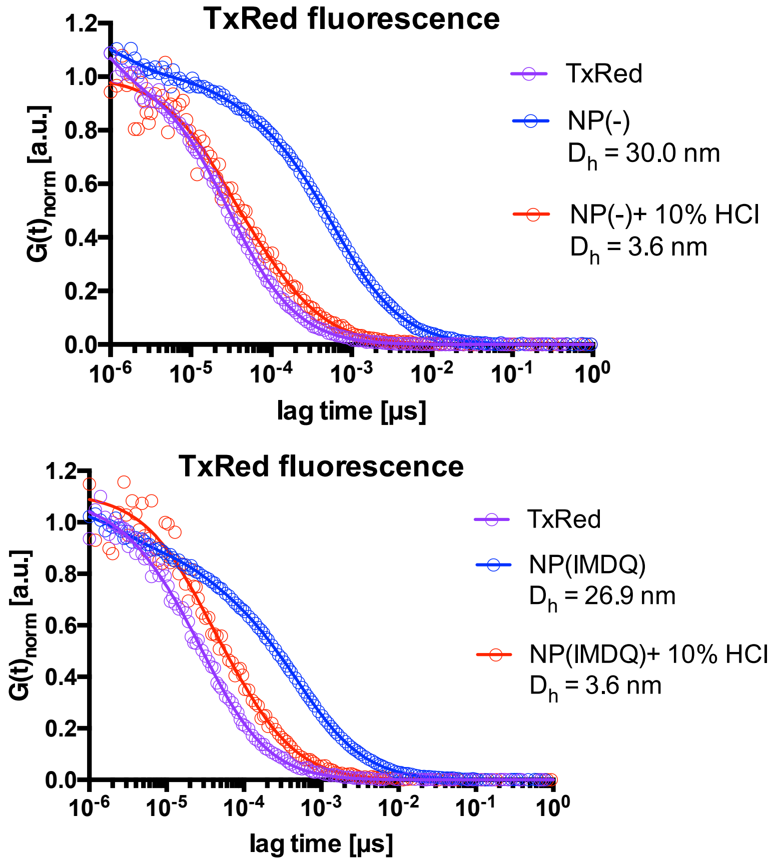

Figure S15: FCS study of empty and IMDQ-loaded nanogel degradation under acidic conditions at 2.4 µg/mL. in PBS alone or supplemented with 10 v% 1M HCl to each nanogel solution.

To ensure azide-accessibility nanogels were applied in a SPAAC reaction with a cyclooctyne-modified dye prior to antigen conjugation. To this regard, cyclooctyne tetra(ethylene oxide)-*N*-hydroxysuccinimidyl-ester (NHS ester) (10 mg/mL, 5.7 µL, 128.8 nmol), derived from earlier reports,[3] was added to Oregon Green cadaverine (2.5 mg/mL, 25.1 µL, 128.8 nmol) together with triethylamine (0.25 µL) and shaken overnight at room temperature (as control, an Oregon Green cadaverine solution was prepared at same concentration in DMSO with triethylamine, but without addition of cyclooctyne tetra(ethylene oxide)-NHS ester). To both samples (cyclooctyne-modified Oregon Green and non-modified Oregon Green) a solution of nanogel (2 mg/mL PBS, 250 µL) was then added and the reaction mixture was incubated overnight at room temperature. Repetitive centrifugal filtration with water supplemented with 0.1% (v/v) ammonia was used to purify SPAAC-conjugates from free Oregon Green dye. Successful conjugation could be monitored by UV-Vis spectroscopy.

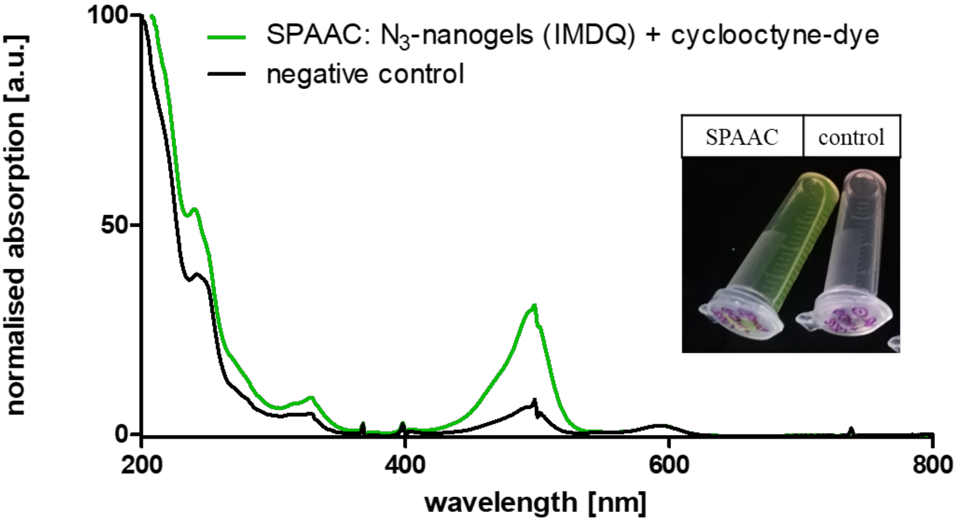

Figure S16: UV-Vis spectra and photograph of SPAAC reaction between IMDQ-loaded nanogels and cycloocytane-modified Oregon Green dye after several steps of centrifugal filtration.

Fluorescent labeling and modification of model-antigen ovalbumin with DBCO-PEG4-linker for SPAAC reaction

The sequence of ovalbumin can be found on PDB (https://www.rcsb.org/pdb/explore/remediatedSequence.do?structureId=1OVA):

Ac-
GSIGAASMEFCFDVFKELKVHHANENIFYCPIAIMSALAMVYLGAKDSTRTQINKVV RFD
KLPGFGDS[phos]IEAQCGTSVNVHSSLRDILNQITKPNDVYSFSLASRLYAEERYPILPEY LQCVKELYRGGLEPINFQTAADQARELINSWVESQTNGIIRNVLQPSSVDSQTAMVL VNAIVFKGLWEKAFKDEDTQAMPFRVTEQESKPVQMMYQIGLFRVASMASEKMKI LELPFASGTMSMLVLLPDEVSGLEQLE[*SIINFEKL*][MHC I epitope]TEWTSSNVMEERKIKV YLPRMKMEEKYN[N_acetylglucosamine]LTSVLMAMGITDVFSSSANLSGISSAESLK[*ISQAVH AAHAEINEAGR*][MHC II epitope]EVVG*S*[*phos*]AEAGVDAASVSEEFRADHPFLFCIKHIATNAVLF FGRCVSP.

Amon the 386 amino acids affording a molecular weight of 42.8 kDa there are 20 lysine residues as potential modification sites for NHS-ester derived amidations. Only one potential modification site is located in the MHC-I sequence (*SIINFEKL*) but none can be found inside the MHC-II sequence (*ISQAVHAAHAEINEAGR*).

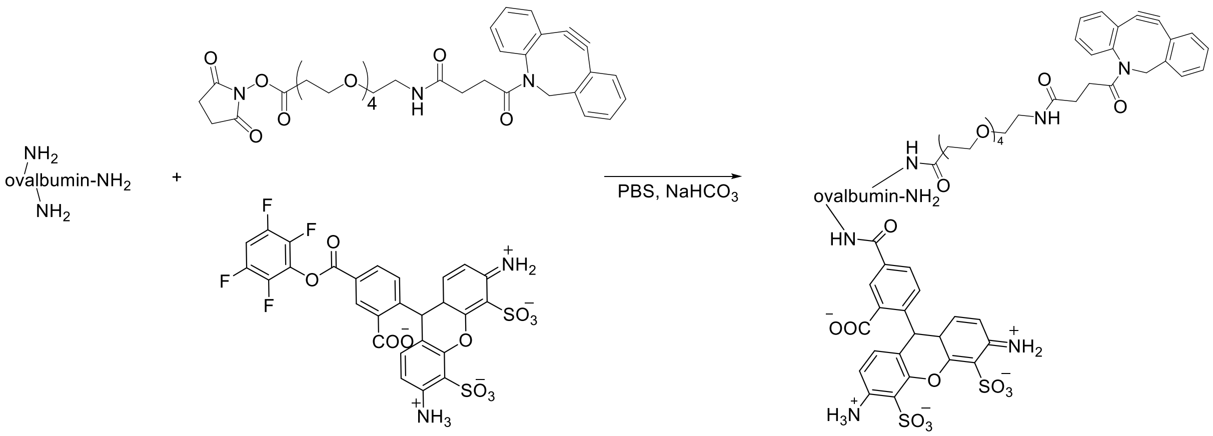

Fluorescent labeling with Alexa Fluor 488 and the introduction of a SPAAC-reactive DBCO-PEG4-linker was performed simultaneously at the amino-containing side chains of ovalbumin. To a solution of ovalbumin in sterile, endotoxin-free water (2 mg/mL, 500 µL, 23.4 nmol) supplemented with bicarbonate buffer (1 M, 50 µL, pH 8.3) Alexa Fluor 488-tetrafluorophenyl ester (2 mg/mL DMSO, 35 µL, 79 nmol) was added. In case of desired DBCO-modification dibenzylcylooctyne-PEG4-NHS ester (10 mg/mL DMSO, 11.4 µL, 175 nmol) was added, too. The reaction mixture was vigorously stirred in the dark at room temperature for 4 h and the purified by size-exclusion chromatography under sterile conditions (eluent: sterile PBS). Protein concentration was determined using Micro BCA Protein Assay (Thermo Fisher) with a standard curve generated from ovalbumin samples of known concentrations.

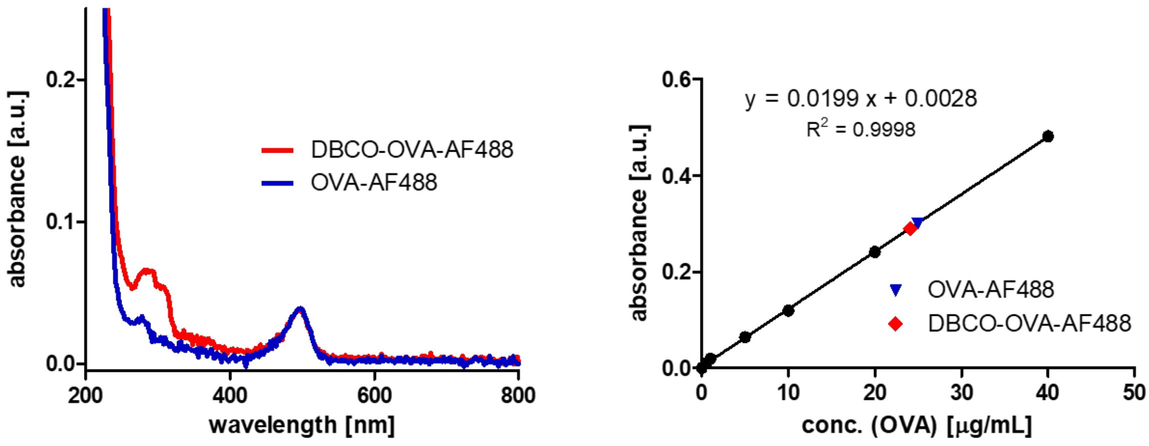

Figure S17: UV-Vis spectra and calibration curve for determination of protein concentration of fluorescently labeled ovalbumin with and without additional introduction of a SPAAC-reactive DBCO-linker.

The degree of labeling (DL) can be estimated by the absorbance maxima at 496 nm and 280 nm (the extinction coefficients of Alexa Fluor 488 (AF488) and DBCO-PEG$_4$-linker (DBCO) are provided by the supplier):

For OVA-AF488 DL_{AF488} can be estimated by $\frac{A_{496nm}}{A_{280nm}} = \frac{DL_{AF488} \cdot \varepsilon_{AF488\ at\ 496\ nm}}{DL_{AF488} \cdot \varepsilon_{AF488\ at\ 280\ nm} + \varepsilon_{OVA\ at\ 280\ nm}}$

$$=> DL_{AF488} = \frac{\frac{A_{496nm}}{A_{280nm}} \cdot \varepsilon_{OVA\ at\ 280\ nm}}{\varepsilon_{AF488\ at\ 496\ nm} - \frac{A_{496nm}}{A_{280nm}} \cdot \varepsilon_{AF488\ at\ 280\ nm}} = \frac{\frac{0.04}{0.03} \cdot 30590}{71000 - \frac{0.04}{0.03} \cdot 7800} \approx 1$$

For DBCO-OVA-AF488 DL_{DBCO} can be estimated assuming the same $DL_{AF488} \approx 1$ by

$$\frac{A_{496nm}}{A_{280nm}} = \frac{DL_{AF488} \cdot \varepsilon_{AF488\ at\ 496\ nm}}{DL_{AF488} \cdot \varepsilon_{AF488\ at\ 280\ nm} + DL_{DBCO} \cdot \varepsilon_{DBCO\ at\ 280\ nm} + \varepsilon_{OVA\ at\ 280\ nm}}$$

$$=> DL_{DBCO} = \frac{\frac{A_{280nm}}{A_{496nm}} \cdot DL_{AF488} \cdot \varepsilon_{AF488\ at\ 496\ nm} - DL_{AF488} \cdot \varepsilon_{AF488\ at\ 280\ nm} - \varepsilon_{OVA\ at\ 280\ nm}}{\varepsilon_{DBCO\ at\ 280\ nm}}$$

$$= \frac{\frac{0.06}{0.04} \cdot 1 \cdot 71000 - 1 \cdot 7800 - 30590}{13100} \approx 5$$

By FCS (see description below) only a slight increase in size was observed between DBCO-OVA-AF488 and OVA-AF488 confirming successful ligation of the SPAAC-reactive DBCO-PEG_4-linker, yet, this did not interfere with protein integrity or stability as no aggregates were found in PBS.

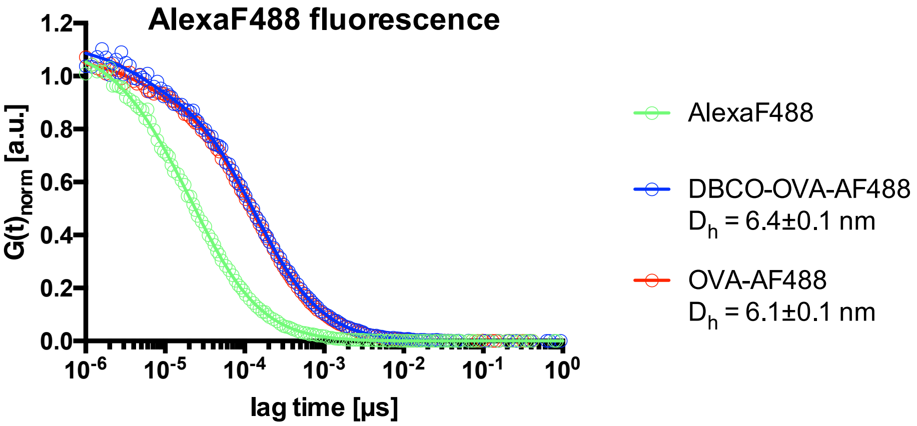

Figure S18: FCS correlogram of AF488 and SPAAC-reactive DBCO-linker modified ovalbumin.

SDS-PAGE was used to further verify OVA-DBCO modification (see description below). Samples were prepared by mixing 4.5µl of a the 0.5 µg/µg modified OVA samples (OVA-AF488 or DBCO-OVA-AF488) with 6.5 µl of a 10 µg/µl 5kDA N_3-PEG-OH sample in PBS (corresponding controls were prepared at similar concentrations) and incubated overnight at room temperature. Samples were then treated with 4x reducing Laemmli sample buffer before loading.

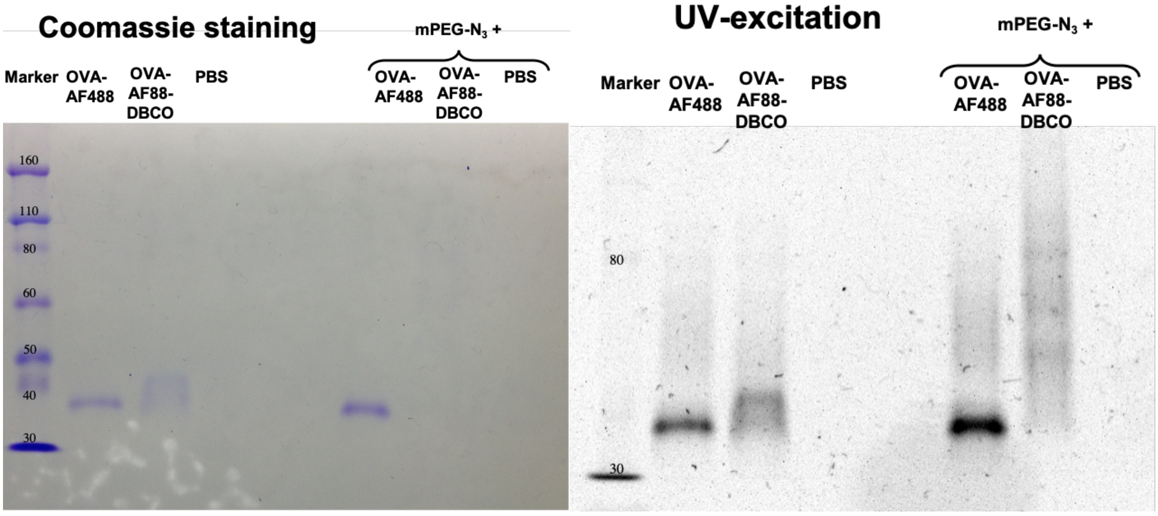

Figure S19: SDS-PAGE of AF488 and SPAAC-reactive DBCO-linker modified ovalbumin alone and incubated with azide-terminated mPEG-N_3 (5kDA). By both Coomassie staining and UV-excitation to visualize AF488-labeling successful DBCO-protein modification could be found via SPAAC-selective PEGylation.

T-cell proliferation was further applied to assure antigen bioactivity after ovalbumin-modification with AF488 and DBCO linker. For that purpose, BMDCs were incubated with OVA-AF488 and OVA-AF488-DBCO and TLR7/8 agonist IMDQ at 1µg/mL, harvested on the next day and then serially diluted and incubated with either CD8^{+} T cells from OT-I mice or CD4^{+} T cells from OT-II mice as indicated (see description below). No difference between the two samples was obtained.

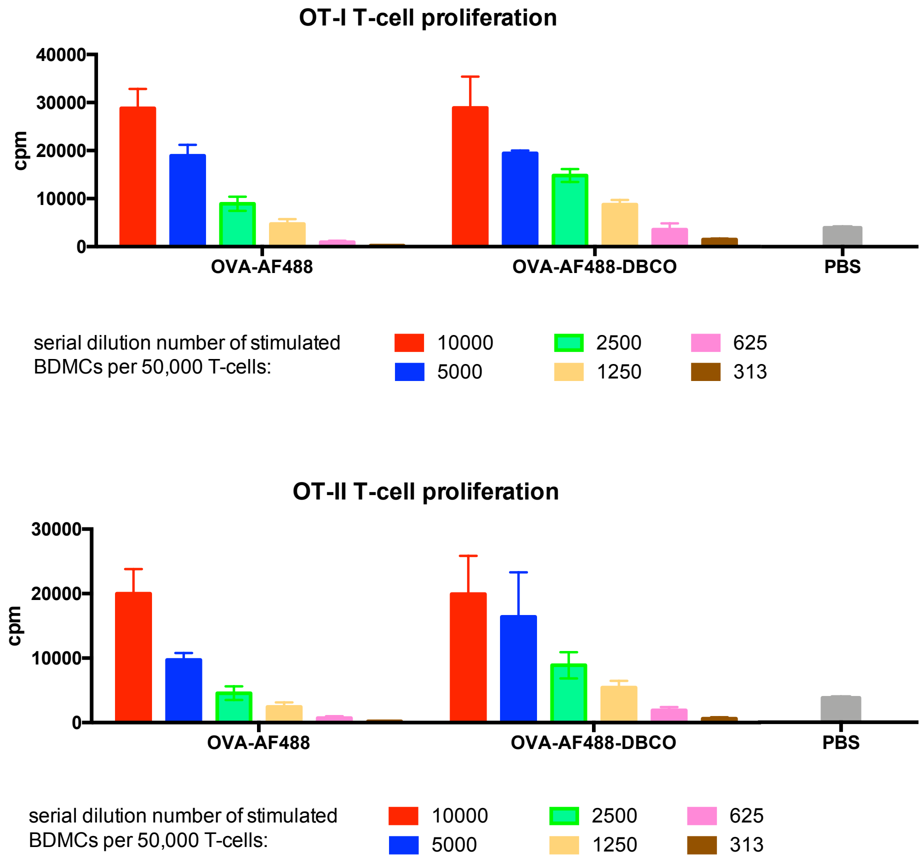

Figure S20: CD8^{+} and CD4^{+} specific T-cell proliferation derived from OT-I and OT-II T cells incubated with serial dilutions of BMDCs pulsed with OVA-AF488 and OVA-AF488-DBCO.

Surface decoration of azide-exposing nanogels by SPAAC reaction

For detailed *in vitro* and *in vivo* studies different nanogel formulations were prepared for each experiment freshly at final concentrations of 0.3 mg/mL ovalbumin and 0.1 mg/mL IMDQ. To covalently attach the antigen onto the nanogel surface, lyophilized nanogels (+/- IMDQ, 5 mg/mL) were dispersed in water supplemented with 0.1% (v/v) ammonia and sonicated for 1 h. Then, the nanogel solution (5 mg/mL, 38 µL, 15.2 nmol azide) was added to DBCO-modified ovalbumin (0.8 mg/mL, 37.5 µL, 0.7 nmol) and the mixture was filled up to 100 µL with sterile PBS to adjust concentrations. The mixture was incubated at 4°C for 48 h. To obtain a simple mixture of antigen and nanogel for comparative studies, nanogels and fluorescent labeled ovalbumin without DBCO-modification were mixed and incubated. Based on this procedure several sample compositions were prepared regarding different combinations of covalent or admixed IMDQ- and antigen-addition. After incubation samples were analyzed by SDS-PAGE, UV-Vis and fluorescence spectroscopy, DLS and Zeta-potential measurements.

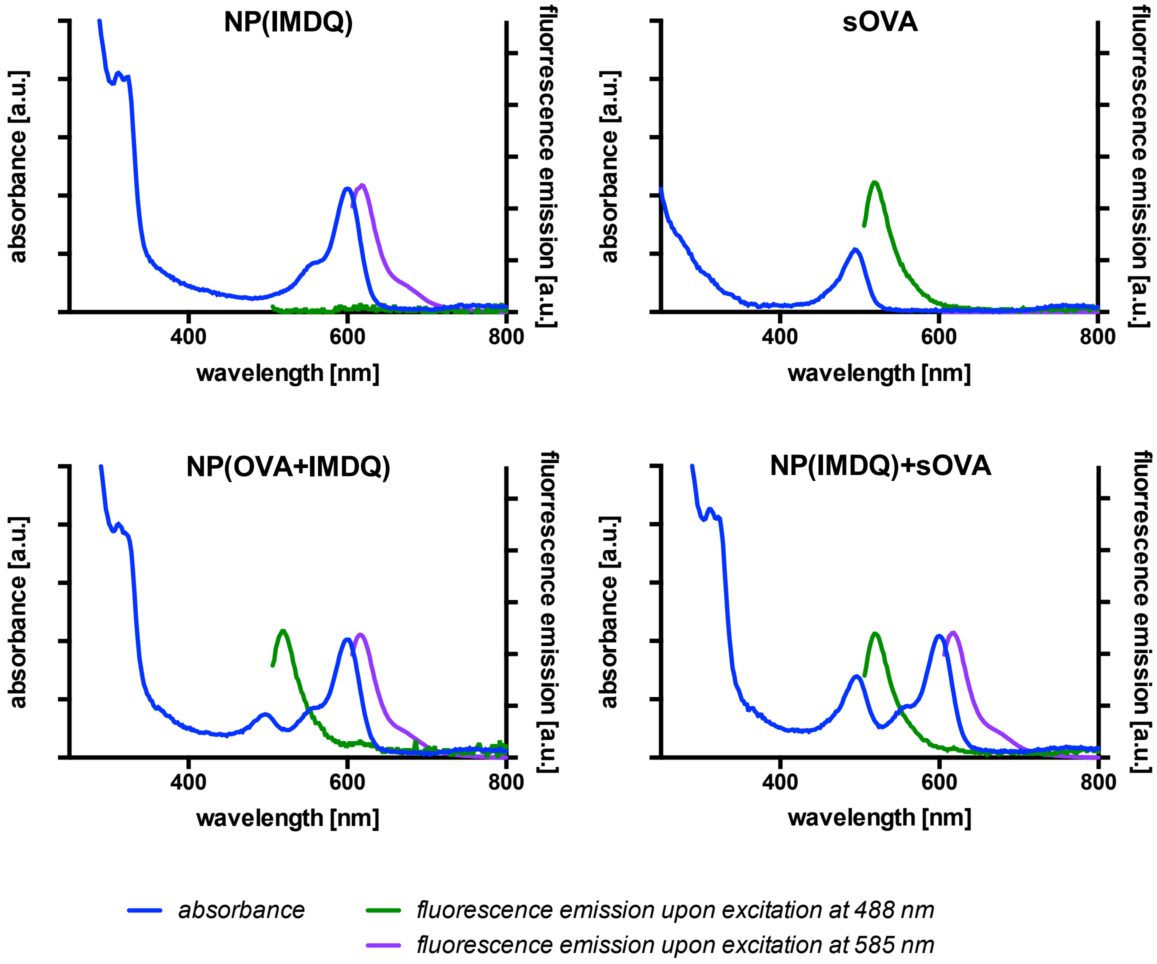

Figure S21: UV-Vis and fluorescence spectra of IMDQ-loaded nanogels, sOVA and both components either separate or covalently attached at 0.4 mg/mL of equivalent nanogel concentration. Note that the fluorescent dyes can be addressed individually from each other.

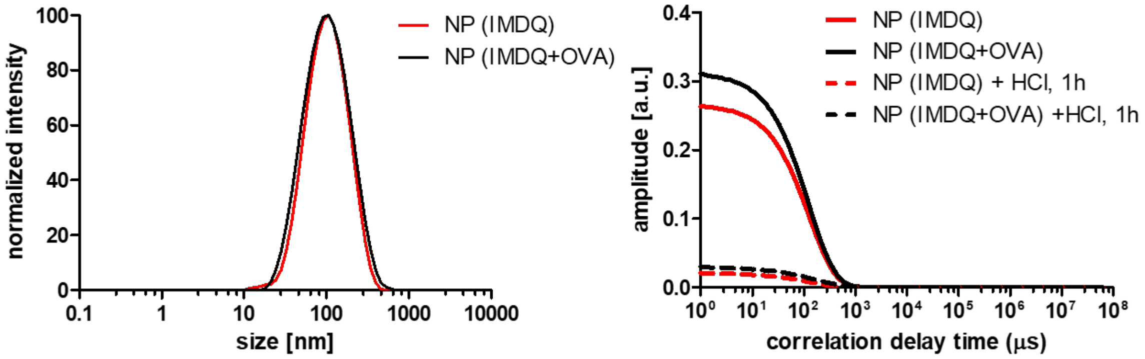

Figure S22: DLS characterization of IMDQ-loaded nanogels with or without covalently attached ovalbumin. After addition of 3 v% 1M HCl correlation strongly decreases due to nanogel degradation into single polymer chains.

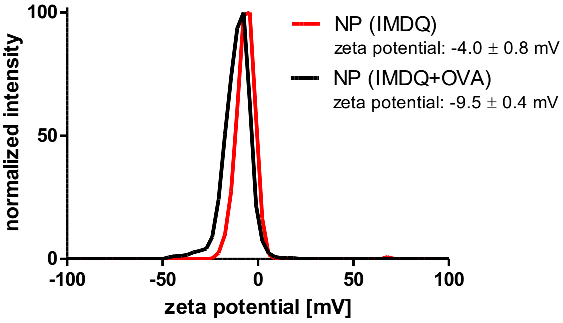

Figure S23: Zeta potential measurements of IMDQ-loaded nanogels with or without covalently attached ovalbumin. Measurements were performed at 0.25 mg/mL in 1 mM KCl.

SDS-PAGE

Reaction monitoring of SPAAC conjugation was performed by SDS-PAGE. SDS-PAGE was performed with 15% polyacrylamide separation gel and 5% polyacrylamide loading gel using the Mini-PROTEAN Tetra Cell from Bio-Rad. Samples were prepared by mixing the corresponding volume of 3 μg protein with 4x reducing Laemmli sample buffer before loading. For molecular weight comparisons Novex™ Sharp Pre-Stained Protein Standard was used. Gel electrophoresis was performed for 55 min at 160 mV. Gel image were obtained after excitation with a UV lamp and after staining with Coomassie Blue with smart phone camera.

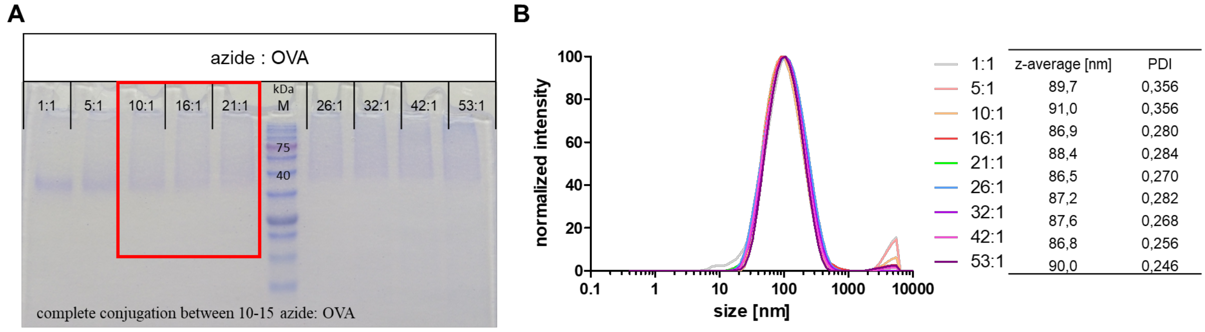

Figure S24: Influence of azide-protein ratio on SPAAC reaction and nanogel size. (A) SDS-PAGE analysis of conjugation of DBCO-ovalbumin to azide-nanogels. (B) DLS intensity size distribution of resulting nanogels. Increasing excess of azide-nanogels does not lead to size changes.

FCS measurements

For FCS measurements nanogels were purified from unbound Texas Red dye prior to conjugate preparation. Hence, 10 mg of each nanogel type (+/- IDMQ) were dissolved in 2 mL of a mixture (1:1) of methanol and water supplemented with 0.1% (v/v) ammonia and sonicated for 1 h. Unbound, free dye was removed by spin-filtration (MWCO 100000 g/mol) of nanogels with the water-methanol mixture. The procedure was repeated until complete disappearance of Texas Red dye-derived absorbance maximum (588 nm) monitored by UV-Vis measurements. Samples were lyophilized and used for preparation of following formulations: NP(IMDQ), NP(IMDQ+OVA), NP(IMDQ) + sOVA. Nanogels were re-dispersed in water supplemented with 0.1% (v/v) ammonia and sonicated for 1 h. Preparation of the different nanogel-ovalbumin compositions was performed as already described. All samples were incubated for two days at 4°C. To investigate degradation behavior hydrochloric acid (3% (v/v), 1 M) was added to one part of each sample. Usually for each FCS measurements 1µL of each sample was diluted with 500 µL PBS in an eight-well polystyrene, chambered cover glass (Laboratory-Tek, Nalge Nunc International). For excitation of the Alexa 488 and TxRed labeled species an argon ion laser (488 nm) and a He/Ne-laser (543 nm) were used simultaneously. The excitation light was focused into the sample by a high numerical aperture water immersion objective (C-Apochromat 40x/1.2 W, Carl Zeiss, Jena, Germany). The fluorescence was collected with the same objective and after passing through a confocal pinhole, directed to a spectral detection unit (Quasar, Carl Zeiss). In this unit emission was spectrally separated by a grating element on a 32-channel array of GaAsP detectors operating in a single photon counting mode. The emission of Alexa 488 was detected in the spectral range 500- 517 nm and that of TxRed in the range 624-696 nm. These arrangements resulted in the formation of two overlapping confocal observation volumes V_b and V_r that superimpose to a common observation volume V_{br}. The sizes of the observation volumes were obtained by performing calibration experiments using Alexa 488 and TxRed as reference dyes with known diffusion coefficients. For each solution, a series of twenty measurements with a total duration of 3 min were performed. The obtained experimental auto- and cross-correlation curves were fitted with the theoretical model function for an ensemble of either 1 or 2 types of freely diffusing fluorescence species for $G(\tau) = 1 + \left[1 + \frac{f_T}{1 - f_T} \cdot e^{-\tau/\tau_T}\right] \cdot \frac{1}{N} \sum_{i=1}^{m} \frac{f_i}{\left[1 + \frac{\tau}{\tau_{D,i}}\right] \cdot \sqrt{1 + \frac{\tau}{S^2 \cdot \tau_{D,i}}}}$ (with m = 1 or 2).[6] The fits yielded the diffusion times of the fluorescent species from which the respective diffusion coefficients were evaluated by $\tau_{D,i} = \frac{\omega^2}{4D_i}$. Finally, the hydrodynamic radii were calculated (assuming spherical particles) by using the Stokes-Einstein relation $R_{h,i} = \frac{k_B T}{6 \cdot \pi \cdot \eta \cdot D_i}$.

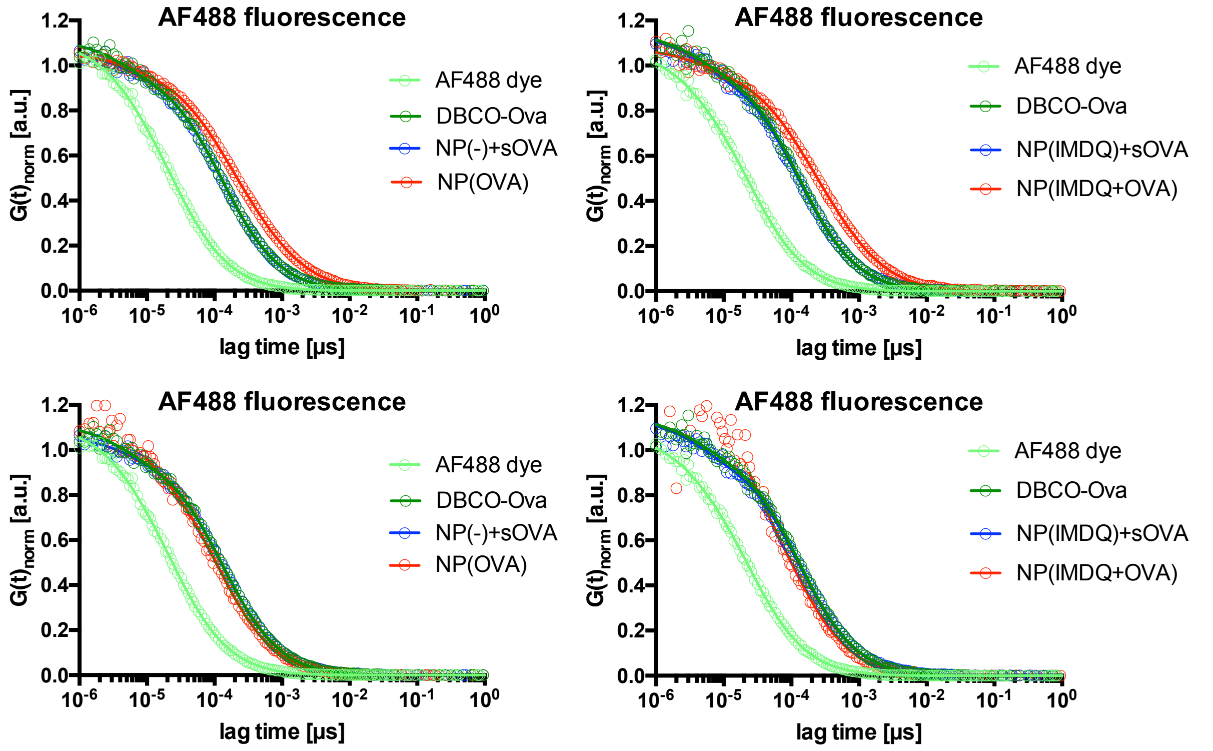

Figure S25: Alexa Fluor 488-based FCS autocorrelation derived from the fluorescently labeled ovalbumin mixed or covalently conjugated to either empty nanogels (left) or IMDQ-loaded nanogels (right) before (top row) or after (bottom row) acidification with 3vol% 1M HCl. Note that the larger correlation times for NP(OVA) and NP(IMDQ+OVA) corresponding to ovalbumin conjugated to nanogels disappear upon acid-triggered particle degradation.

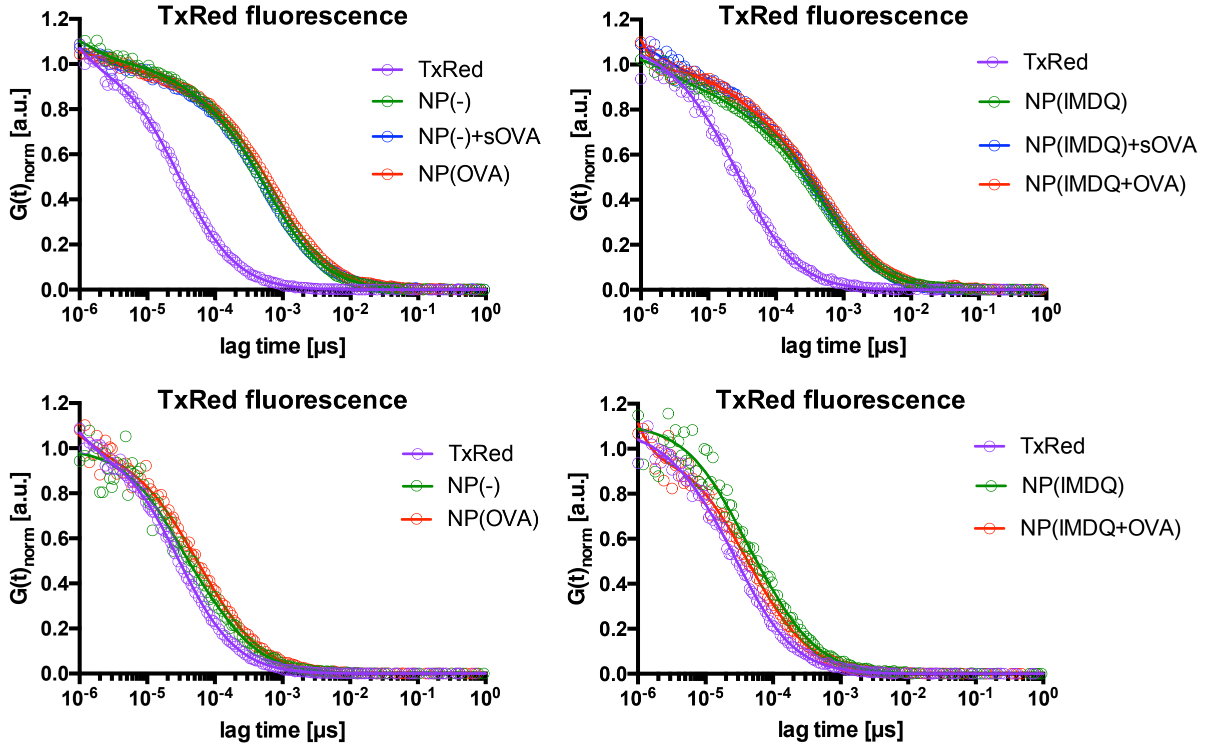

Figure S26: Texas Red-based FCS autocorrelation derived from the fluorescently labeled empty (left) or IMDQ-loaded nanogels (right) mixed or covalently conjugated with ovalbumin before (top row) or after (bottom row) acidification with 3vol% 1M HCl. Note that the larger correlation times for NP(-), NP(IMDQ), NP(OVA) and NP(OVQ+IMDQ) corresponding to intact nanogels disappear upon acid-triggered particle degradation.

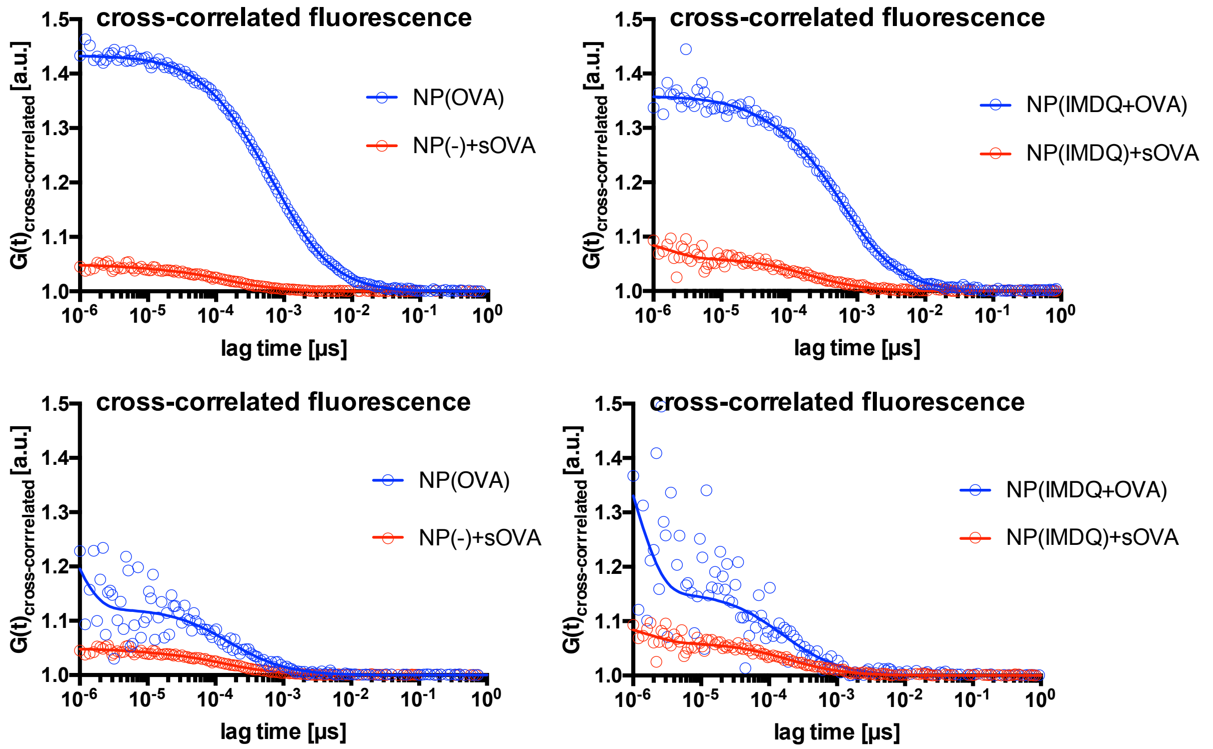

Figure S27: Alexa Fluor 488 and Texas Red derived fluorescent cross-correlation of empty (left) or IMDQ-loaded nanogels (right) mixed or covalently conjugated with ovalbumin before (top row) or after (bottom row) acidification with 3vol% 1M HCl. Note that the cross-correlations for NP(OVA) and NP(IMDQ+OVA) corresponding to ovalbumin conjugated to the nanogel disappear upon acid-triggered particle degradation.

Table S1: Results of Alexa Fluor 488-based FCS measurements derived from the fluorescently labeled ovalbumin mixed or covalently conjugated to either empty or IMDQ-loaded nanogels.

**Hydrodynamic diameter determined by Alexa Fluor 488 derived FCS**<br>*(correlation times and % from one- or two-component fitting)*				
**Alexa Fluor 488**	1.1 nm<br>*τ_1 = 23.1 µs (100%)*			
**OVA**	6.1 nm<br>*τ_1 = 125.6 µs (100%)*			
**OVA-DBCO**	6.4 nm<br>*τ_1 = 130.5 µs (100%)*			
**NP(-)+sOVA**	6.2 nm<br>*τ_1 = 127.9 µs (100%, unbound OVA)*		**NP(IMDQ)+sOVA**	6.3 nm<br>*τ_1 = 130.4 µs (100%, unbound OVA)*
**NP(OVA)**	32.7 nm<br>*τ_1 = 671.2 µs (34%)*<br>*τ_2 = 130.5 µs (66%, free OVA)*		**NP(IMDQ+OVA)**	29.9 nm<br>*τ_1 = 620.0 µs (43%)*<br>*τ_2 = 130.5 µs (57%, free OVA)*
**NP(OVA) acidified**	5.3 nm<br>*τ_1 = 107.7 µs (100%)*		**NP(IMDQ+OVA) acidified**	4.9 nm<br>*τ_1 = 102.3 µs (100%)*

Table S2: Results Texas Red-based FCS measurements derived from the fluorescently labeled empty or IMDQ-loaded nanogels mixed or covalently conjugated with ovalbumin.

**Hydrodynamic diameter determined by Texas Red derived FCS**<br>*(correlation times and % from one- or two-component fitting)*				
**Texas Red cadaverine**	1.4 nm<br>*τ_1 = 29.5 µs (100%)*			
**NP(-)**	30.0 nm<br>*τ_1 = 614.3 µs (84%)*<br>*τ_2 = 29.5 µs (16%, free dye)*		**NP (IMDQ)**	26.8 nm<br>*τ_1 = 558.4 µs (75%)*<br>*τ_2 = 29.5 µs (25%, free dye)*
**NP(-) acidified**	3.6 nm<br>*τ_1 = 74.8 µs (99%)*<br>*τ_2 = 29.5 µs (<1%, free dye)*		**NP(IMDQ) acidified**	3.6 nm<br>*τ_1 = 74.3 µs (99%)*<br>*τ_2 = 29.5 µs (<1%, free dye)*
**NP(-)+sOVA**	30.4 nm<br>*τ_1 = 623.8 µs (85%)*<br>*τ_2 = 29.5 µs (15%, free dye)*		**NP(IMDQ)+sOVA**	25.2 nm<br>*τ_1 = 519.7 µs (77%)*<br>*τ_2 = 29.5 µs (23%, free dye)*
**NP(OVA)**	36.7 nm<br>*τ_1 = 752.5 µs (87%)*<br>*τ_2 = 29.5 µs (13%, free dye)*		**NP(IMDQ+OVA)**	29.1 nm<br>*τ_1 = 605.0 µs (75%)*<br>*τ_2 = 29.5 µs (25%, free dye)*
**NP(OVA) acidified**	5.4 nm<br>*τ_1 = 110.6 µs (50%)*<br>*τ_2 = 29.5 µs (50%, free dye)*		**NP(IMDQ+OVA) acidified**	4.9 nm<br>*τ_1 = 101.2 µs (32%)*<br>*τ_2 = 29.5 µs (%, free dye*

Reproducibility of nanogel fabrication

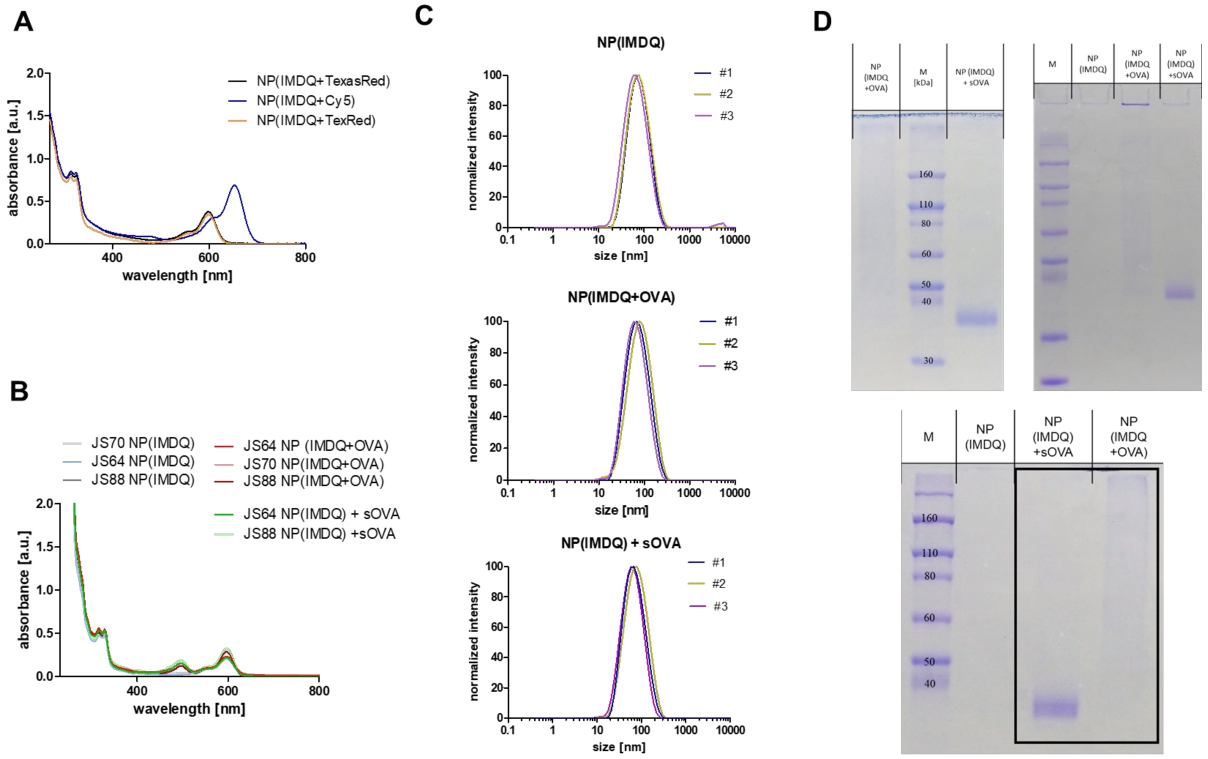

Figure S28: Representative data of various nanogel preparations demonstrating a reproducible fabrication process. (A) UV-Vis spectra of 3 batches of IMDQ-loaded nanogels showing similar drug-loading and dye-labeling (TexasRed). (B) UV-Vis spectra of nanogel samples with or without addition of ovalbumin used for in vitro an in vivo experiments. Same concentrations of IMDQ and OVA are observable. (C) DLS intensity size distribution plots of three nanogel preparations showing similar sizes between the preparations independent of samples type. (D) SDS-PAGE analyses of three different SPAAC conjugations of azide-nanogels and ovalbumin revealing complete conjugation of protein to the carrier system.

RAW-Blue macrophage TLR reporter assay

IMDQ-triggered TLR receptor stimulation followed by NF-kB/AP-1 activation (detectable by secretion of embryonic alkaline phosphatase) was performed on RAW-Blue macrophages as proposed by the manufacturer (InvivoGen) and in analogy to earlier reports.[7,8] Into a 96-well plate RAW-Blue cells were seeded at 90000 cells/well in 180 µL cell culture medium. After cell adhesion overnight each well was incubated with 20 µL of IMDQ-containing samples at given concentrations for 16 h. Then, 50 µL of the supernatant of each well was transferred into a new 96-well plate and tested for secreted embryonic alkaline phosphatase using the QUANTI-Blue assay (InvivoGen). 150 µL QUANTI-Blue solution was added to each well and samples were incubated at 37°C for 30 min. Levels of secreted embryonic alkaline phosphatase were determined by measuring optical density at 620 nm with a microplate reader. Increased TLR activity was determined by an increase in optical density relative to a negative control treated with PBS. IMDQ served as positive control and experiments were performed in quadruplicate (n = 4).

RAW-Blue macrophage cell viability by MTT assay

25 µL of 3-(4,5-dimethylthiazol-2-yl)-2,5-diphenyltetrazolium bromide (MTT, 2 mg/mL PBS) was added to each well containing RAW-Blue macrophages which were treated with IMDQ-samples at given concentrations. After incubation at 37°C for 1 h, 100 µL of 10% SDS (m/v)/0.01 M HCl were added to each well to dissolve formed formazan crystals followed by incubation overnight at 37°C. Cell viability was determined in relation to positive (PBS blank, 100% viability) and negative (10% DMSO, 0% viability) control samples based on absorbance measurements at 570 nm. Experiments were performed in quadruplicate (n = 4).

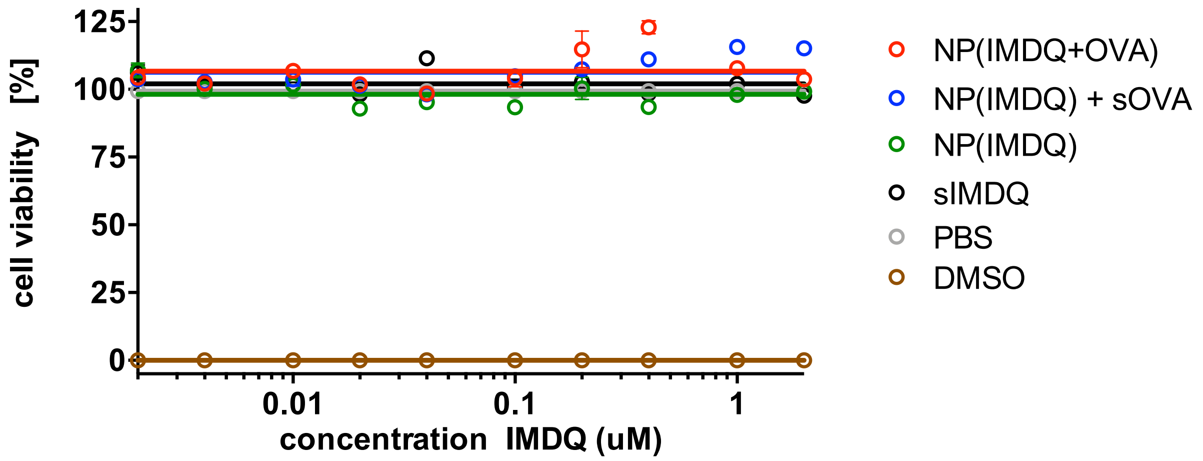

Figure S29: In vitro evaluation of IMDQ-loaded nanogels in comparison to soluble TLR agonist. Nanogels show no negative effect on cell viability.

Flow cytometry of RAW-Blue macrophages

RAW-Blue macrophages were seeded into 24-well plates in a density of 250000 cells/well in 980 µL cell culture medium and incubated overnight to allow cell adhesion. To each well 20 µL nanogel sample (resulting in a nanogel concentration of 40 µg/mL) was added and cells were incubated at 37°C. After 16 h of incubation, cell culture medium was aspirated and cells were washed with 1 mL PBS followed by an incubation with 500 µL Cell Dissociation Buffer for 20 min. Then, cells were detached and cell suspensions were transferred into Eppendorf tubes and centrifuged immediately (300 g, 10 min, 5°C). The supernatant was aspirated and cell pellets were re-suspended in 500 µL PBS and put on ice. All samples were run in triplicate (n = 3). Flow cytometric analysis was performed on an Attune NxT flow cytometer (Thermo Fisher) and the resulting data processed by FlowJo software following the displayed gating strategy.

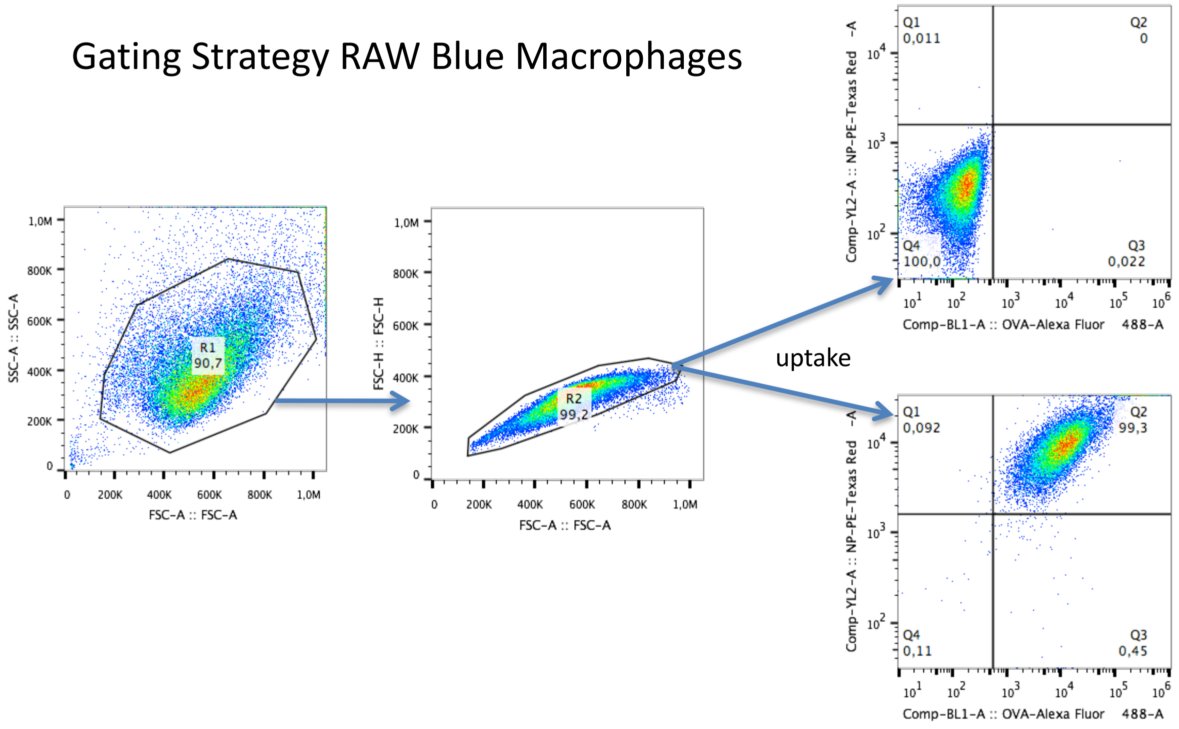

Figure S30: Gating strategy for flow cytometry analysis of the uptake of different IMDQ-nanogel formulations and ovalbumin by RAW-Blue macrophages of (n = 3).

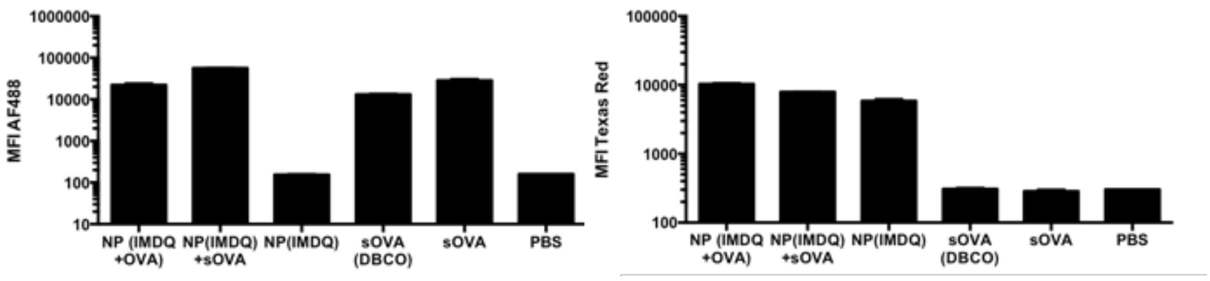

Figure S31: Results of mean fluorescence intensity (MFI) from the flow cytometry analysis of the uptake different IMDQ-nanogel formulations and ovalbumin by RAW-Blue macrophages (n = 3).

Confocal fluorescence microscopy of RAW-Blue macrophages and DC2.4

RAW-Blue macrophages (50,000 cells/well, suspended in 0.18 mL of the culture medium) were seeded into Ibidi µ-slide eight-well confocal microscopy chambers and left to adhere overnight. Afterwards, cells were incubated with 20 µL nanogel sample with soluble or covalently attached OVA sample (yielding a total nanogel concentration of 40 µg/mL) for 16 h. Then, culture medium was aspirated and cells were washed three times with PBS. Next, 200 µL of 4% paraformaldehyde was added and allowed to fixate for 15 min at 37 °C. Subsequently, cells were washed again three times with PBS and their nuclei were stained with 200 µL of 4′,6-diamidino-2-phenylindole (DAPI) (50 µg/mL in PBS) for 15 min at 37 °C. Finally, cells were washed three times with PBS and stored under an aqueous mounting medium. Fluorescence confocal laser scanning microscopy images were recorded on a Leica SP5 confocal microscope system with a 63× immersion objective. All images were finally processed by the ImageJ software package.

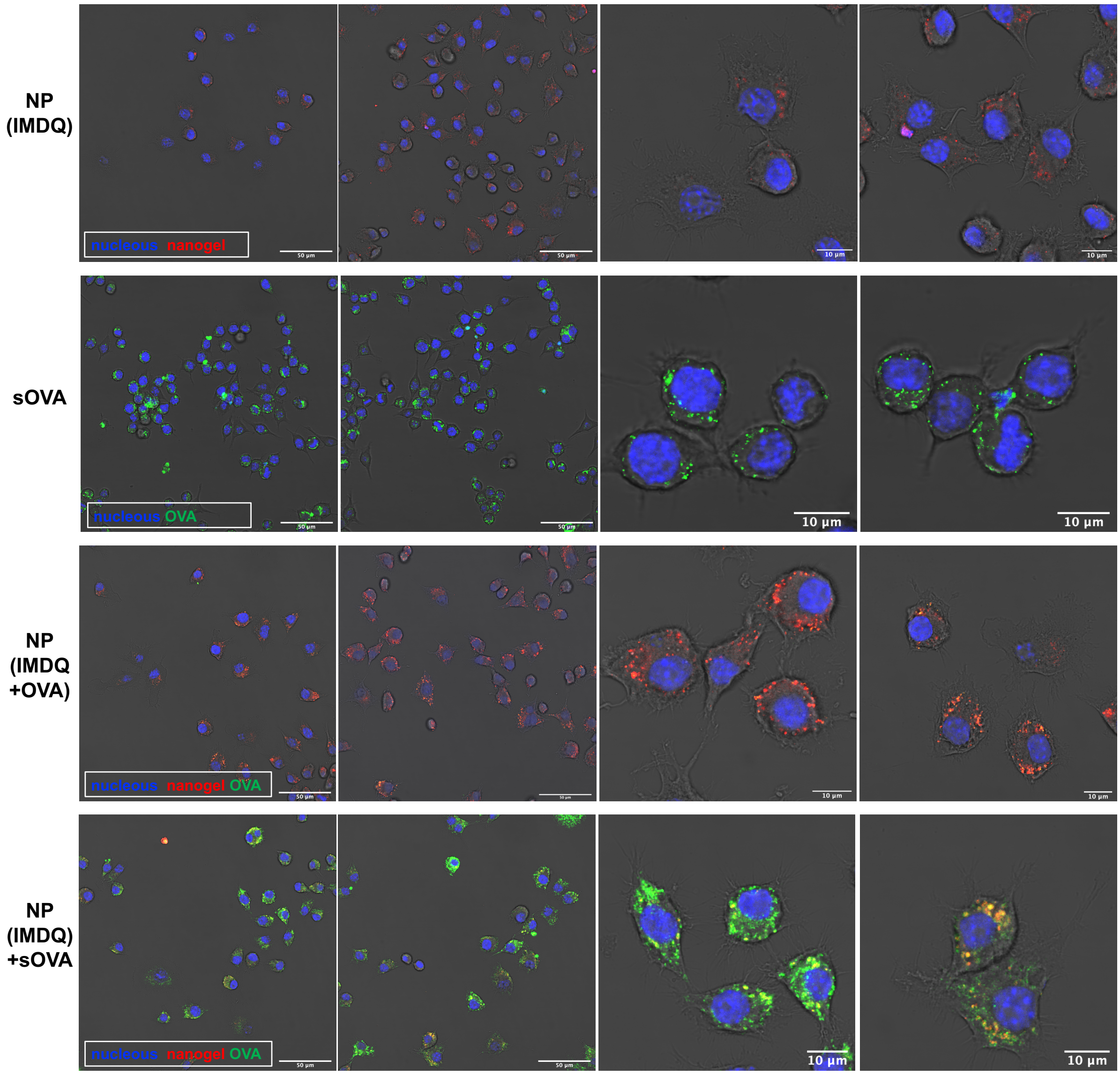

Figure S32: Fluorescence confocal microscopy image of RAW Blue macrophages incubated with IMDQ- and OVA-loaded nanogels NP(IMDQ+OVA) as well as their corresponding controls NP(IMDQ), sOVA and .NP(IMDQ)+sOVA. Nanogels could be identified by their Texas Reed label (red), while OVA could be visualized by the Alexa Fluor 488 label (green).

Under similar conditions DC2.4 dendritic cells were also incubated with soluble or covalently attached OVA samples and imaged by confocal microscopy. Again, a strong co-localization was found for OVA covalently bound to the nanogel carrier.

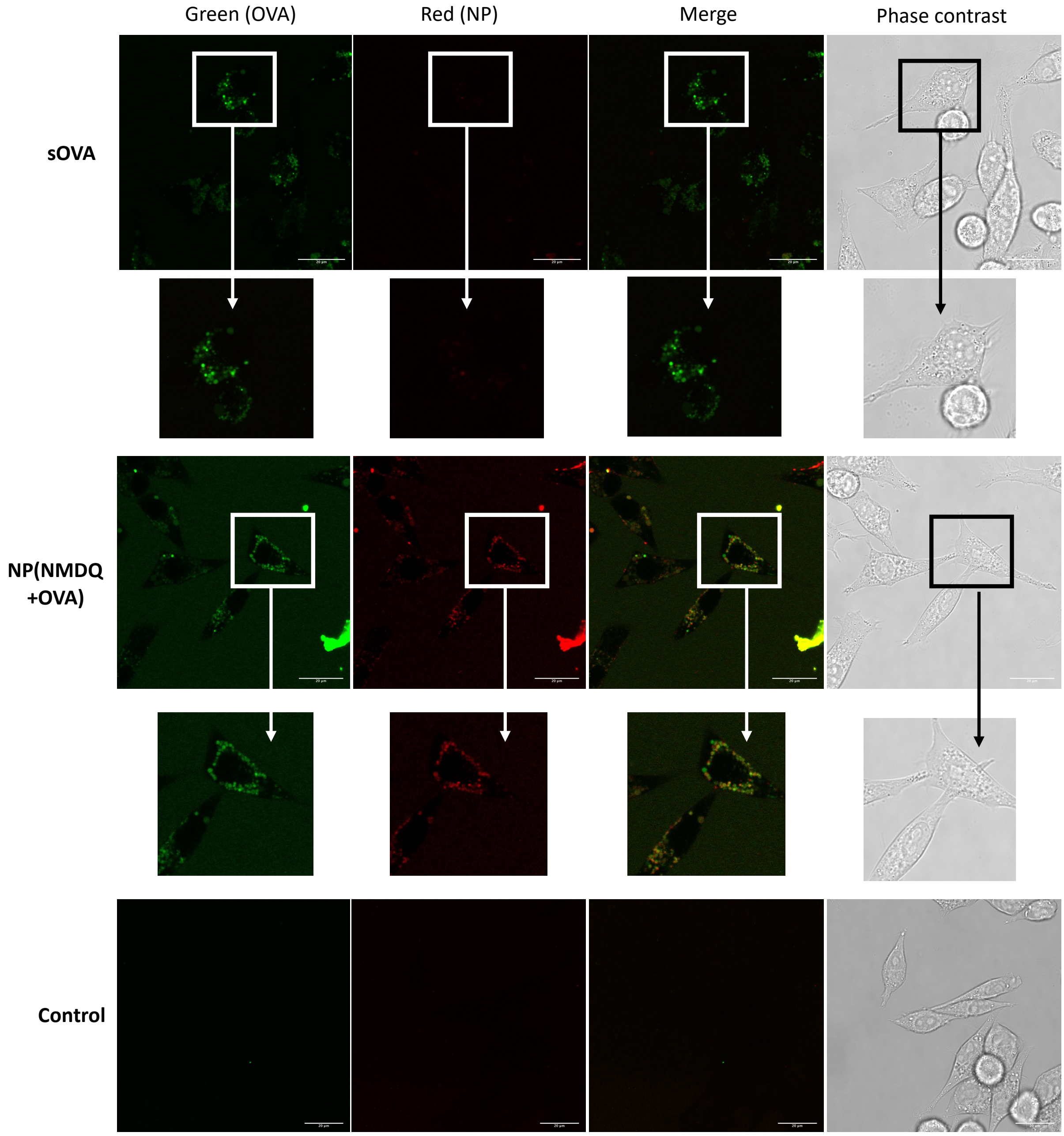

Figure S33: Fluorescence confocal microscopy image of DC2.4 dendritic cells incubated with sOVA and IMDQ- and OVA-loaded nanogels NP(IMDQ+OVA). Nanogels could be identified by their Texas Red label (red), while OVA could be visualized by the Alexa Fluor 488 label (green).

In vitro read-out in spleen cells

Spleen cells were derived from C57BL/6 mice, and erythrocytes were lysed using a hypotonic lysis buffer (155 mM NH_4Cl, 10 mM $KHCO_3$, 100 μM EDTA, pH 7.4). Spleen cells were resuspended in IMDM-based culture medium ($4x10^6$/mL). Aliquots were transferred into sterile FACS tubes (each 500 µl). The various NP formulations (1 µg/mL) were applied either directly or after preincubation with the same volume of native mouse serum (30 min, 37 °C) to exclude the influence of a potentially formed protein corona. On the next day, samples were washed, and subjected to flow cytometric analysis.

Flow cytometry of spleen cells

Again, in order to prevent unspecific binding of subsequently added antibodies, samples were incubated first with a rat anti-mouse CD16/CD32 (Fc-block) antibody (15 min, 4 °C). Next, spleen cells were incubated for 20 min at 4 °C with specific antibodies for CD11b (SB600), CD11c (PE-Cy7), CD19 (SB702), Ly6G (PE), MHC-II(APC) and CD86 (FITC – note that its labeling was way much stronger that the weak fluorescence derived from the AF488-labeled OVA, e.g. no difference between NP(IMDQ) and NP(IMDQ+OVA)). All antibodies were obtained from BioLegend (San Diego, CA) or Thermo Fisher (Waltham, MA). After antibody incubation, samples were incubated with fixable viability dye (FVD) eFl450 (Thermo Fisher) to exclude dead cells. Samples left untreated, incubated with only a NP formulation or with a single antibody only served as controls. Washed samples were fixed and subjected to flow cytometric analysis employing an Attune NxT flow cytometer (Thermo Fisher). The resulting data were processed by FlowJo software following the displayed gating strategy. All samples were run in biological triplicate (n = 3) and referenced to the negative control sample (PBS group).

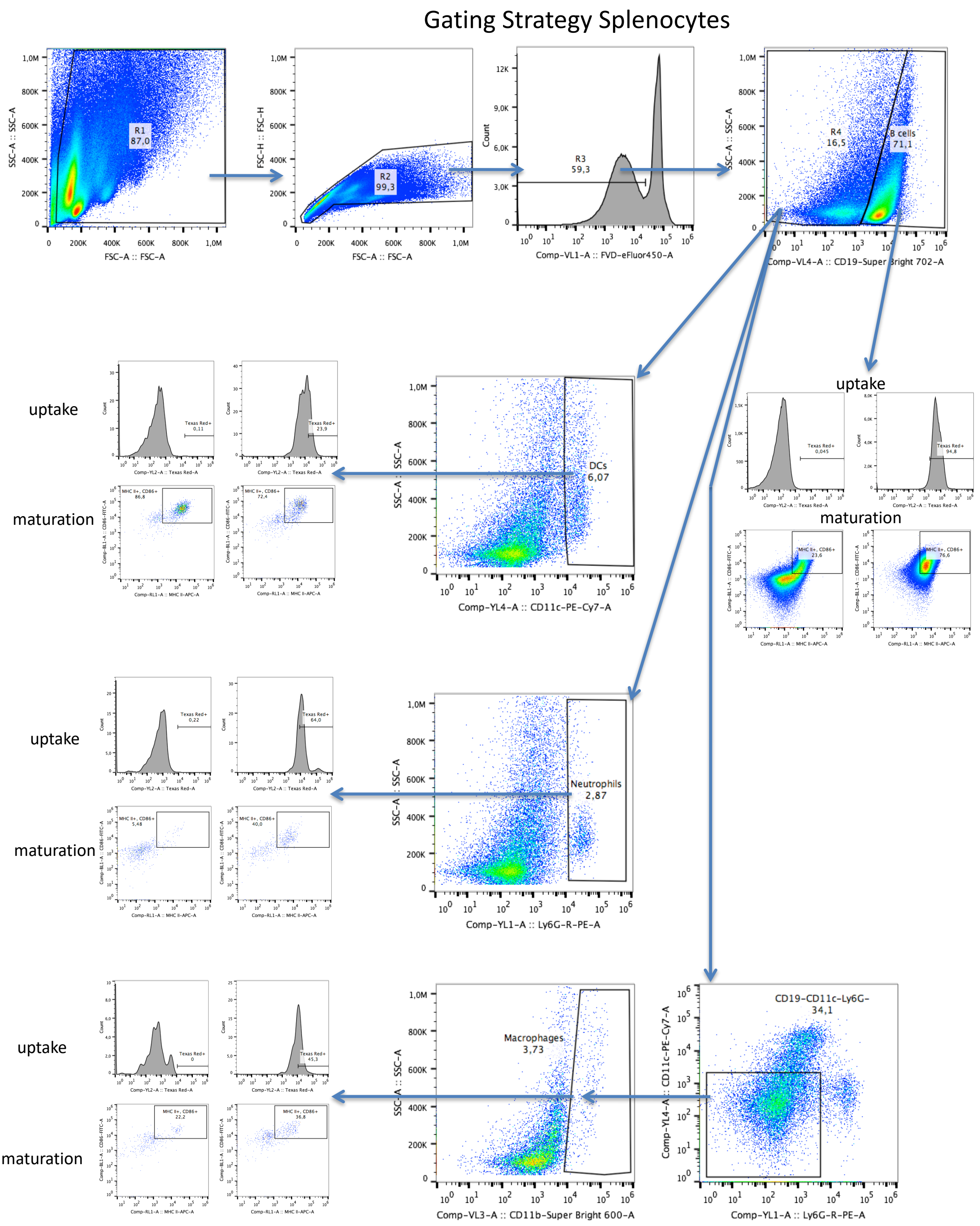

Figure S34: Gating strategy for flow cytometry analysis of spleen cells incubated with IMDQ/OVA nanogel formulations with respect to particle uptake and immune cell maturation.

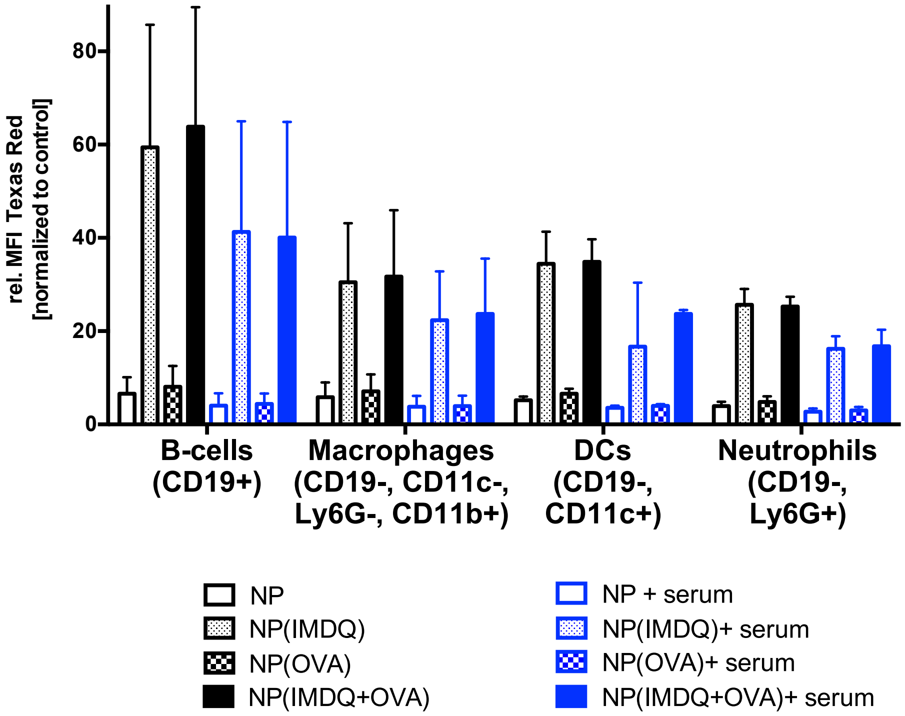

Figure S35: Results of relative mean fluorescence intensity (MFI – in relation to cells treated with PBS9 from the flow cytometry analysis of splenocytes incubated with covalent IMDQ and OVA nanogel formulations. Note that a pre-incubation of nanogels with mouse serum (blue) did not affect the uptake of the nanogels into the different immune cell populations.

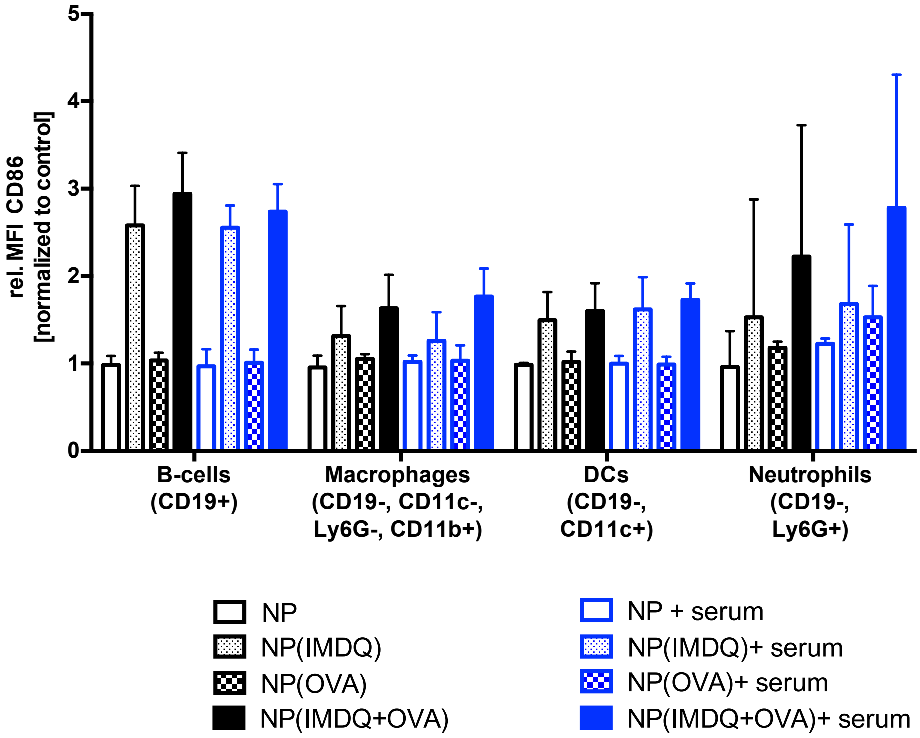

Figure S36: Results of relative mean fluorescence intensity (MFI – in relation to cells treated with PBS) from the flow cytometry analysis of splenocytes incubated with covalent IMDQ and OVA nanogel formulations. Note that a pre-incubation of nanogels with mouse serum did not affect the maturation status (MFI of CD86 expression) of the different immune cell populations after x vivo treatment with nanogels.

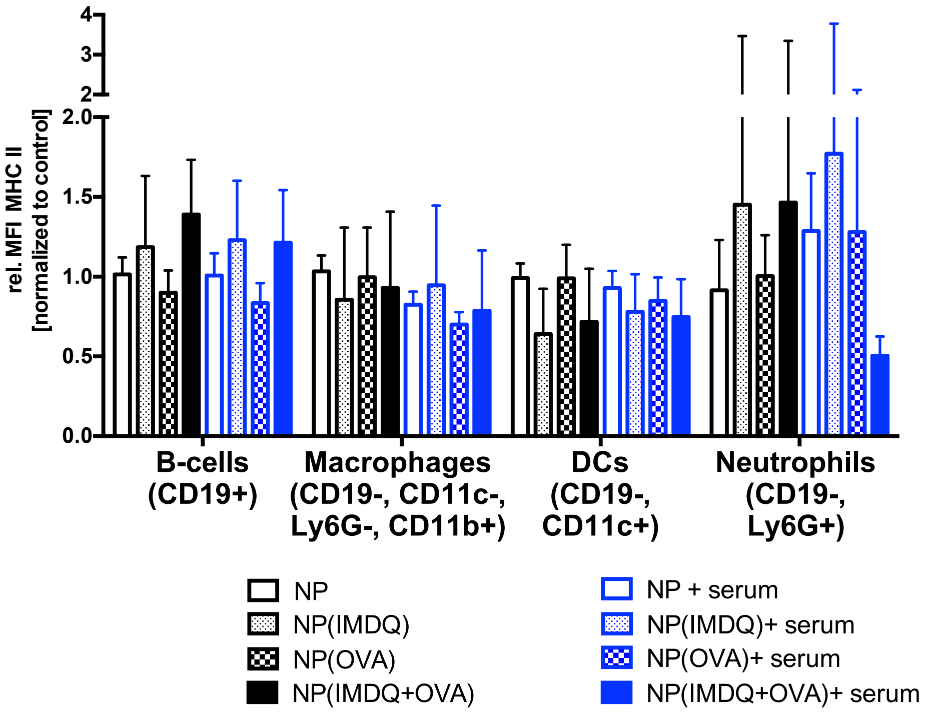

Figure S37: Results of relative mean fluorescence intensity (MFI – in relation to cells treated with PBS) from the flow cytometry analysis of splenocytes incubated with covalent IMDQ and OVA nanogel formulations. Note that the basal expression levels of MHC-II were already quite high and a pre-incubation of nanogels with mouse serum did not alter the maturation status either (MFI of MHC-II expression).

Microscopy analysis of spleen cells

For interaction analyses, a wildtype mouse was sacrificed and the splenocytes were isolated. $1x10^6$ splenocytes were seeded into a well of a 6-well plate and glass bottom dishes (MatTek Corporation, Ashland, United States) and were either treated with 100 µl of NP(IMDQ+OVA) or left untreated. After 4 hours of incubation at 37 °C, the cells were harvested, washed and then stained with CD19-Biotin followed by a secondary staining against CD19-Biotin with Streptavidin-Pacific Blue (obtained from BioLegend (San Diego, CA) and Thermo Fisher (Waltham, MA)), both for 1 hour at room temperature in PBS/1 % FCS. At least, the cells were covered with PBS and analysed on a fluorescence microscope (Axiovert 200 M, Carl Zeiss, Jena), kindly supported by R. Stauber (Department of Otolaryngology, Head and Neck Surgery, University Medical Center Mainz). Brightness and contrast of the images were adapted using the Canvas™ software (ACD Systems, Seattle, United States).

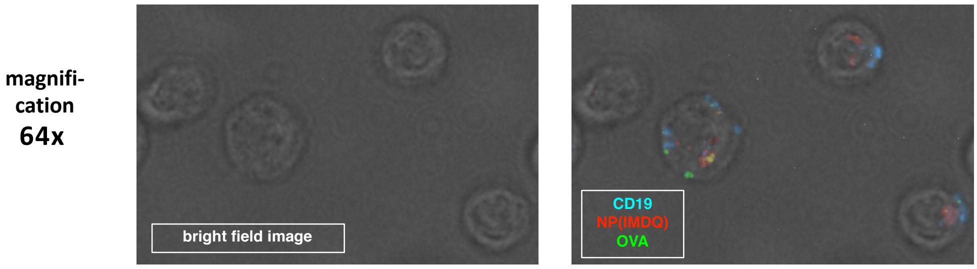

Figure S38: Fluorescence microscopy image of splenocytes incubated with IMDQ- and OVA-loaded nanogels NP(IMDQ+OVA). Nanogels could be identified by their Texas Reed label (red), while OVA could be visualized by the Alexa Fluor 488 label (green). Moreover, B-cells could be identified by antibody staining against CD19.

In vitro read-out in bone marrow-derived dendritic cells (BMDCs)

Bone marrow cells were seeded in 12 well suspension cell culture cluster plates (each $2x10^5$/mL). Culture media was replenished on days 2 and 6. On day 7-8 the different NP formulations were applied (1 µg/mL). On the following day, samples were harvested and subjected to flow cytometric analysis or employed as stimulators in T cell proliferation assays.

Flow cytometry of BMDCs

In order to prevent unspecific binding of subsequently added antibodies, samples were first incubated with a rat anti-mouse CD16/CD32 (Fc-block) antibody (15 min, 4 °C). Specific antibodies for CD11c (conjugated with PE-CY7), MHC-II (APC) and CD86 (eFl450) were applied for 20 min at 4 °C. All antibodies were obtained from BioLegend (San Diego, CA) or Thermo Fisher (Waltham, MA). Samples left untreated, incubated with only a NP formulation or with a single antibody only served as controls. Washed samples were fixed and subjected to flow cytometric analysis employing an Attune NxT flow cytometer (Thermo Fisher) The resulting data were processed by FlowJo software following the displayed gating strategy.

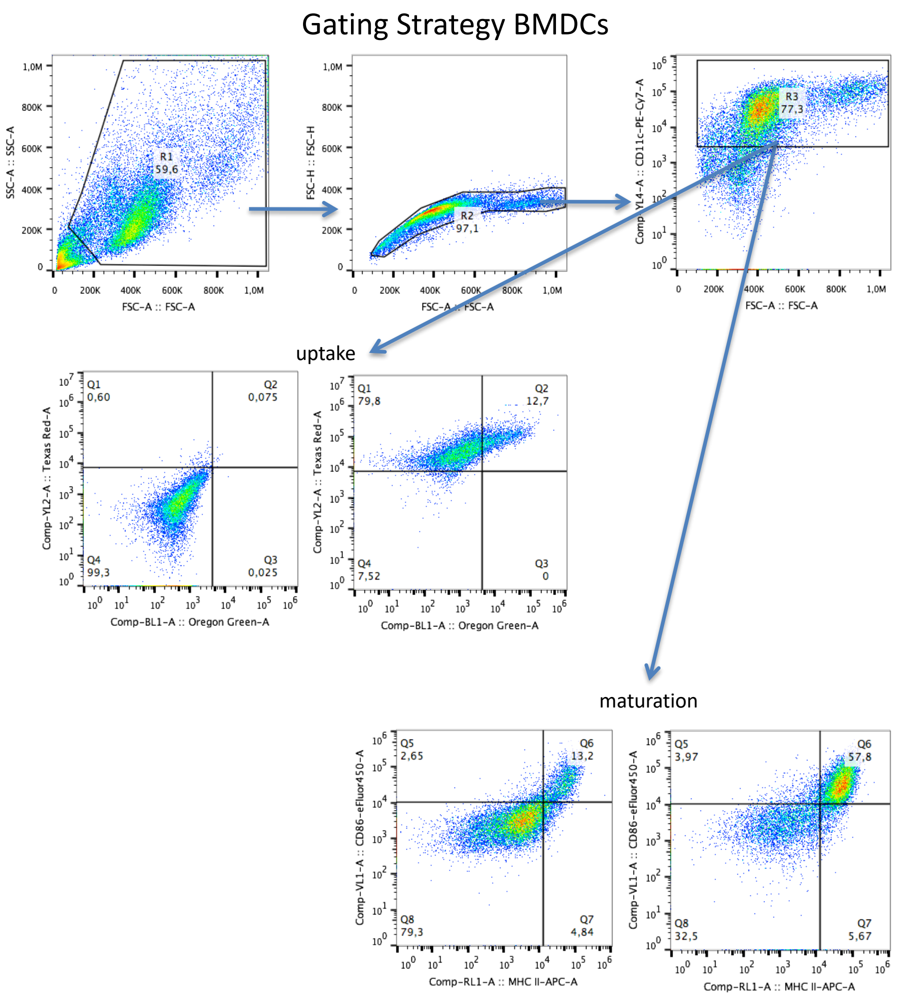

Figure S39: Gating strategy for flow cytometry analysis of uptake of different IMDQ-nanogel formulations and immune cell maturation by BMDCs.

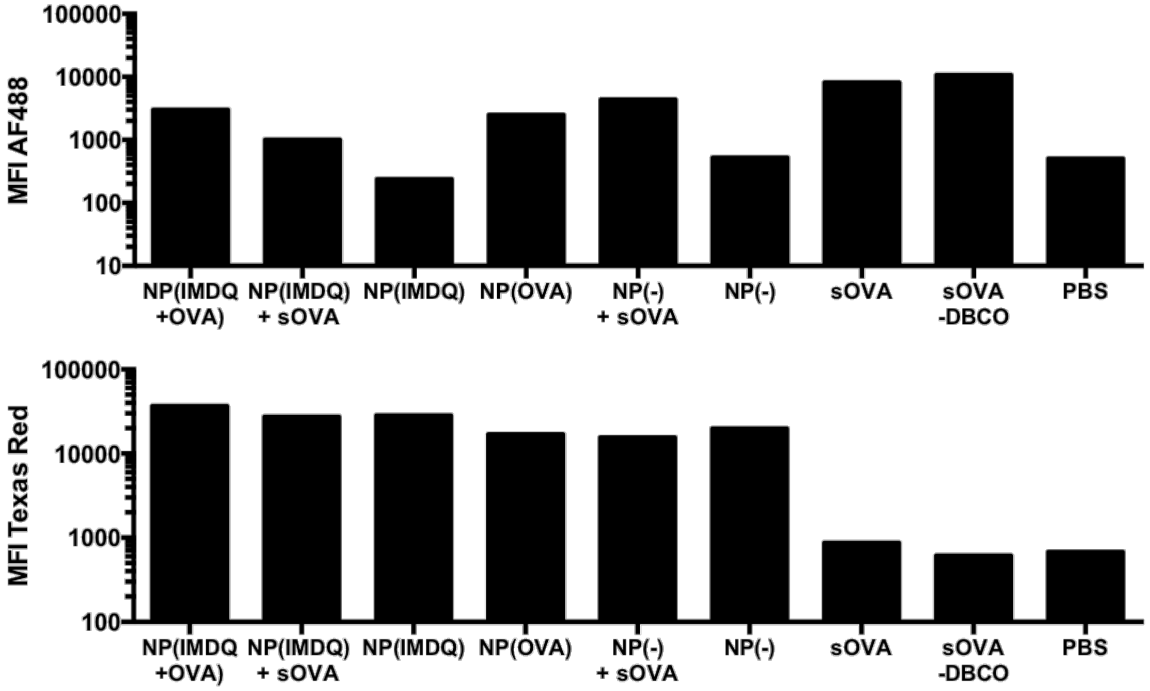

Figure S40: Results of mean fluorescence intensity (MFI) from the flow cytometry analysis of the uptake different IMDQ-nanogel formulations and ovalbumin by BMDCs.

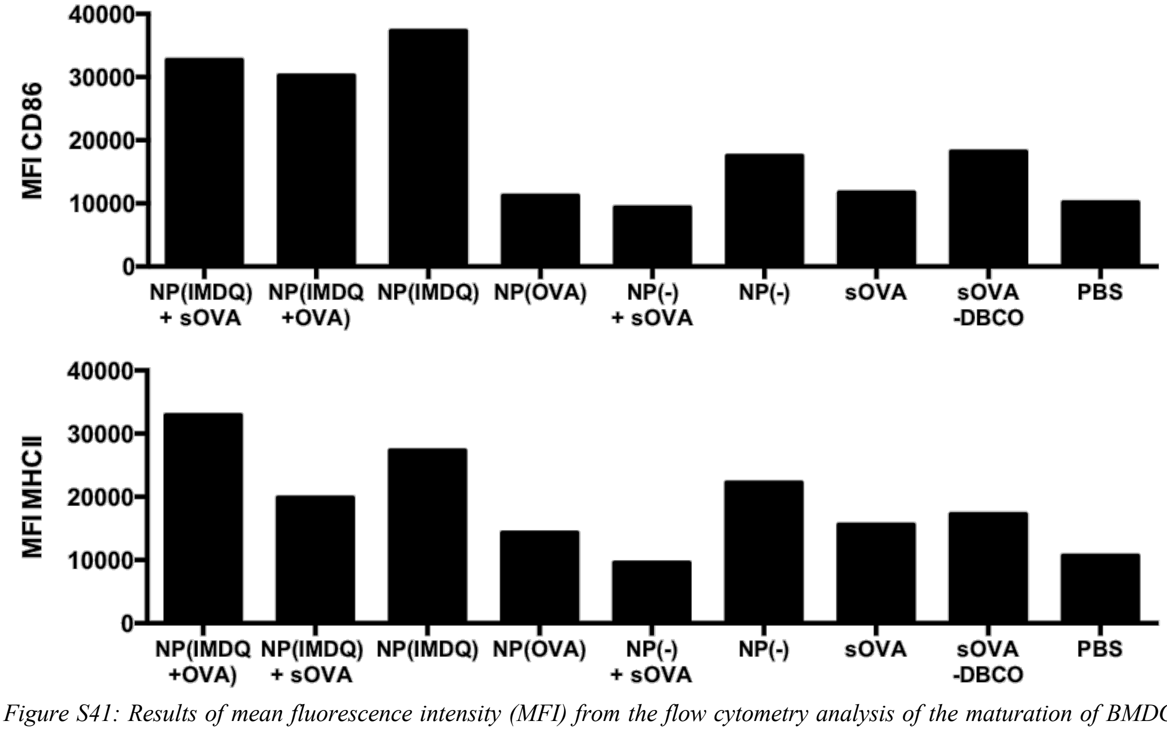

Figure S41: Results of mean fluorescence intensity (MFI) from the flow cytometry analysis of the maturation of BMDCs by different IMDQ-nanogel formulations and ovalbumin.

T-cell proliferation and cytokine assay

Differentially pretreated BMDCs were harvested and reseeded in triplicates into 96-well plates (10^5/mL; 100 µl/well) and were serially diluted. OVA peptide-specific splenic $CD8^+$ T cells from OT-I mice (https://www.ncbi.nlm.nih.gov/pubmed/10762410) and $CD4^+$ T cells from OT-II mice (https://www.ncbi.nlm.nih.gov/pubmed/9553774) were isolated by negative immunomagnetic enrichment (Miltenyi, Bergisch-Gladbach, Germany). Isolated T cells ($5x10^5$/mL; each 100 µl) were added to diluted BMDC. Aliquots of cell culture supernatants were taken on day 1, 2 and 3 and stored at -20 ºC. Then they were analyzed by a cytometric bead array according to the manufacturer (BD, Cytometric Bead Array Soluble Protein Flex Set – beads were measured with a Attune NxT flow cytometer, Thermo Fisher). On day 3, 3H-thymidine (0.5 µCi/well) was added for the last 16-18 h of coculture. Afterwards, cells were harvested onto glass fiber filters, and retained radioactivity was quantified in a microplate scintillation counter (1450 MicroBeta Trilux; Perkin Elmer, Waltham, MA). All samples were run in triplicate (n = 3).

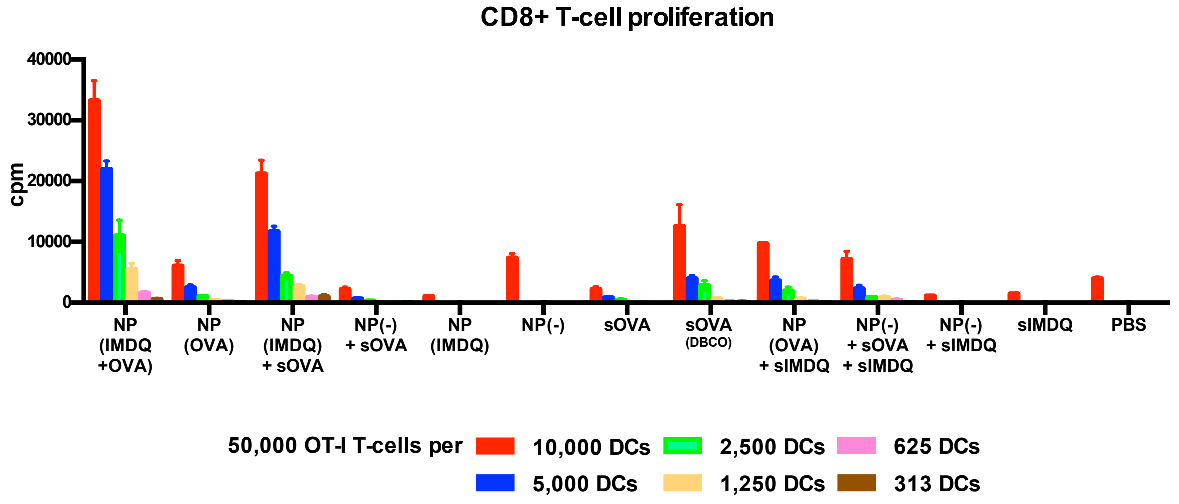

Figure S42: $CD8^+$ specific T-cell proliferation derived from OT-I T cells incubated with serial dilutions of BMDCs pulsed with nanogel samples loaded with IMDQ and/ or OVA as well as respective control samples of nanogels with soluble compounds.

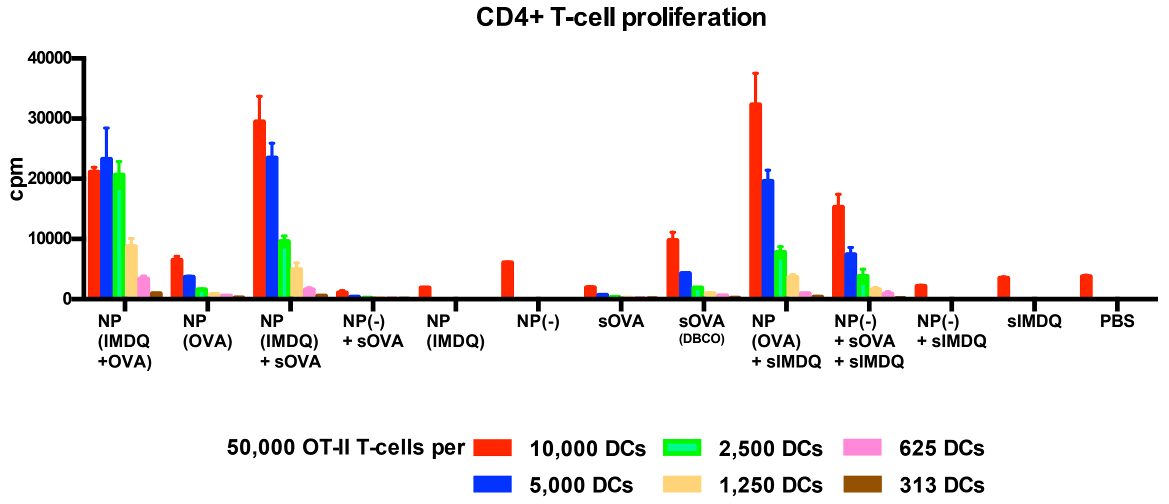

Figure S43: $CD4^+$ specific T-cell proliferation derived from OT-II T cells incubated with serial dilutions of BMDCs pulsed with nanogel samples loaded with IMDQ and/ or OVA as well as respective control samples of nanogels with soluble compounds.

Internalization and inhibition of NP(IMDQ+OVA-FITC) and sOVA by dendritic cells

For internalization experiments, 1 x 10^6 DC2.4 cells were incubated with NP(IMDQ+OVA) at a dilution of 1:150 in the presence of 2 % mouse serum for 30 min at 37°C after 1h starvation in serum-free X-VIVO medium. For inhibition experiments, 300 µg/mL Fucoidan (Sigma) was added. Analysis of the internalized NP by DC2.4 cells was performed using an Amnis ImageStream Mk II flow cytometer (Luminex) at the Core Facility of the Research Center for Immunotherapy (University Medical Center, Johannes Gutenberg-University Mainz). Data were evaluated using the IDEAS software (Luminex). Dead cells were gated out using DAPI (Thermo Fisher Scientific) at a dilution of 1:10,000.

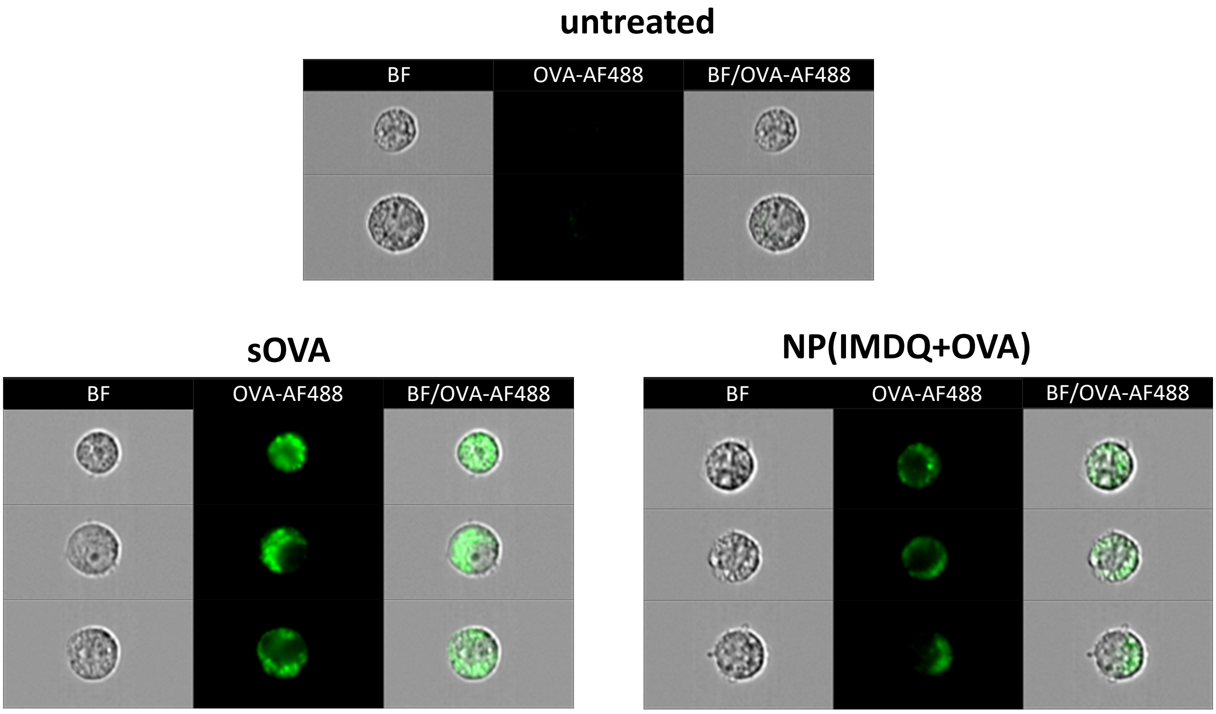

Figure S44: Results of imaging flow cytometry of DCs incubated with sOVA and OVA-conjugated IMDQ-nanogel formulations. ImageStream recorded images of bright field (BF), Alexa Fluor 488 derived OVA fluorescence and overlay of untreated DC2.4 or incubated with sOVA and NP(OVA+IMDQ) (magnification 40x).

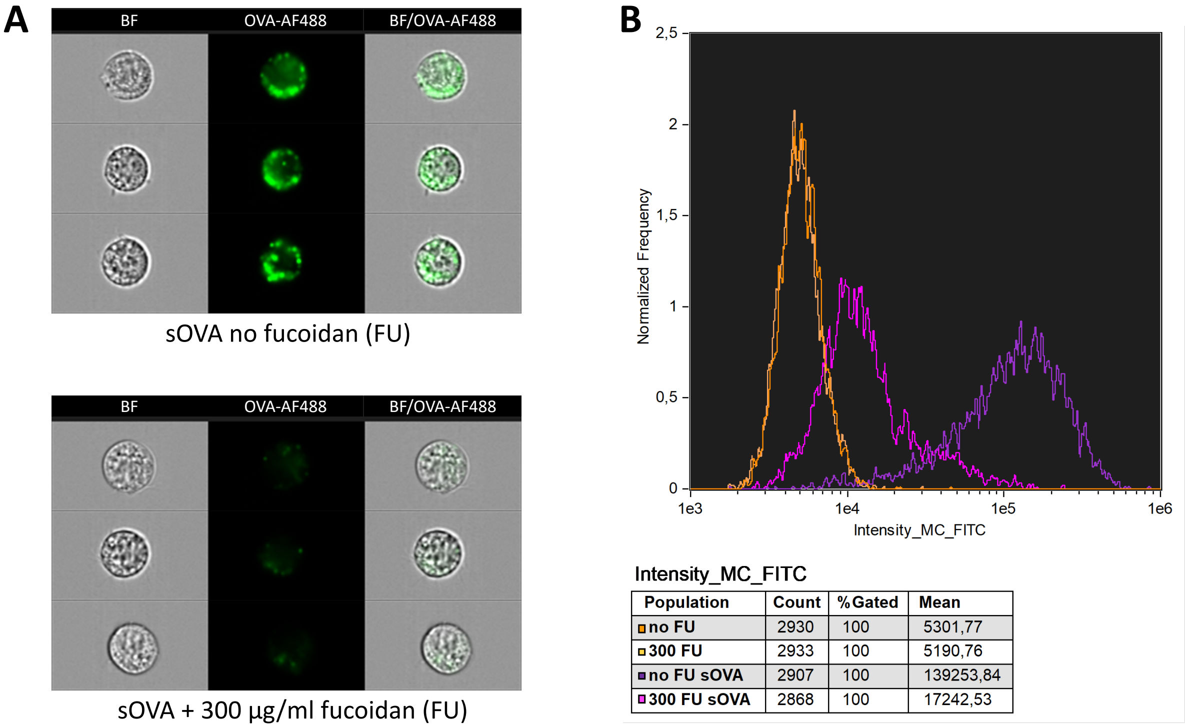

Population	Count	%Gated	Mean
no FU	2930	100	5301,77
300 FU	2933	100	5190,76
no FU sOVA	2907	100	139253,84
300 FU sOVA	2868	100	17242,53

Figure S45: Results of imaging flow cytometry of DCs incubated with sOVA with and without 300 µg/mL fucoidan (FU). (A) ImageStream recorded images of bright field (BF), Alexa Fluor 488 derived OVA fluorescence and overlay (magnification 40x). (B) Derived histogram for the samples with and without 300 µg/mL fucoidan (FU).

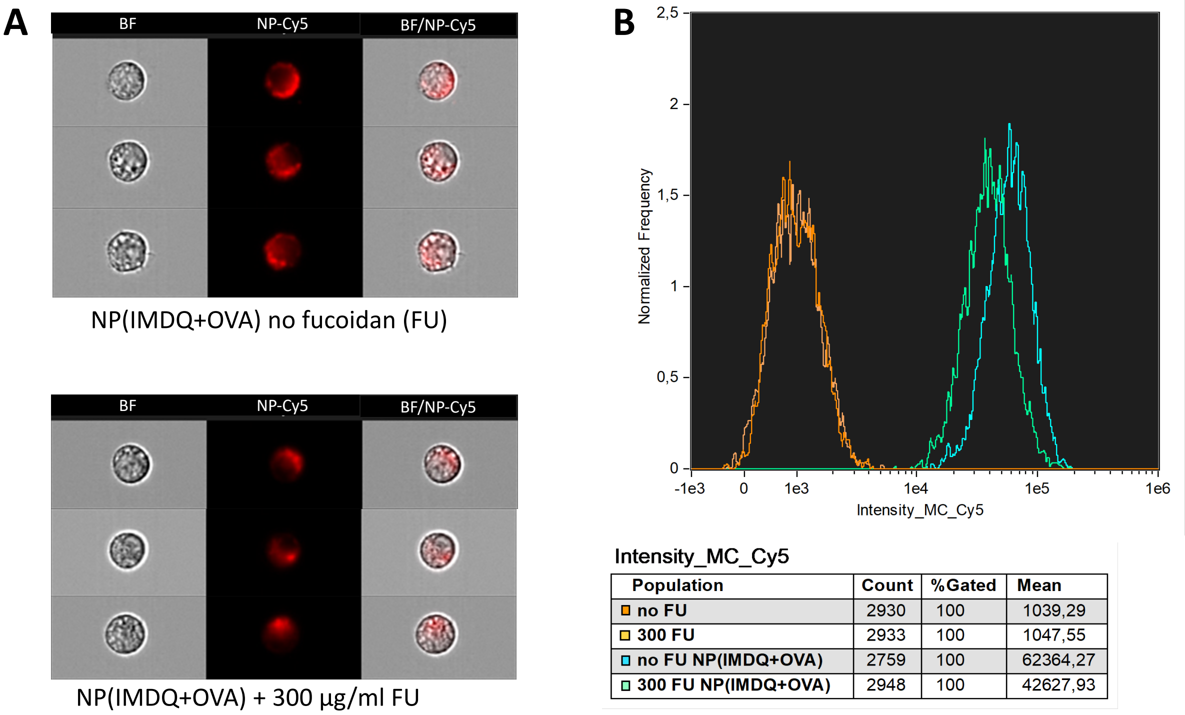

Population	Count	%Gated	Mean
no FU	2930	100	1039,29
300 FU	2933	100	1047,55
no FU NP(IMDQ+OVA)	2759	100	62364,27
300 FU NP(IMDQ+OVA)	2948	100	42627,93

Figure S46: Results of imaging flow cytometry of DCs incubated with NP(IMDQ+OVA) with and without 300 µg/mL fucoidan (FU). (A) ImageStream recorded images of bright field (BF), Cy5 derived NP fluorescence and overlay (magnification 40x). (B) Derived histogram for the samples with and without 300 µg/mL fucoidan (FU).

In vivo toxicity assay

To analyse the *in vivo* tolerance of the used nanoparticles, we immunized the mice intravenously (i.v.) with 100 µl of nanoparticles or soluble TLR agonist (corresponding to 10 µg of IMDQ, or 30 µg OVA) and took blood samples at 4, 8 and 24 h. After 48 h mice were sacrificed and blood was taken again. At each time point blood samples were analyzed on a scil Vet^{abc} Animal Blood Counter for parameters like numbers of white and red blood cells and platelets as indicators for homological toxicities (n=3).

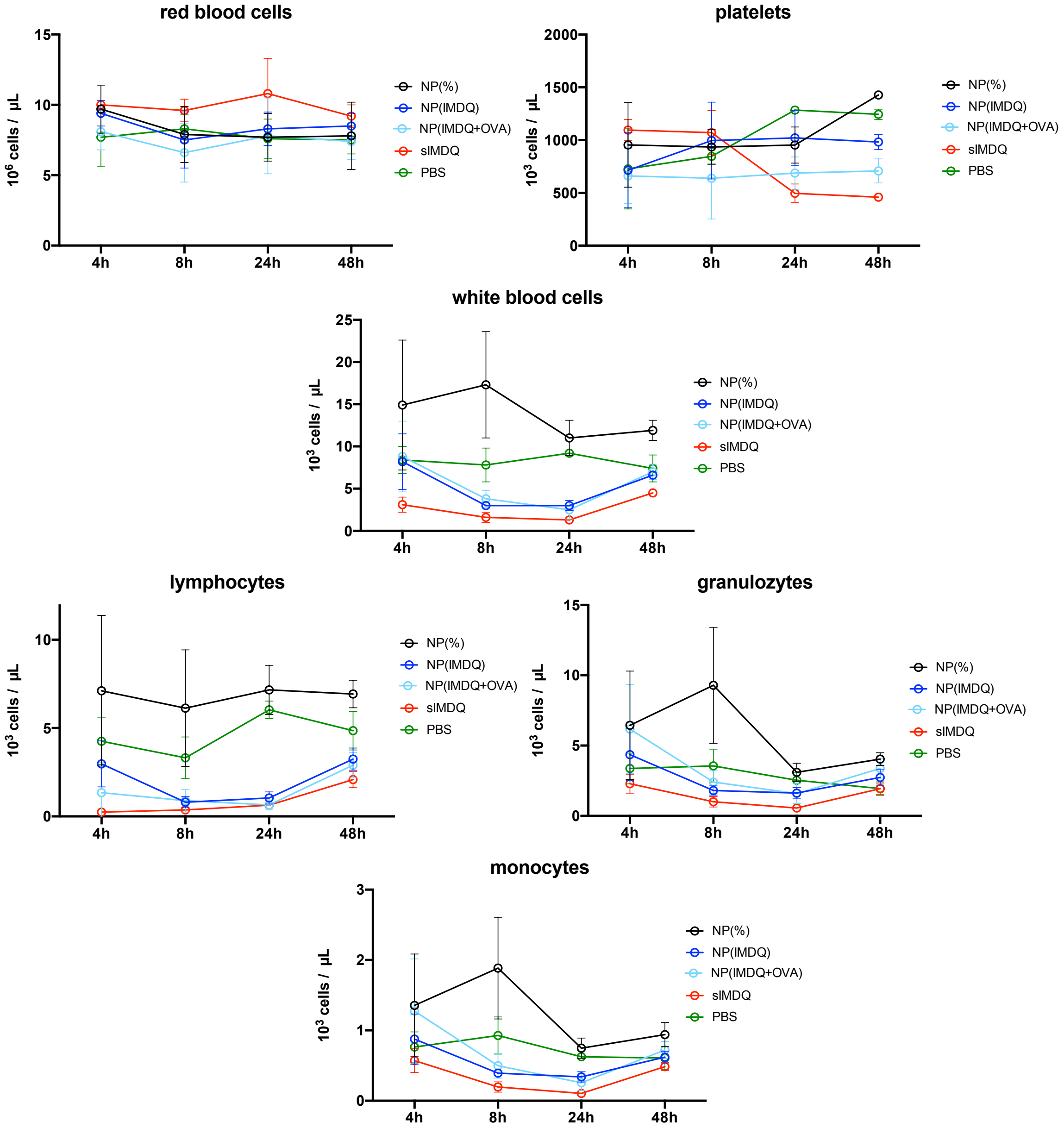

Figure S47: Hematologic toxicity analysis of empty nanogels, IMDQ-loaded nanogels, IMDQ- and OVA-loaded nanogels versus soluble IMDQ after single i.v. injection. While no effect was found on red blood cells, platelets are significantly most reduced sIMDQ. Same is found for white blood cells including lymphocytes, granulocytes and monocytes.

Biodistribution of IMDQ-loaded nanogels (IVIS + FACS)

To analyse the *in vivo* biodistribution of the used nanoparticles, BALB/c mice were injected intravenously via the tail vain with 100 µl of Cy5 labeled nanoparticles conjugated or mixed with OVA (NP(IMDQ+OVA) or NP(IMDQ)+sOVA) as well as a mixture of soluble TLR agonist with sOVA in PBS (n=3). For each sample a dose corresponding to 10 µg of IMDQ or 30 µg OVA was used. After 24 h, blood was taken from these mice by heart punction and analyzed for cytokine production as well as liver parameters with respect to liver related toxicities. Afterwards, organs were dissected and imaged by an IVIS Spectrum Imaging CT system (excitation/emission: 633/647nm; Caliper Life Sciences, Waltham, MA) in order to localize the distribution of the Cy5-labeled nanogels. Regions of interest (ROI) were drawn and mean fluorescence signal was measured within these ROIs for semi-quantitative analysis of the biodistribution (fluorescence intensities are presented as total radiant efficiency ([p/s]/[µW/cm^2]; attention should be drawn to the fact that quantification of Cy5 signal is restricted and can only be used for indicative comparison). From liver and spleen, single cell suspensions were prepared following standard protocols (liver non-parenchymal cells (NPC) were obtained by enzymatic/mechanical digestion (Liver Dissociation Kit; Miltenyi Biotec, Bergisch-Gladbach, Germany); spleen cell suspensions were generated by mechanical dissociation of the according organs).

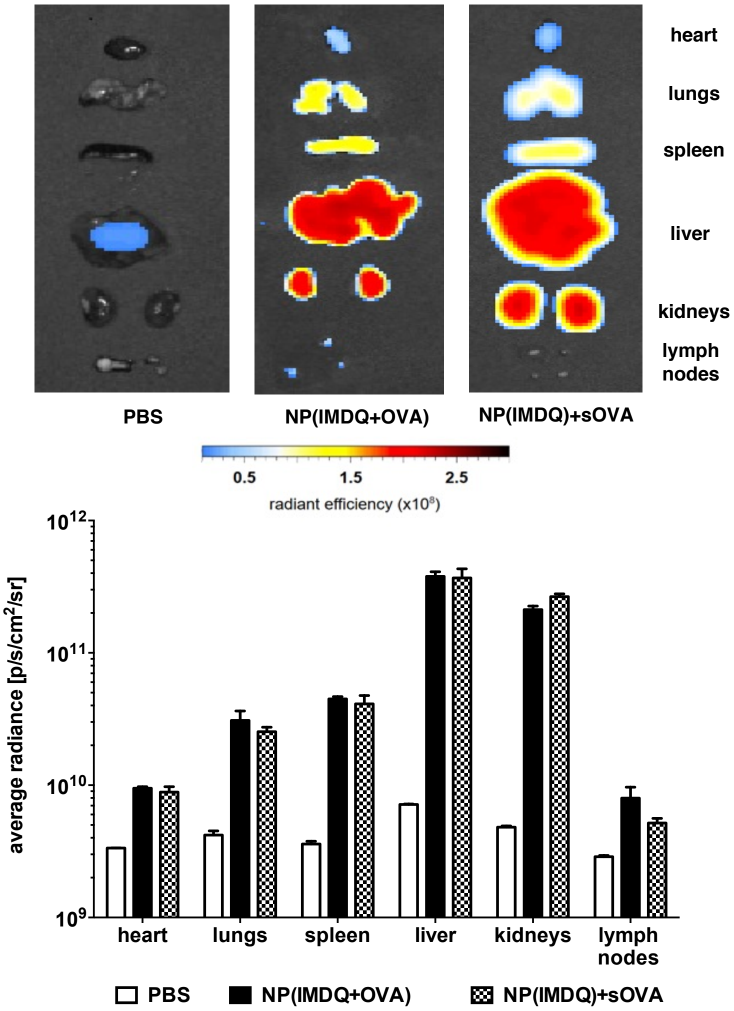

Figure S48: Biodistribution of IMDQ-loaded nanogels after i.v. injection. (A) Evaluation of organ-specific accumulation of IMDQ-loaded Cy5-labeled nanogels 24 h after i.v. injection. (B) Quantification of organ-specific accumulation of Cy5-labeled nanogels.

Liver enzyme activity

Blood that was retrieved by heart puncture was used for determining the serum contents for aspartate aminotransferase (AST), alanine aminotransferase (ALT), alkaline phosphatase (ALP), L-lactate dehydrogenase (LDH) and blood urea by nitrogen content (BUN). The parameters were measured by the central laboratory of the University Medical Center of Mainz.

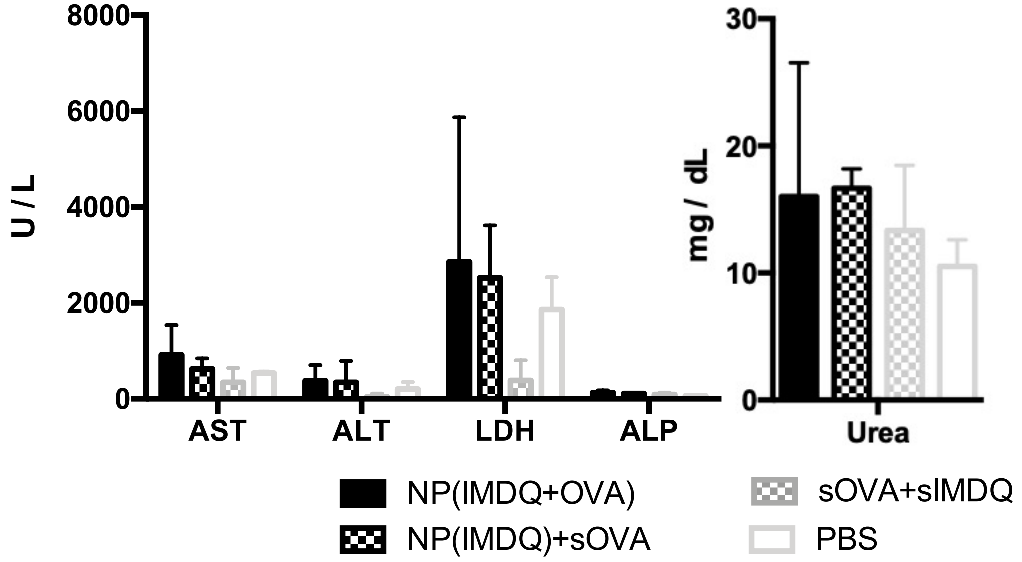

Figure S49: Liver toxicity parameters of IMDQ-loaded nanogels from blood 24h after i.v. injection. Note that the large variation of LDH activity is related to some hemolytic blood samples.

For additional insights into the immediate immune response, experiments were repeated and blood samples were collected 4 h and 24 h after i.v. injection of NP(IMDQ+OVA) or sIMDQ+sOVA. Again, liver enzyme parameter swere measured by the central laboratory of the University Medical Center of Mainz.

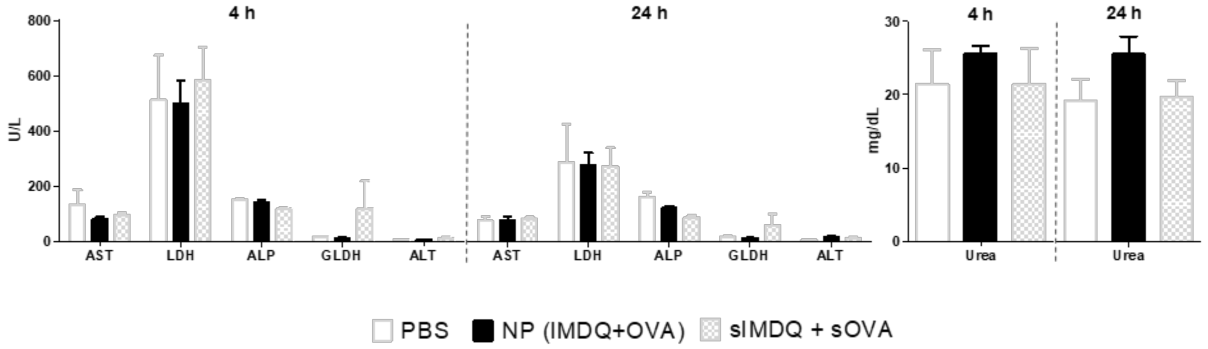

Figure S50: Liver toxicity parameters of IMDQ-loaded nanogels from blood 4 h and 24h after i.v. injection.

Cytokine analysis by cytometric bead array

Cytokine concentrations were determined by cytometric bead array (CBA; BD Biosciences, San Jose, CA) as recommended by the manufacturer. For this, bead populations with distinct fluorescence intensities were conjugated with cytokine-specific capture antibodies. Recombinant cytokines were used to prepare standard dilutions. Samples were mixed with capture beads, subsequently incubated with PE-conjugated detection antibodies for 1 h (all at room temperature in the dark) and subjected to flow cytometric analysis. Results were analyzed using FCAP Array Analysis Software v.1.0.1 (BD Biosciences).

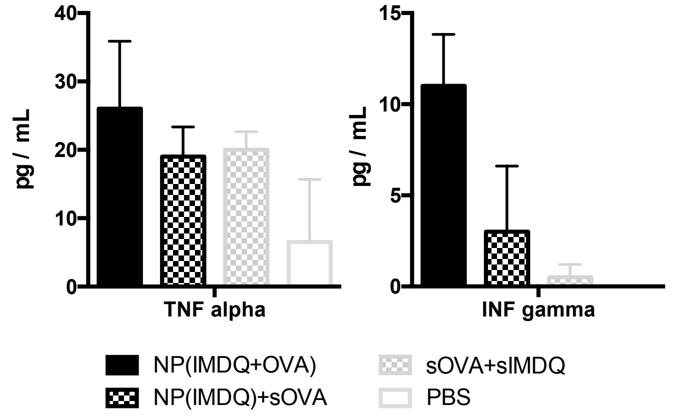

Figure S51: Cytokine parameters of IMDQ-loaded nanogels from blood 24h after i.v. injection.

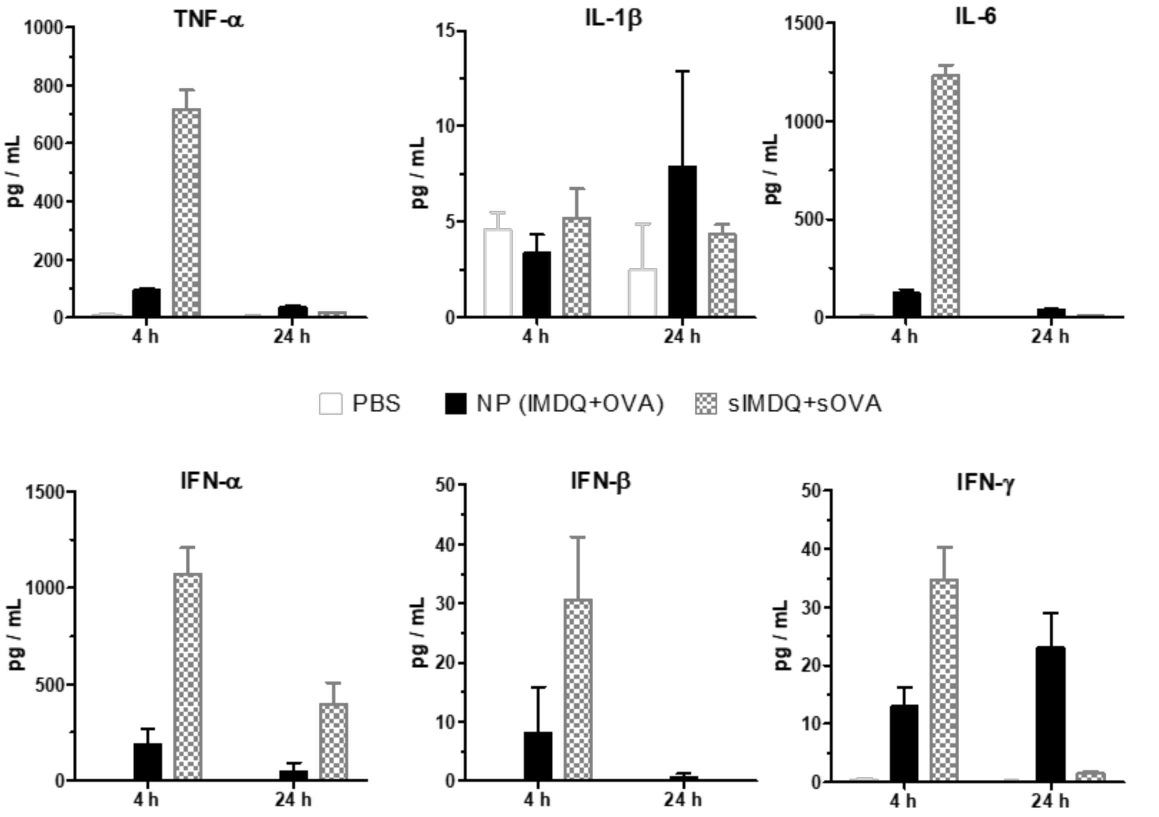

Figure S52: Cytokine parameters of IMDQ-loaded nanogels from blood 4 h and 24 h after i.v. injection measured by CBA.

Histopathological analysis by HE staining

Mice were injected with 100 ul either NP(IMDQ+OVA) or sIMDQ+sOVA, each containing 10 µg IMDQ and 30 µg OVA, i.v. in the tail vain. For histopathological analysis inner organs of mice (namely: heart, lungs, liver, spleen and kidneys) were retrieved after 24 h, paraffin-embedded blocks were prepared, and derived tissue sections (3 µm) were stained with haematoxylin & eosin (H&E) to assess inflammatory responses and tissue injury. To this end, H&E-stained tissue sections were examined by microscopy in a blinded fashion by an independent pathologist using a scoring system that quantifies the degree of tissue inflammation or tissue injury (0–3). In general, three randomly selected areas on each slide were analyzed with an Axioscope 5 equipped with a Axiocam 208 (Zeiss, Jena, Germany).

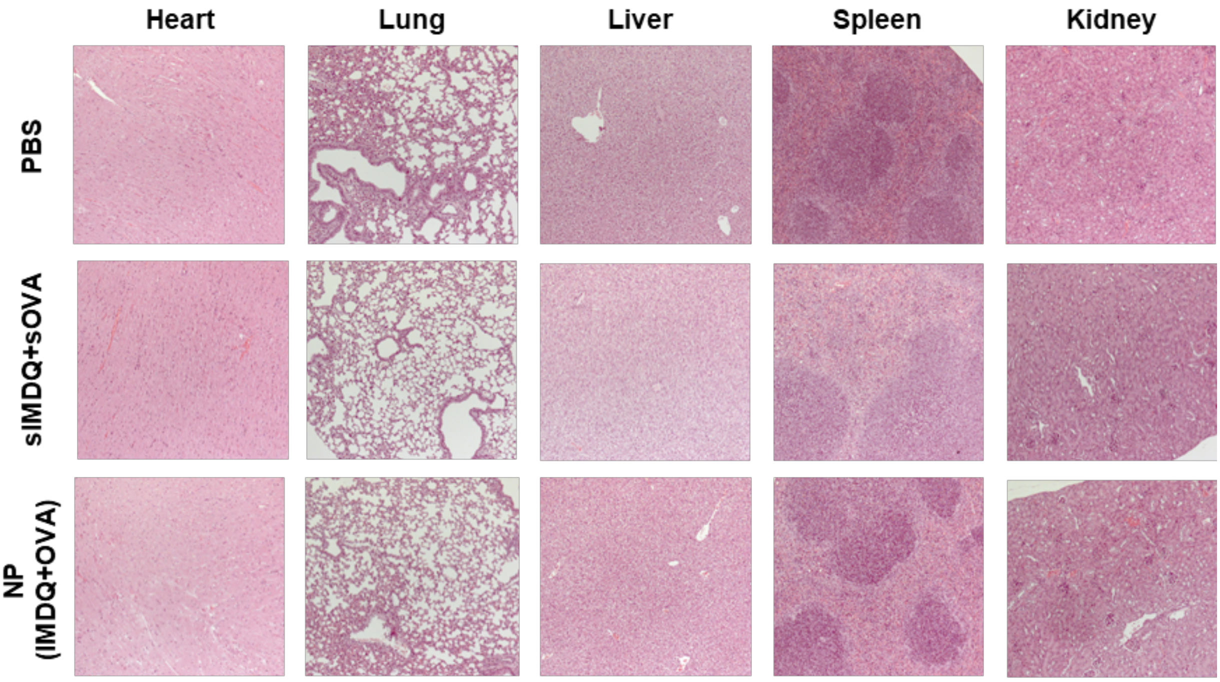

Figure S53: Histological analysis of various tissues by HE staining after iv injection of IMDQ- and OVA-containing formulations. No histological anomalies were observable for both samples.

Flow cytometry of liver and spleen cells

Liver and spleen single cell suspensions were incubated first with a rat anti-mouse CD16/CD32 (Fc-block) antibody (15 min, 4 °C) and washed with 2% FBS in PBS.
For analyzing liver non-parenchymal cells (NPC), they were further incubated for 20 min at 4 °C with antibodies specific for CD45 (BV711), CD32b (PE), CD11c (PE-Cy7), MHC-II (eFluor610, CD86 (eFluor450) and F4/80 (eFluor506).
For analyzing spleen cells, they were further incubated for 20 min at 4 °C with specific antibodies for CD3 (eFluor5069, CD19 (Super Bright 702), CD11b (Super Bright 600), CD11c (Brilliant Violet 421), MHC-II (PE), CD86 (PE-Cy7), Ly6G (eFluor610) and F4/80 (eFluor506).
All antibodies were obtained Thermo Fisher (Waltham, MA). After antibody incubation, samples were incubated with fixable viability dye (FVD) APC-eFluor780 (Thermo Fisher) to exclude dead cells. Washed samples were fixed with 0.7% PFA and subjected to flow cytometric analysis employing an Attune NxT flow cytometer (Thermo Fisher). The resulting data were processed by FlowJo software following the displayed gating strategy.

Gating Strategy in vivo biodistribution and immune activation in the liver

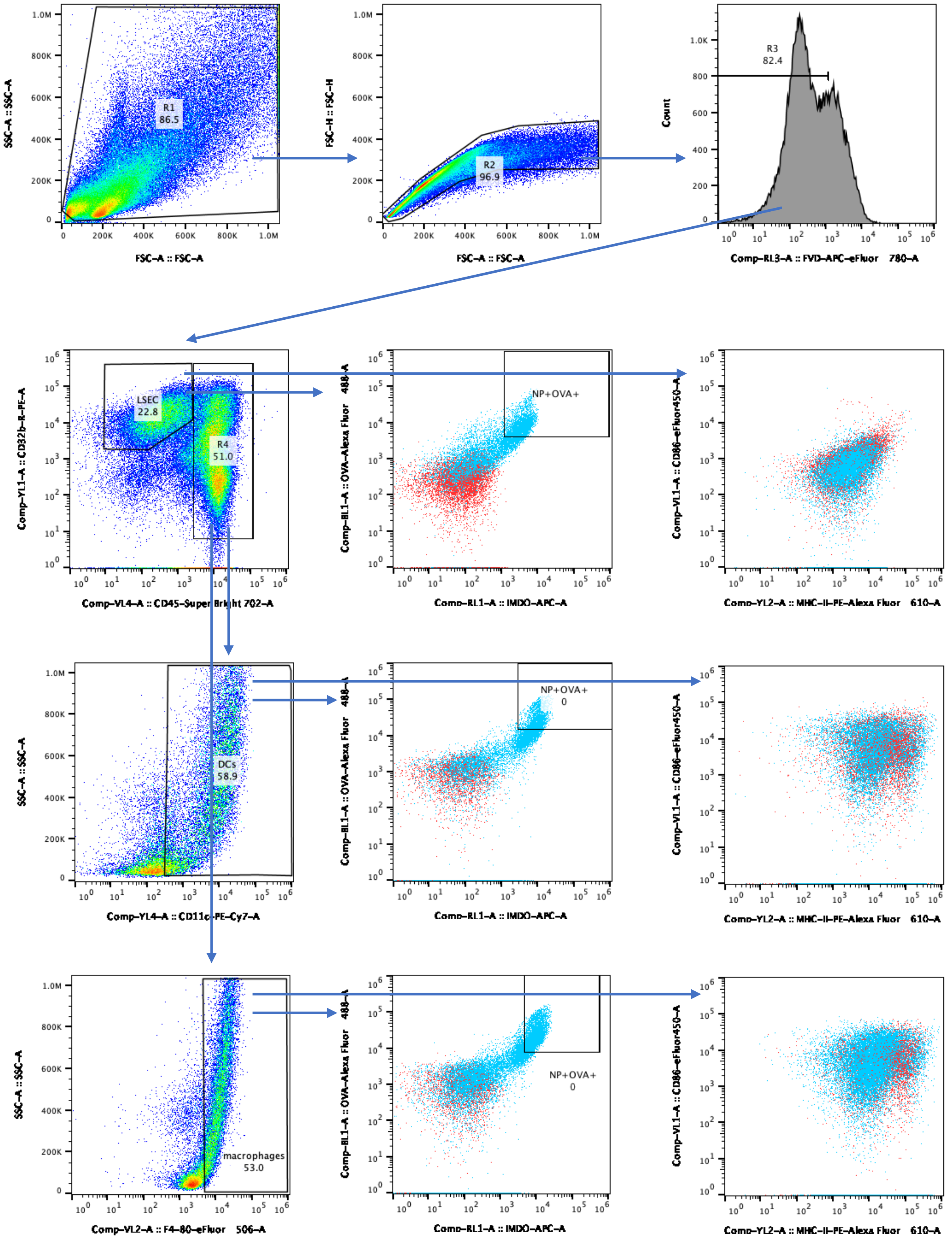

Figure S54: Gating strategy for flow cytometry analysis of liver single cell suspensions after 24 h treatment with NP(IMDQ+OVA), NP(IMDQ)+sOVA, sOVA+sIMDQ or PBS (n = 3).

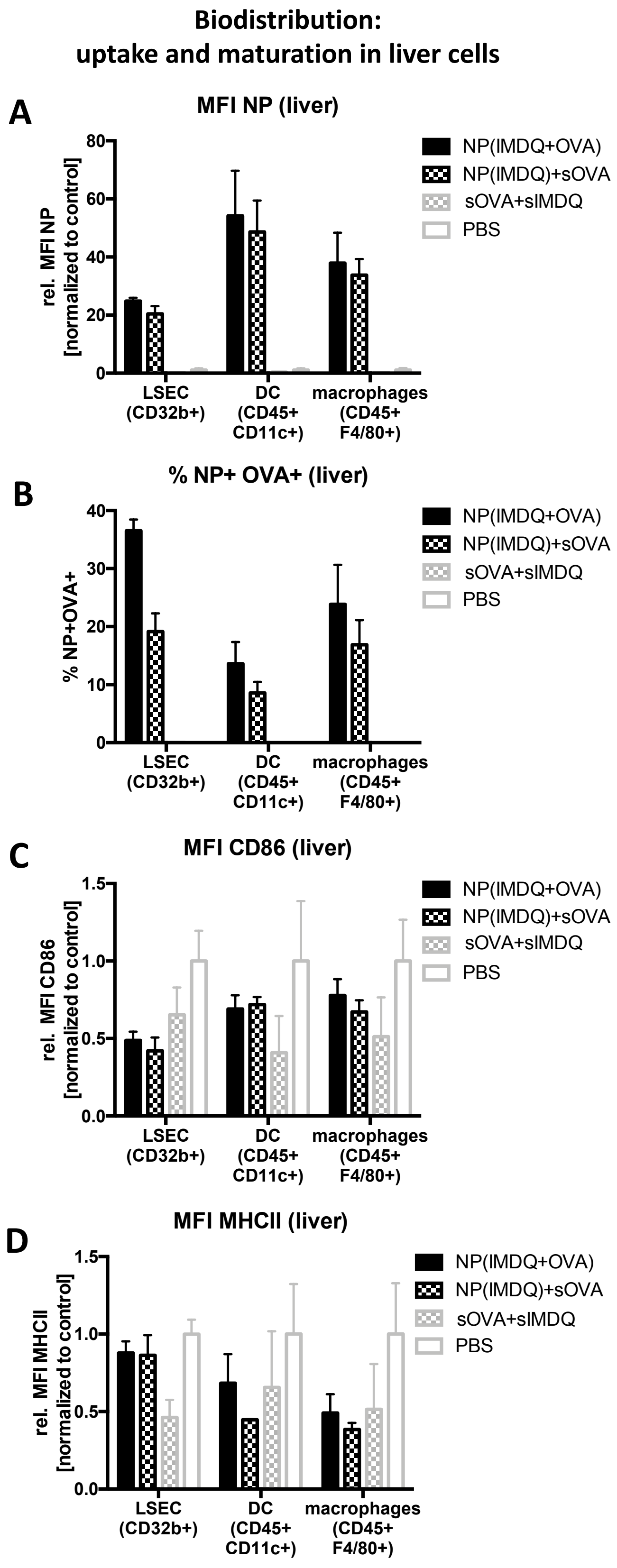

Figure S55: Results of particle uptake (A, relative MFI of Cy5-labeled nanogels in the corresponding liver associated immune cells and liver sinusoidal endothelial cells LSEC in relation to PBS treated cells), antigen co-delivery (B, Cy5-NP$^+$ and AF488-OVA$^+$), as well as relative mean fluorescence intensity (MFI) values of the maturation markers CD86 (C) and MHC-II (D) in relation to PBS treated mice. Note that particles can be found mostly in DCs and macrophages as antigen-presenting cells. A co-delivery of the antigen OVA works best for the covalent conjugate NP(IMDQ+OVA). However, none of the maturation markers CD86 and MHC-II can be upregulated compared to the PBS control, probably because of the immunosuppressive microenvironment in the liver.

Gating Strategy in vivo biodistribution and immune activation in the spleen

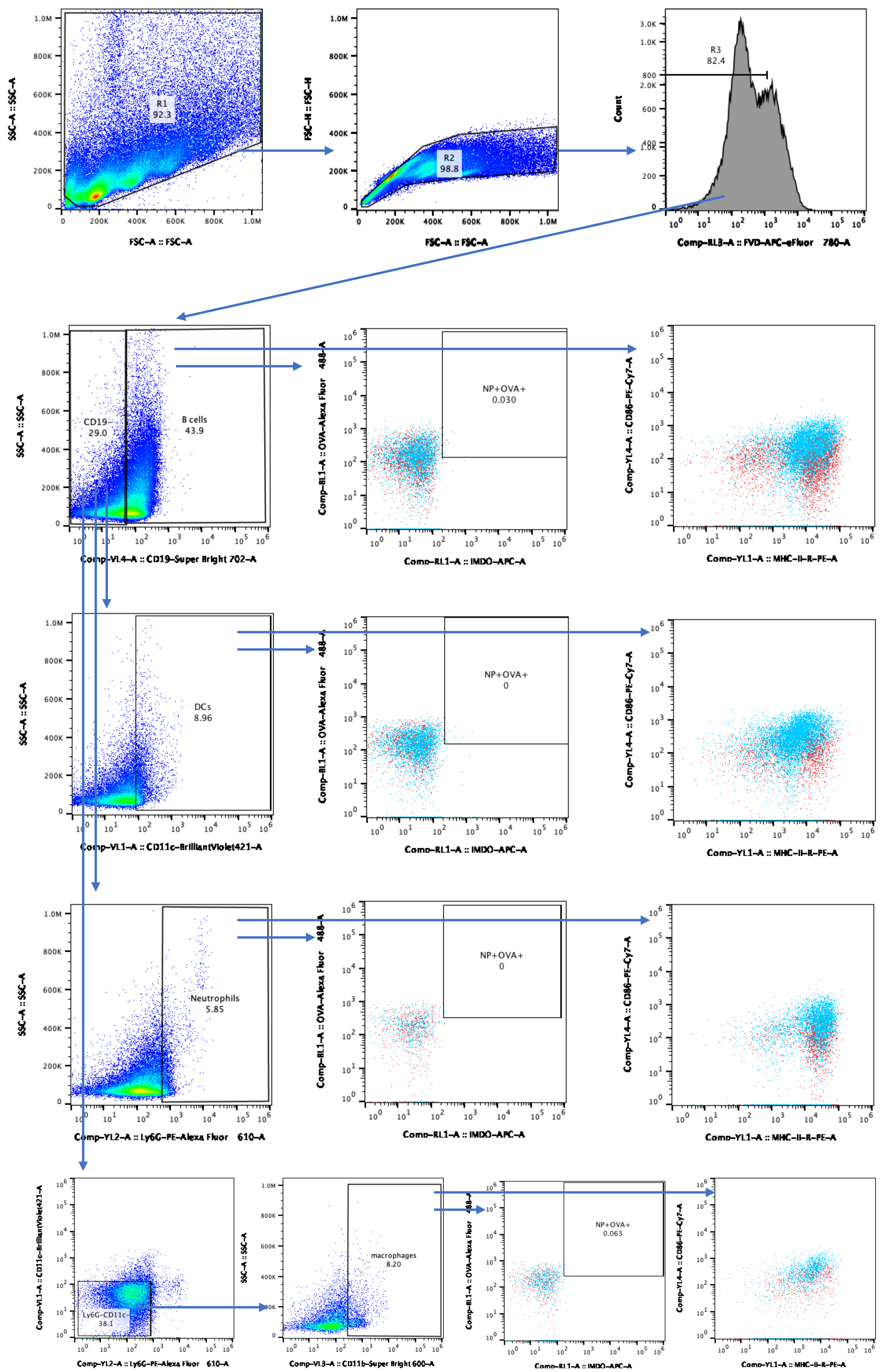

Figure S56: Gating strategy for flow cytometry analysis of spleen single cell suspensions after 24 h treatment with NP(IMDQ+OVA), NP(IMDQ)+sOVA, sOVA+sIMDQ or PBS (n = 3).

Biodistribution:
uptake and maturation in spleen cells

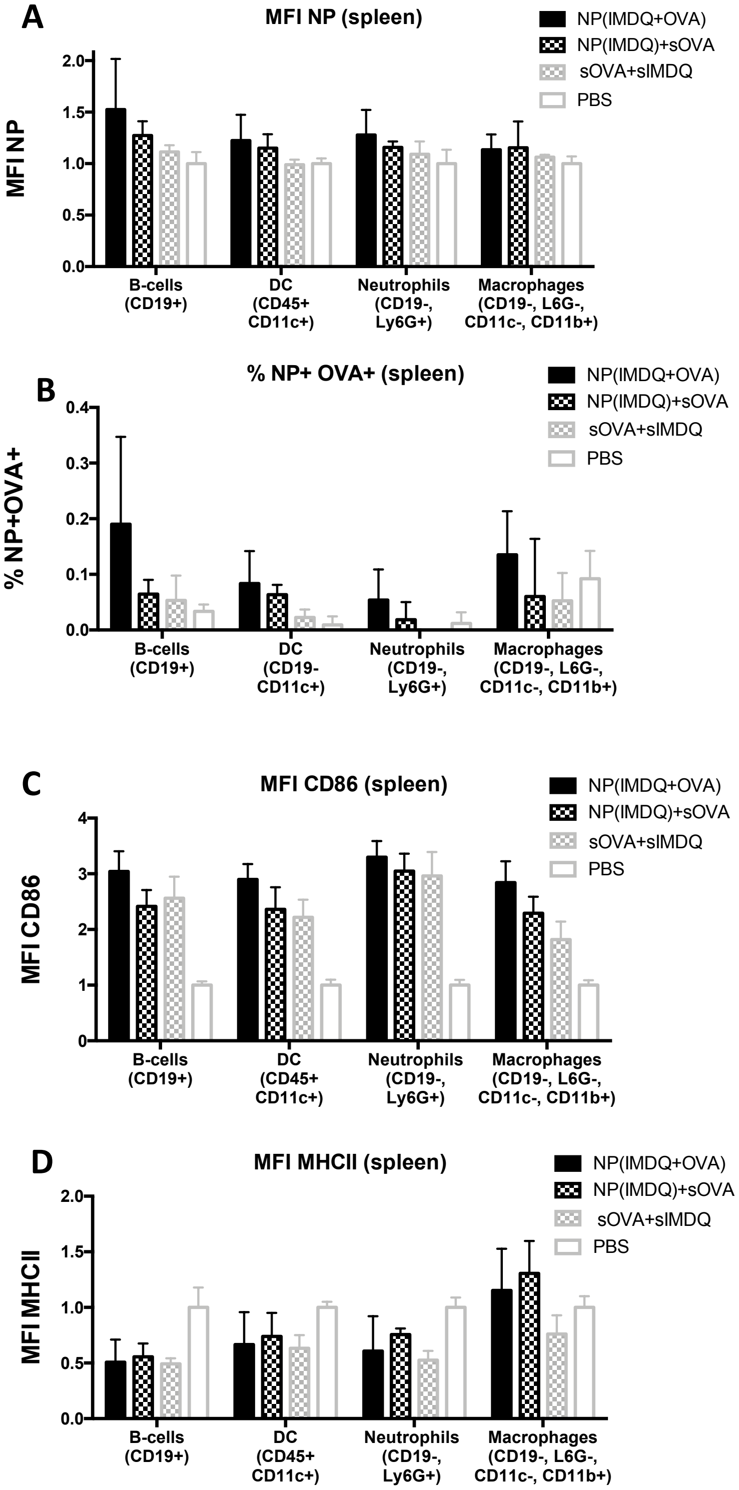

Figure S57: Results of particle uptake (A, relative MFI of Cy5-labeled nanogels in the corresponding antigen-presenting immune cells (APCs) in the spleen in relation to PBS treated cells), antigen co-delivery (B, Cy5-NP^{+} and AF488-OVA^{+}), as well as relative mean fluorescence intensity (MFI) values of the maturation markers CD86 (C) and MHC-II (D) in relation to PBS treated mice. Note that in analogy to the ex vivo experiments with splenocytes (Fig. S33 – S37) particles can be found mostly B-cells. A co-delivery of the antigen OVA works best for the covalent conjugate NP(IMDQ+OVA) into all spleen-associated APCs. Moreover, particle-bound IMDQ yields also the highest expression levels of the maturation maker CD86 – only the relative MFI values for MHC-II are again not very reliable, which is in accordance with Fig. S36 for the ex vivo experiments and may be related to the relatively high basal levels in the PBS control samples due to the applied splenic isolation conditions.

Immunization experiments

For immunization, 100 µl of nanoparticles (120 µg of nanoparticle, 10 µg IMDQ and 30 µg OVA respectively) were injected i.v. into the tail vein of the mice (n=3 for wild type mice and n=2 for *TLR7*$^{-/-}$ mice). 14 days later, the immunization was repeated the same way ("boost"). Mice were sacrificed on day 26 to analyse antibody titres in the blood and cytotoxic T cells in the spleen. The respective control groups received 100 µl PBS or unfunctionalized nanoparticles (NP(%)).

For a comparison of administration routes, nanoparticles were injected either s.c. or i.v. into the tail vein of mice as described before. Again, antibody titres were determined from blood by ELISA and T cells in the spleen by ELISpot analysis. In addition, body weight of mice was recorded each 3-4 days.

ELISA analysis

ELISA analyses were performed using sera from immunized mice before and after immunization with different kinds of nanoparticles. In brief, 96-well plates were coated with 25 mg/mL OVA at 4 °C over night. After blocking the 96-well plate, the serum was added 1:100 in the first well and titrated serially 1:2 over the plate. After 1 hour of incubation at 37 °C, detection of OVA-specific antibody production was performed using anti-IgG1-HRP, anti-IgG2a-HRP and anti-IgM-HRP antibodies (all received from Abcam, Cambridge, United Kingdom), which were incubated for 30 min at 37 °C. After washing the plate, 5 mg/mL ABTS was added and after 10 – 15 min of incubation at room temperature, the absorbance was measured at 410 nm. As a positive control, an anti-OVA-HRP antibody (Sigma Aldrich, St. Louis, Missouri, United States) was used.

ELISpot analysis

96-well plates (MultiScreenHTS IP, 0.45 mm, Merck Millipore, Darmstadt, Germany) were coated with 10 µg/mL of anti-IFNγ antibody (clone AN18, Mabtech, Nacka Strand, Sweden) at 4 °C over night. After blocking the plate, splenocytes were plated. Therefore, mice were sacrificed and the spleen was removed. After isolating the splenocytes by using a cell strainer and lysis of erythrocytes, cells were counted and plated at $5x10^5$ per well. They were restimulated with either 1 µM $OVA_{257-264}$, 1 µM $OVA_{323-337}$ or left untreated and incubated at 37 °C for 20 hours. After washing the plate, 2 mg/mL of a biotinylated anti-IFNγ antibody (clone R4-6A2, Mabtech) was added and incubated for 2 hours at 37 °C. Afterwards Vectastain ABC Kit (Vector Laboratories, Burlingame, USA) / AEC (Sigma-Aldrich, Taufkirchen, Germany)-Complex was added according to manufacturer 's constructions. After spots were visible plates were washed with water and dried overnight. Spot counting was performed on an AID iSpot ELISpot Reader (AID AutoimmunDiagnostika, Straßberg, Germany).

Tetramer and antibody staining

Peripheral blood samples were collected and incubated with the following antibodies: BV421conjugated anti-CD8, APC-conjugated anti-CD44 and FITC-conjugated anti-CD62L (all from eBioscience) after hypotonic lysis. H-$2K^b$-$OVA_{257-264}$-specific T cells were detected by H2-K^b tetramers containing $OVA_{257-264}$ peptides as described.[9] The samples were analyzed by flow cytometry.

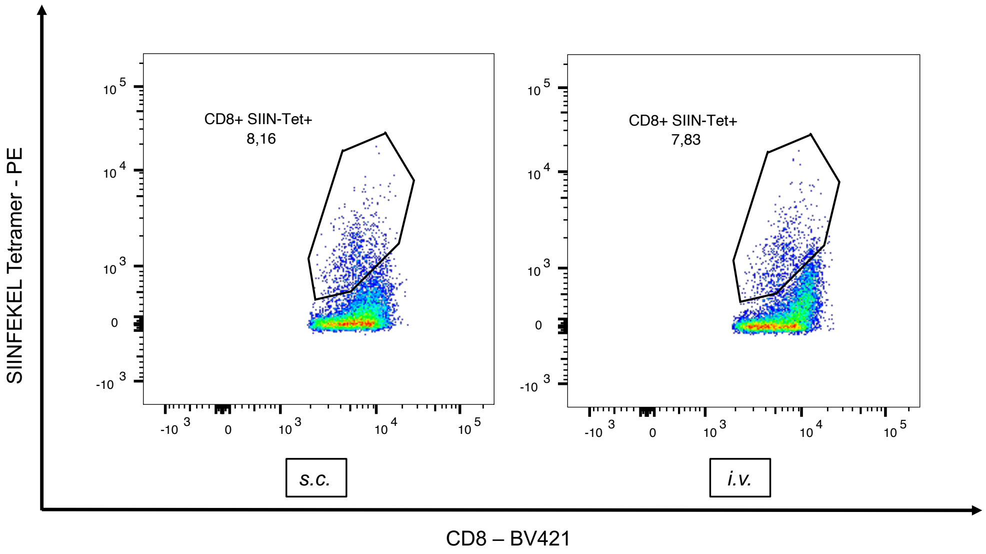

Figure S58: FACS Plots from flow cytometry gated for OVA-specific CD8⁺ T cells in blood after s.c. and i.v. injection of NP(IMDQ+OVA) determined by tetramer staining.

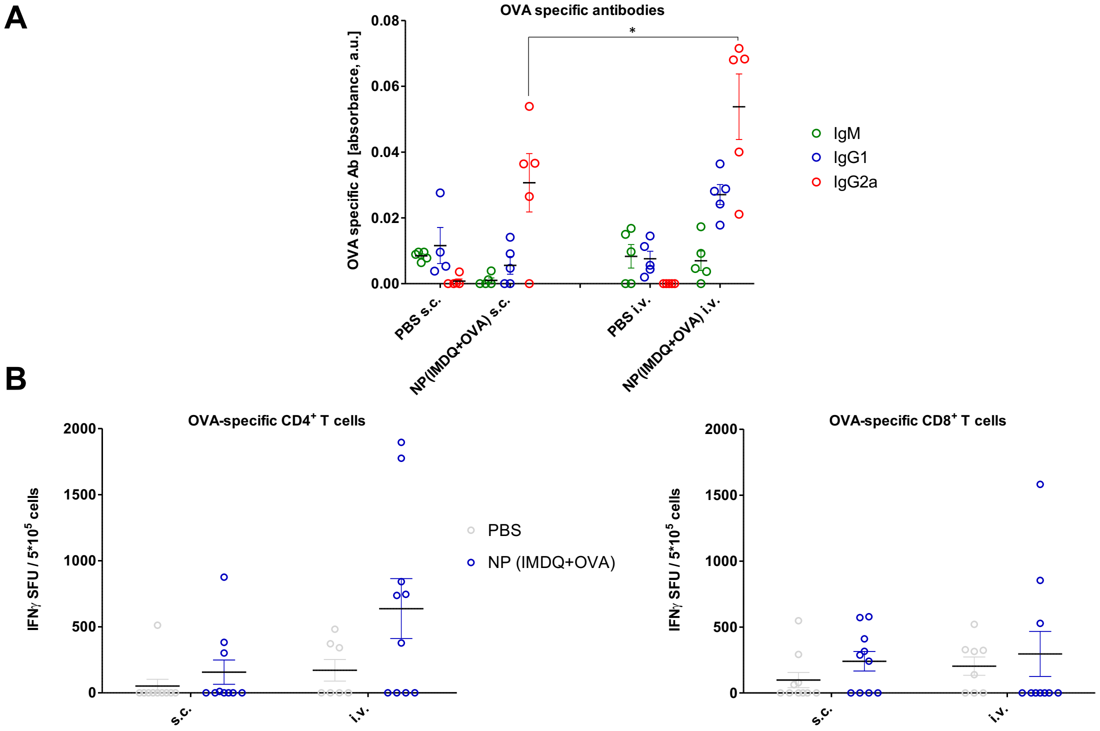

Figure S59: Humoral and cellular immune responses after s.c. and i.v. administration of NP(IMDQ+OVA). A) OVA specific antibodies determined by ELISA of the blood serum, (B) corresponding T cells determined by ELISpot of the isolated spleen cells of mice.

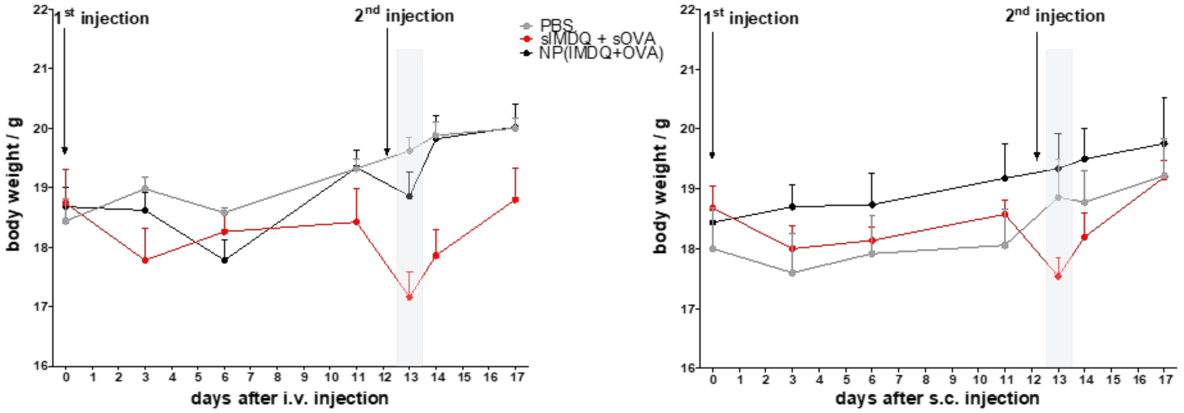

Figure S60: Body weight of wild type mice after i.v. (left) or s.c. (right) administration of IMDQ-containing formulations. Independent of the application route sIMDQ+sOVA lead to a drop in body weight after the boost injection and slower recovery.

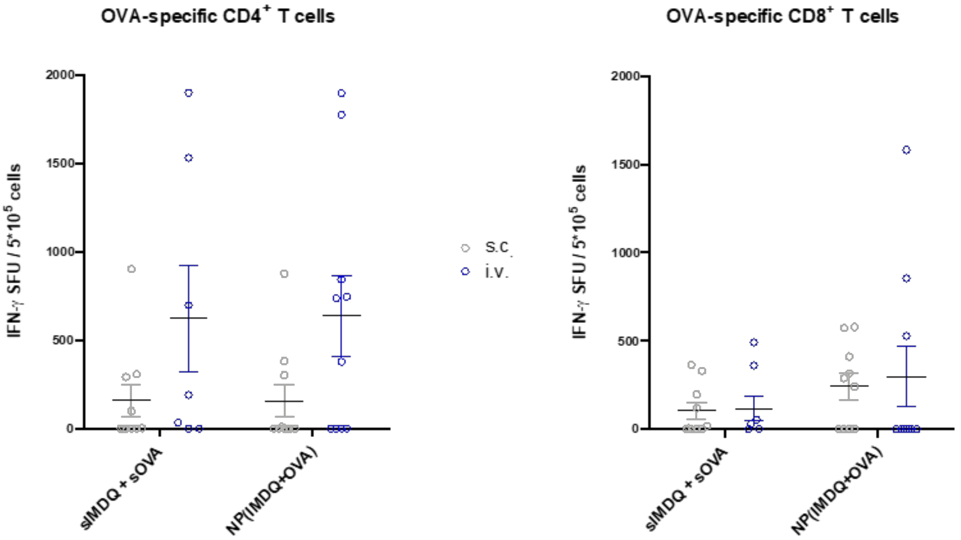

Figure S61: ELISpot analysis of on day 25 isolated spleen cells after i.v. or s.c. injection of sIMDQ+sOVA or NP(IMDQ+OVA).

Surface and cytosolic OVA expressing tumor cell line

To maintain EGFP and OVA expressions, transfected cells were cultured in media containing G418 (400 µg/mL for MC38mOVA, 1000 µg/mL for B16-F10mOVA and B16-F10cOVA). For surface OVA staining, MC38 cells, MC38 mock-transfected cells expressing only EGFP or MC38mOVA were incubated for 20 min at 4°C with a rabbit polyclonal antibody against OVA (dilution 1:300), which was kindly provided by H.-C. Probst (Institute for Immunology, University Medical Center, Johannes Gutenberg-University Mainz) and K. Schäkel (Department of Dermatology, University Hospital Heidelberg). After secondary antibody staining using a goat anti-rabbit IgG (H+L) conjugated to Alexa Fluor 647 (Thermo Fisher Scientific, dilution 1:500), data were acquired on an Amnis ImageStream Mk II flow cytometer (Luminex) at the Core Facility of the Research Center for Immunotherapy (University Medical Center, Johannes Gutenberg-University Mainz) and analysed using the IDEAS software (Luminex). Dead cells were gated out using DAPI (Thermo Fisher Scientific, dilution 1:10,000).

For recognizing and eliminating OVA-expressing cells by T cells, OT-I transgenic mice were used to generate $CD8^+$ T cells that respond to peptide 257-264 of OVA. Cytotoxicity activity was measured using the CytoTox96 Non-Radioactive Cytotoxicity Assay kit (Promega) and by following the manufacturer´s instructions. In brief, target cells were incubated with varying numbers of effector cells for 4 h, and supernatants were then analysed for lactate dehydrogenase release. The results are expressed as percent specific lysis, calculated as (Experimental release – Spontaneous release)/(Total release – Spontaneous release) x 100%.

In vivo tumor experiments

In therapeutic tumor experiments, 8-12 weeks old mice were subcutaneously (s.c.) inoculated either with 5x10^5 MC38mOVA cells, B16-F10mOVA cells or 1x10^5 B16-F10cOVA in 100 µl PBS into the right flank. In each experiment the same number of corresponding parental tumor cells were inoculated as control. On day 3, 5 and 7 after tumor inoculation, 100 µl of nanoparticles or PBS were i.v. injected into the tail vein of the mice. Tumor growth was observed over a time period of 17-19 days. Tumor size was measured every 2-3 days using a caliber and tumor volume was calculated by the formula width^2 x length x 0,5. Mice were sacrificed when the tumor volume reached more than 600mm^3 or when there was external necrosis.
In a prophylactic tumor model, the mice first received 100 µl of nanoparticles i.v. into the tail vein on day 0 and day 14 and the tumor cells were inoculated on day 23. From now on, the experimental procedure was the same as described for the therapeutic tumor models.

ELISA, ELISpot and cytokine analysis of tumor bearing mice

ELISA and cytokine analysis of the B16-F10 in vivo tumor experiments were performed with blood sera from mice on day -1 (so before tumor inoculation and treatment with the nanogel) and day 13 after tumor inoculation. For ELISA, same protocols were followed as described above, while for cytokine analysis they were determined by cytometric bead assay according to the manufacturer (BD, Cytometric Bead Array Soluble Protein Flex Set). Diluted samples (1:4) were measured with a Attune NxT flow cytometer (Thermo Fisher) and analyzed after filtering with the FCAP FCS filter v.1.0.4.@2005-2008, soft flow with the FCAP Array software v.1.0.1.@2006, soft flow. For ELISpot analysis, splenocytes were isolated from the mice on day 13 after tumor inoculation and analyzed by the protocols described above.

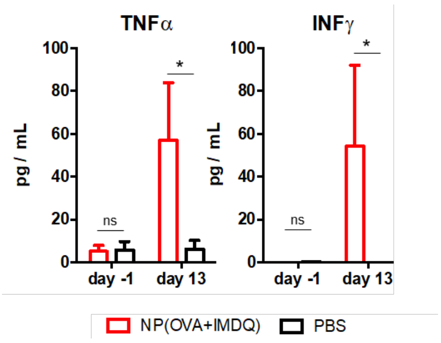

Figure S62: Cytokine analysis of blood serum samples taken on day -1 and day 13 after tumor inoculation from B16-F10mOVA bearing mice treated with NP(IMDQ+OVA) or PBS.

Flow cytometry of tumor cells

From MC38mOVA tumors treated with PBS or NP(IMDQ+OVA), cell suspensions were prepared following standard protocols, then transferred into sterile FACS tubes and incubated first with a rat anti-mouse CD16/CD32 (Fc-block) antibody (15 min, 4 °C). For analyzing myeloid cells, they were further incubated for 20 min at 4 °C with antibodies specific for CD45 (BV421), CD11c (PE-Cy7), CD11b (PE), MHC-II (BV510), Gr-1 (AF488) and F4/80 (APC-A). For T-cell analysis, they were incubated with antibodies specific for CD45 (BV421), TCR

(PE-Cy5), CD4 (BV711) or CD8 (BV510). All antibodies were obtained from BioLegend (San Diego, CA) or Thermo Fisher (Waltham, MA). After antibody incubation, samples were incubated with fixable viability dye (FVD) APC-Cy7 (Thermo Fisher) to exclude dead cells. Washed samples were fixed and subjected to flow cytometric analysis employing an Attune NxT flow cytometer (Thermo Fisher). The resulting data were processed by FACSDiva V6.0 software following the displayed gating strategy. All samples were run at n = 6.

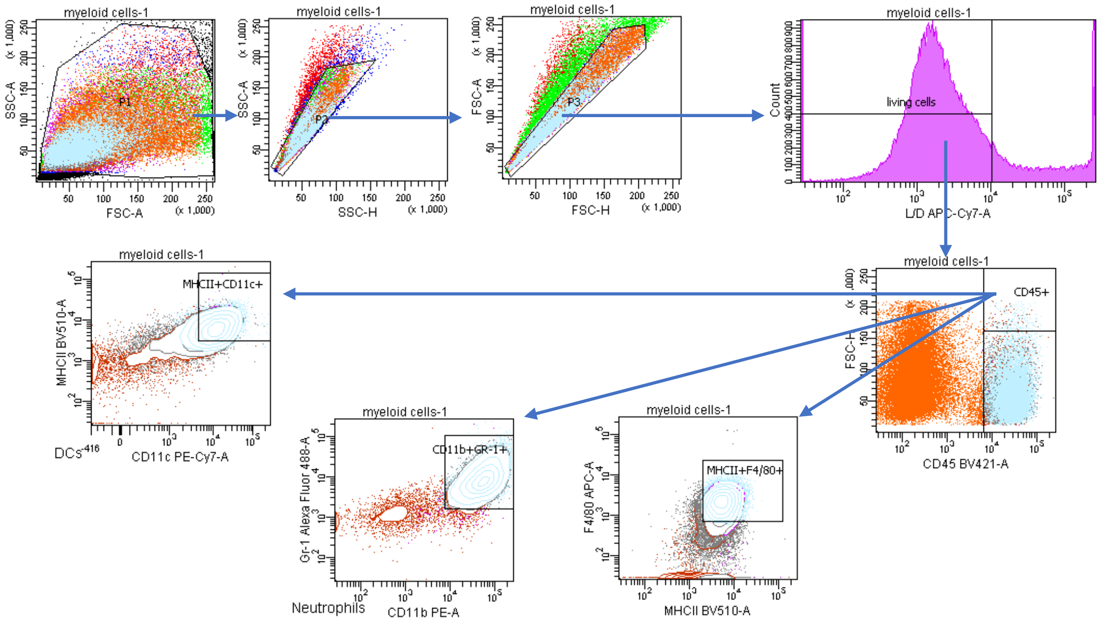

Figure S63: Gating strategy for flow cytometry analysis of myeloid cells from tumor single cell suspensions after treatment with NP(IMDQ+OVA) or PBS (n = 6).

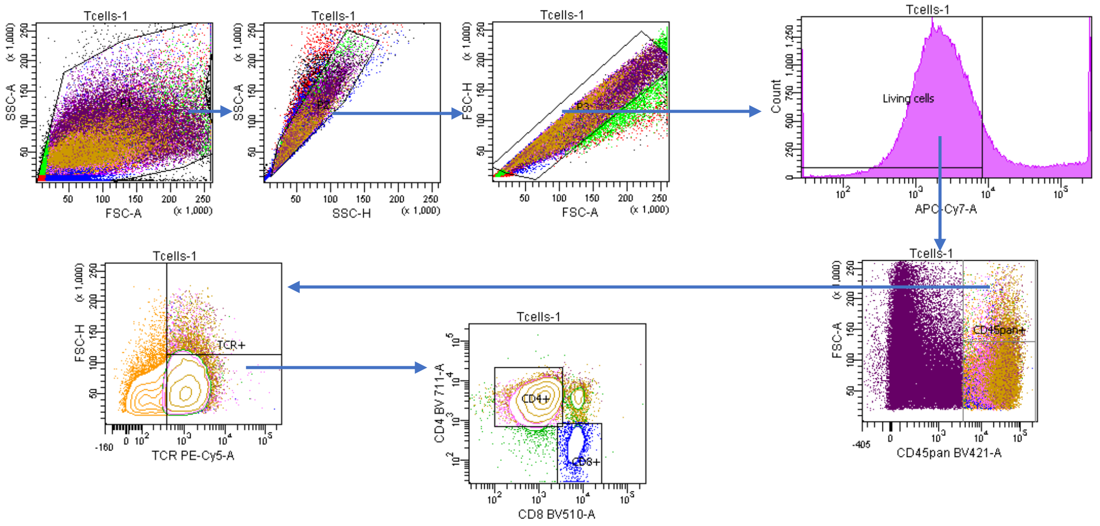

Figure S64: Gating strategy for flow cytometry analysis of T cells from tumor single cell suspensions after treatment with NP(IMDQ+OVA) or PBS (n = 6).

IgMi mice tumor experiments

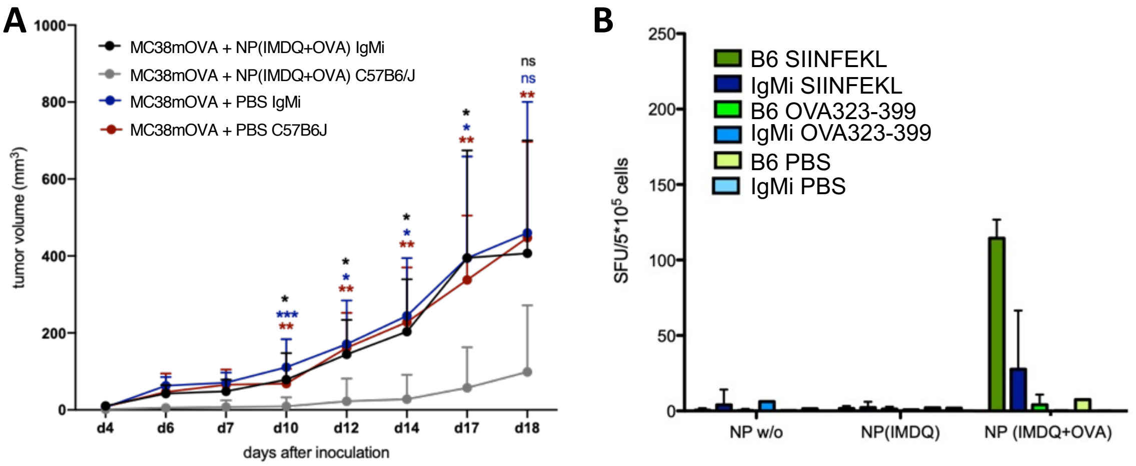

*Figure S65: Nanogel NP(IMDQ+OVA) vaccination in IgMi and wild type B6 (C57BL/6J) mice. (**A**) Mice were also challenged with MC38mOVA tumors but only in the wild type B6-mice a significant reduction in tumor growth was observed. (**B**) ELISpot analysis of spleens of IgMi and wild type B6 mice immunized with NP(IMDQ+OVA), NP(IMDQ) and PBS. A strong induction of OVA-specific CD8^{+} T cells was found in the in the wild type B6-mice, while in the IgMi group this number was already reduced. This further explains the low antigen-specific antitumor response in this mouse model challenged with MC38mOVA.*

Statistical analyses

Data are shown as mean values + standard deviation unless otherwise specified in the figure legend. To calculate statistical significance of the mean values student's t-test with Welch's correction was performed. For the tumor as well as mice experiments and the corresponding measurements (ELISA, ELISpot) the 2-way-ANOVA test was used. All statistical analyses were done using the graph pad prism 8 software. In all figures * = P ≤ 0.05; ** = P ≤ 0.01; *** = P ≤ 0.001 or otherwise mentioned.

References

(1) Shukla, N. M.; Malladi, S. S.; Mutz, C. A.; Balakrishna, R.; David, S. A. Structure−Activity Relationships in Human Toll-Like Receptor 7-Active Imidazoquinoline Analogues. *J. Med. Chem.* **2010**, *53* (11), 4450–4465.

(2) Leber, N.; Kaps, L.; Yang, A.; Aslam, M.; Giardino, M.; Klefenz, A.; Choteschovsky, N.; Rosigkeit, S.; Mostafa, A.; Nuhn, L.; et al. α-Mannosyl-Functionalized Cationic Nanohydrogel Particles for Targeted Gene Knockdown in Immunosuppressive Macrophages. *Macromol. Biosci.* **2019**, *1900162*, 1–12.

(3) Nuhn, L.; Kaps, L.; Diken, M.; Schuppan, D.; Zentel, R. Reductive Decationizable Block Copolymers for Stimuli-Responsive MRNA Delivery. *Macromol. Rapid Commun.* **2016**, *37* (11), 924–933.

(4) Nuhn, L.; Bolli, E.; Massa, S.; Vandenberghe, I.; Movahedi, K.; Devreese, B.; Van Ginderachter, J. A.; De Geest, B. G. Targeting Protumoral Tumor-Associated Macrophages with Nanobody-Functionalized Nanogels through Strain Promoted Azide Alkyne Cycloaddition Ligation. *Bioconjug. Chem.* **2018**, *29* (7), 2394–2405.

(5) Nuhn, L.; Vanparijs, N.; De Beuckelaer, A.; Lybaert, L.; Verstraete, G.; Deswarte, K.; Lienenklaus, S.; Shukla, N. M.; Salyer, A. C. D.; Lambrecht, B. N.; et al. PH-Degradable Imidazoquinoline-Ligated Nanogels for Lymph Node-Focused Immune Activation. *Proc. Natl. Acad. Sci.* **2016**, *113* (29), 8098–8103.

(6) Rigler, R.; Elson, E. S. *Fluorescence Correlation Spectroscopy - Theory and Applications*; Springer: Heidelberg, 2001.

(7) Nuhn, L.; De Koker, S.; Van Lint, S.; Zhong, Z.; Catani, J. P.; Combes, F.; Deswarte, K.; Li, Y.; Lambrecht, B. N.; Lienenklaus, S.; et al. Nanoparticle-Conjugate TLR7/8 Agonist Localized Immunotherapy Provokes Safe Antitumoral Responses. *Adv. Mater.* **2018**, *30* (45), 1803397.

(8) Nuhn, L.; Van Hoecke, L.; Deswarte, K.; Schepens, B.; Li, Y.; Lambrecht, B. N.; De Koker, S.; David, S. A.; Saelens, X.; De Geest, B. G. Potent Anti-Viral Vaccine Adjuvant Based on PH-Degradable Nanogels with Covalently Linked Small Molecule Imidazoquinoline TLR7/8 Agonist. *Biomaterials* **2018**, *178*, 643–651.

(9) Bialojan, A.; Sohl, J.; Rausch, J.; Aranda Lopez, P.; Denny, M.; Langguth, P.; Hartmann, A.; Yagita, H.; Probst, H. C.; Schild, H.; et al. Transcutaneous Immunization with CD40 Ligation Boosts Cytotoxic T Lymphocyte Mediated Antitumor Immunity Independent of CD4 Helper Cells in Mice. *Eur. J. Immunol.* **2019**, *49* (11), 2083–2094.
